# Hypoxia Induced *Lnc191* Upregulation Dictates the Progression of Esophageal Squamous Cell Carcinoma by Activating GRP78/ERK Pathway

**DOI:** 10.1002/advs.202406674

**Published:** 2024-12-04

**Authors:** Sisi Wei, Xinyi Fan, Xiaoya Li, Wei Zhou, Zhihua Zhang, Suli Dai, Huilai Lv, Yueping Liu, Baoen Shan, Lianmei Zhao, Qimin Zhan, Yongmei Song

**Affiliations:** ^1^ State Key Laboratory of Molecular Oncology National Cancer Center/National Clinical Research Center for Cancer/Cancer Hospital Chinese Academy of Medical Sciences and Peking Union Medical College Beijing 100021 China; ^2^ Research Center the Fourth Hospital of Hebei Medical University Jiankang Road 12 Shijiazhuang Hebei 050011 China; ^3^ Key Laboratory of Tumor Prevention and Precision Diagnosis and Treatment of Hebei Clinical Oncology Research Center Shijiazhuang Hebei 050011 China; ^4^ Hangzhou Institute of Medicine University of Chinese Academy of Sciences (Zhejiang Cancer Hospital) Hangzhou Zhejiang 310022 China; ^5^ Neurosurgery Department Tsinghua University Yuquan Hospital Beijing 100049 China; ^6^ Department of Thoracic Surgery the Fourth Hospital of Hebei Medical University Shijiazhuang Hebei 050011 China; ^7^ Pathology Department the Fourth Hospital of Hebei Medical University Shijiazhuang Hebei 050011 China; ^8^ Key Laboratory of Carcinogenesis and Translational Research Laboratory of Molecular Oncology Peking University Cancer Hospital & Institute Beijing 100142 China

**Keywords:** EGFR, ESCC, GRP78, LncRNA, MAPK

## Abstract

Hypoxia is a typical hallmark of solid tumors and plays a crucial role in the progression of esophageal squamous cell carcinogenesis (ESCC). Nevertheless, the precise mechanisms underlying the involvement of hypoxia in tumor development remain unclear. In the present study, a novel hypoxia‐induced long noncoding RNA (lncRNA) is identified first, *lnc191*, which is highly expressed in clinical ESCC tissues and is positively correlated with poor prognosis of ESCC patients. These findings provide evidence that the hypoxia‐inducible factor‐1α (HIF‐1α)‐mediated transcriptional activation of *lnc191* enhances the growth and metastasis of ESCC cells both in vitro and in vivo. Mechanistically, *lnc191* interacts with GRP78 (78‐kDa glucose‐regulated protein), one of the endoplasmic reticulum chaperone proteins, leading to its translocation to the membrane, where GRP78 binds with EGFR and enhances its phosphorylation (Y845), further activates ERK/MAPK signaling pathway, and thereby in favor of the progression of ESCC. Overall, this data proposes *lnc191* as a key driver during the development of ESCC and reveals the participation of the activated GRP78/ERK/MAPK axis in the ESCC progression mediated by *lnc191*. These findings indicate the potential of *lnc191* as a promising diagnostic biomarker and therapeutic target in ESCC.

## Graphical abstract

This study reveals that under hypoxic conditions, HIF‐1α transcriptionally activates *lnc191*, which in turn facilitates the progression of ESCC by directly binding to GRP78. Subsequently, *lnc191* expedites the translocation of GRP78 to the cell membrane, where it forms a complex with EGFR, ultimately leading to the Tyr845 phosphorylation of EGFR and activation of the MAPK/ERK pathway.

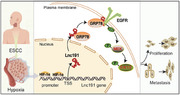

## 1 Introduction

Esophageal cancer (EC) is a frequently diagnosed malignancy, ranking sixth in global cancer deaths.^[^ ^1^, ^2^ ^]^ Esophageal squamous cell carcinoma (ESCC) is the main pathological type of EC in Asian and African countries, especially China, where it accounts for more than 90%.^[^ ^3^ ^]^ At present, ESCC is characterized by its aggressive phenotype, resulting in a 5‐year survival rate of less than 25%.^[^ ^4^, ^5^ ^]^ Therefore, there is a pressing need to better understand ESCC's molecular mechanisms and to develop effective diagnostic and treatment methods.

Hypoxia is a notable characteristic of solid tumors, primarily attributed to their rapid proliferation and inadequate angiogenesis.^[^ ^6^ ^]^ This condition triggers the extensive release of bioactive mediators, chemokines, and angiogenic factors, thereby facilitating the advancement and metastases of cancer.^[^ ^7^, ^8^ ^]^ Upon exposure to hypoxic stimuli, the expression of transcription factor HIF‐1α is significantly increased and binds to the hypoxia response elements (HREs) of many target genes,^[^ ^9^ ^]^ which promote the metastasis, proliferation, metabolism, and stemness of cancer cells.^[^ ^10^, ^11^ ^]^ Consequently, it is crucial to uncover novel molecular mechanisms that underlie the persistence of hypoxic tumors and thus overcome the hypoxia‐mediated microenvironment of tumor progression.

Accumulating evidence has shown that hypoxia modulates the expression of long noncoding RNAs (lncRNAs)^[^ ^12^ ^]^ and garner increasing attention for their involvement in cancer development.^[^ ^13^, ^14^ ^]^ Several lncRNAs, including STEAP3‐AS1, LUCAT1 and lincRNA‐p21, have been identified as being activated by HIF‐1α, thus contributing to tumor progression.^[^ ^15^, ^16^, ^17^ ^]^ Nevertheless, there is currently a lack of characterization for specific lncRNA that responds to hypoxia in facilitating ESCC development.

In this study, we systematically identified lncRNA sets that induced upregulation in hypoxia in ESCC cells through transcriptomic analysis. One of these lncRNAs, *lnc191*, was identified to be highly expressed under hypoxia conditions and in ESCC clinical specimens. Functional studies revealed that *lnc191* is of fundamental importance for the proliferation, migration, and invasion of ESCC cells both in vitro and in vivo and predicted a poor prognosis. Mechanically, *lnc191* interacted with GRP78 and facilitated its translocation to cell membrane, thereby activating the ERK/MAPK signaling pathway by enhancing the phosphorylation of EGFR, and promoting ESCC progression. Taken together, our findings revealed a new lncRNA involved in hypoxia‐induced ESCC and clarified the novel potential mechanism for EGFR‐mediated ESCC progression.

## 2 Results

### 2.1 *Lnc191* is Significantly Upregulated Under Hypoxic Condition in ESCC

**Figure 1:**
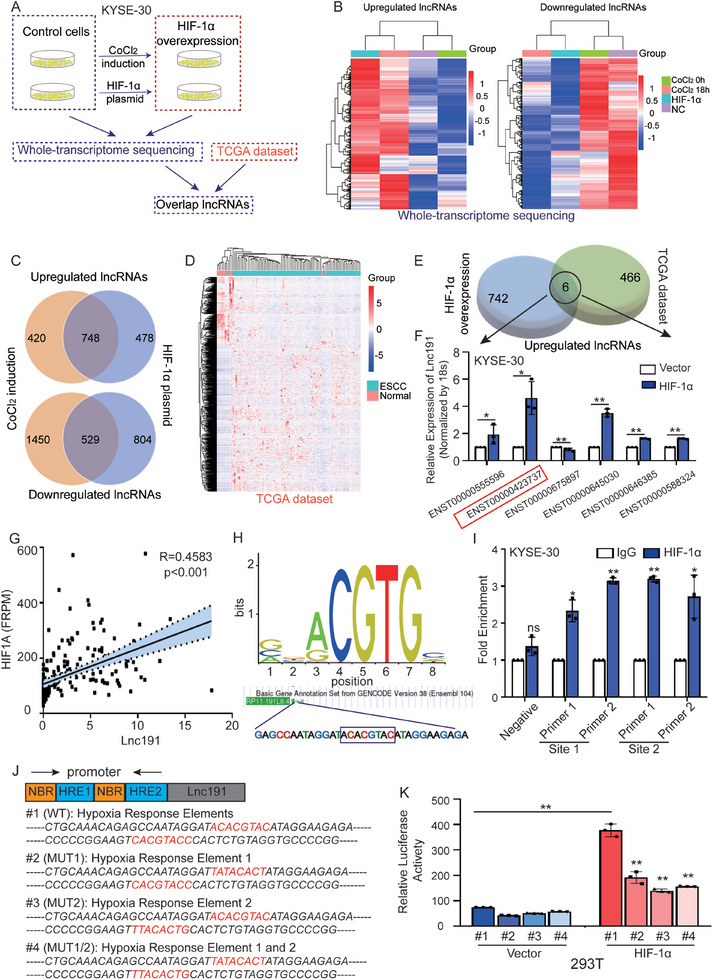
*Lnc191* is significantly upregulated under hypoxic conditions in ESCC. A) Schematic diagram describing the screening process of candidate lncRNAs. B) Hierarchical clustering analysis of significantly upregulated and downregulated lncRNAs in KYSE‐30 with or without HIF‐1α overexpression. C) Venn diagram of HIF‐1α downstream genes from two RNA‐seq analyses with CoCl_2_ induction or HIF‐1α plasmid. D) Hierarchical clustering analysis of differentially expressed lncRNAs in ESCC from TCGA dataset. E) Venn diagram of HIF‐1α downstream and TCGA dataset and 6 lncRNAs were selected. F) QPCR was performed to determine the relative RNA level of 6 lncRNAs in KYSE‐30 with HIF‐1α plasmid overexpression for 48 h. G) The correlation between *lnc191* and HIF1A RNA level in Cohort 2 was analyzed by Pearson correlation test. H) The JASPAR database indicated that HIF‐1α was a potential transcription factor of *lnc191* and the binding sequence was ACACGTAC. I) CHIP assays investigating the binding capacity of HIF‐1α to each HRE were conducted in KYSE‐30 cells. J) HREs in the *lnc191* promoter region were analyzed in JASPAR and the mutation site was described (#1: WT, #2: MUT1, #3: MUT2, #4: MUT1/2). NBR represents the non‐binding region. K) The relative luciferase reporter activity of HREs on the *lnc191* promoter in 293T cells. Student's unpaired *t*‐test (F, I, K) or Pearson correlation test (G) was used to calculate the *p*‐value. Three independent experiments were performed to obtain the mean ± SD value (n = 3 replicate experiments). *p < 0.05, **p < 0.01, ns: not significant.

In order to identify independent lncRNAs associated with ESCC progression under hypoxic conditions, a screening workflow was devised as depicted in **Figure** **1**A. Specifically, two models were employed to induce hypoxia: CoCl_2_ (cobalt chloride) induction^[^ ^18^ ^]^ or overexpression of HIF‐1α plasmid on KYSE‐30 cells. The whole‐transcriptome sequencing (Sinotech Genomics) analysis revealed that 1168 lncRNAs were upregulated and 1979 lncRNAs were downregulated in CoCl_2_‐induced hypoxia, while 1226 lncRNAs were upregulated and 1333 lncRNAs were downregulated in the HIF‐1α plasmid‐transfected cells (fold change > 1.5, Figure 1B). Subsequently, the ectopic expression of lncRNAs in the two models was overlapped. Accordingly, a total of 748 lncRNAs were found to be upregulated (Table S2, Supporting Information), while 529 were downregulated (Table S3, Supporting Information) under hypoxic conditions (Figure 1C). Next, to explore the specific hypoxia‐induced lncRNA that potentially functions in ESCC progression, we integrated these data with the ectopically expressed lncRNAs in the cohort of ESCC (fold change > 1.5, *p* < 0.05) from TCGA (Figure 1D; Table S4, Supporting Information), and 6 hypoxia‐induced lncRNAs that simultaneously highly expressed in ESCC tissues were identified finally (Figure 1E; Figure S1A, Supporting Information). As shown in Figure S1B (Supporting Information), online TF (transcriptional factor) prediction software JASPAR showed that multiple specific HIF‐1α binding sites were in the promoter regions of these 6 lncRNAs, and our results validated this prediction (Figure 1F). Among these lncRNAs, ENST00000423737, which is also known as RP11‐191L9.4 (*lnc191*) in the GENCODE database, exhibited the highest level of expression and aroused our attention.

We further verified the upregulation effect of hypoxic conditions on *lnc191* both using CoCl_2_ induction and overexpression of HIF‐1α in ESCC cells respectively (Figure S1C,D, Supporting Information). Moreover, the expression of HIF‐1α and *lnc191* was significantly and positively correlated in our Cohort 2 of ESCC (R = 0.4583, *p* < 0.001) (Figure 1G) and GEPIA data (R = 0.26, *p* < 0.001) (Figure S2A, Supporting Information). Furthermore, we identified two predicted HREs (0 to +400 nt relative to TSS) in the promoter regions of *lnc191* with high scores of 9.69 and 9.47 (Figure 1H; Figure S1B, Supporting Information). We then performed chromatin immunoprecipitation (ChIP) and luciferase assays to verify the binding sites of HIF‐1α to the *lnc191* promoter. Results of ChIP indicated that HIF‐1α was notably enriched at the two HREs of *lnc191* in all three ESCC cell lines (Figure 1I; Figure S2B, Supporting Information). To further confirm this result, we mutated the two HREs, respectively, and tested the luciferase value (Figure 1J). As expected, the luciferase activity was obviously increased after co‐transfection of HIF‐1α and *lnc191* WT promoter, but the luciferase activity in cells transfection of the mutant HREs showed significantly lower luciferase activity than the WT control (Figure 1K; Figure S2C, Supporting Information). Taken together, the data presented herein strongly support the notion that *lnc191*, as a new hypoxia‐responsible factor, is transcriptionally upregulated in ESCC directly.

### 2.2 Higher *Lnc191* Expression is Associated with Poorer Outcomes in ESCC Patients

**Figure 2:**
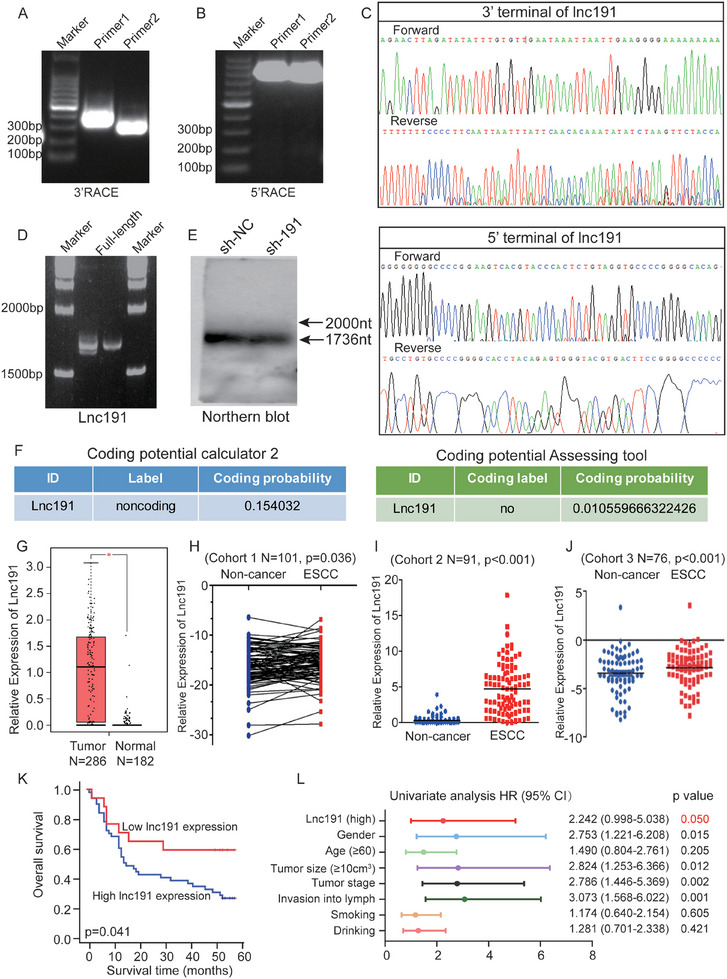
Higher *lnc191* expression is associated with poorer outcomes of ESCC patients. A) 3′ RACE assay of *lnc191*. B) 5′ RACE assay of *lnc191*. C) Sequencing showed the 3′ and 5′ terminals of *lnc191*. D) PCR assay showed the whole length of *lnc191*. E) Northern blot showed the whole length of *lnc191*. F) CPC2 and CPAT showed the coding probability of *lnc191*. G) Boxplot shows that the expression of *lnc191* was significantly increased in patients with esophageal cancer from GEPIA. H–J) Relative expression levels of *lnc191* in ESCC tissues compared with corresponding normal tissues in Cohort 1 (H, N = 101, p = 0.036), Cohort 2 (I, N = 91, *p* < 0.001) and Cohort 3 (J, N = 76, *p* < 0.001). K) Kaplan‒Meier survival curve of two groups of patients with ESCC from Cohort 2 (n = 67). L) Univariate Cox regression analyses of the survival of patients with ESCC (n = 67). Student's paired *t*‐test (H), Student's unpaired *t*‐test (I, J), Log‐rank test (K) or Univariate analysis (L) were used to obtain the *p‐value*. *p < 0.05.

Subsequently, the 3′ and 5′ rapid amplification of cDNA ends (RACE) assays were conducted to characterize the length of *lnc191*, which was first identified in our sequence data (**Figure** **2**A,B). Our results found that the 3′ end of lncRNA matched the reported sequence in the database, whereas the 5′ end shorter 79 nt than the sequence in the Ensembl database (ENST00000423737) and NCBI database (gene ID: 658310044) (Figure 2C). In addition, the full‐length PCR and northern blot assay also demonstrated that the full length of *lnc191*, which was 1736 nucleotides by sequence, not the 1815 nucleotides in ESCC cells (Figure 2D,E). Furthermore, the Coding Potential Calculator^[^ ^19^ ^]^ and Coding‐Potential Assessment Tool^[^ ^20^ ^]^ both predicted that *lnc191* lacked the ability to translate into a protein or polypeptide (Figure 2F). These results suggest that we identified a new lncRNA that has different length from the reported data.

We then elucidated whether *lnc191* has a critical role in ESCC progression. According to the Gene Expression Profiling Interactive Analysis (GEPIA) dataset, *lnc191* demonstrated marked elevation in esophageal cancer tissues (Figure 2G) and various other cancer types (Figure S2D, Supporting Information), suggesting that *lnc191* might be a pan‐cancer‐related gene. We then confirmed the heightened expression of *lnc191* in matched ESCC tissues in the 3 independent cohorts from Beijing and Shijiazhuang respectively (Cohort 1 from Beijing, N = 101; Cohort 2 was from Dec 2015 to Dec 2016 in Shijiazhuang, N = 91; Cohort 3 was from Mon 2017 to Dec 2018 in Shijiazhuang, N = 76) (Figure 2H–J). Furthermore, our study revealed positive correlations of the *lnc191* expression level with tumor size, clinical stage, lymph node metastasis, and overall survival rate in patients with ESCC (**Table** **1** and Figure 2K; Figure S2E, Supporting Information). Remarkably, Cox proportional hazards regression analysis demonstrated that *lnc191* expression was an independent prognostic factor for the overall survival of ESCC patients (*p* = 0.05, Figure 2L). Collectively, our mounting findings highlight that *lnc191* is a newly discovered hypoxia‐induced lncRNA that might play important roles in ESCC progression.

**Table 1:** The relationships of the expression of Lnc191 with the clinicopathological characteristics of the patients with esophageal carcinoma (n =73).

Clinicopathological feature	N	Esophageal carcinoma		χ2	*p* value
		low	high		
Gender				0.27	0.646
Male		12	43		
Female		5	13		
Age/year				0.297	0.586
<60		4	17		
≥60		13	39		
Clinical stage				4.012	0.045
I‐II		12	24		
III‐IV		5	32		
Tumor size(cm^3^)				6.541	0.038
≤5		3	8		
>5 and ≤10		7	8		
>10		7	40		
Invasion into lymph				3.921	0.048
Yes		6	35		
No		11	21		
Smoking				0.467	0.495
Yes		11	31		
No		6	25		
Drinking				0.094	0.76
Yes		9	32		
No		8	24		

### 2.3 Hypoxia‐Induced *Lnc191* Promotes ESCC Cells Proliferation and Metastasis in Vitro and in Vivo

**Figure 3:**
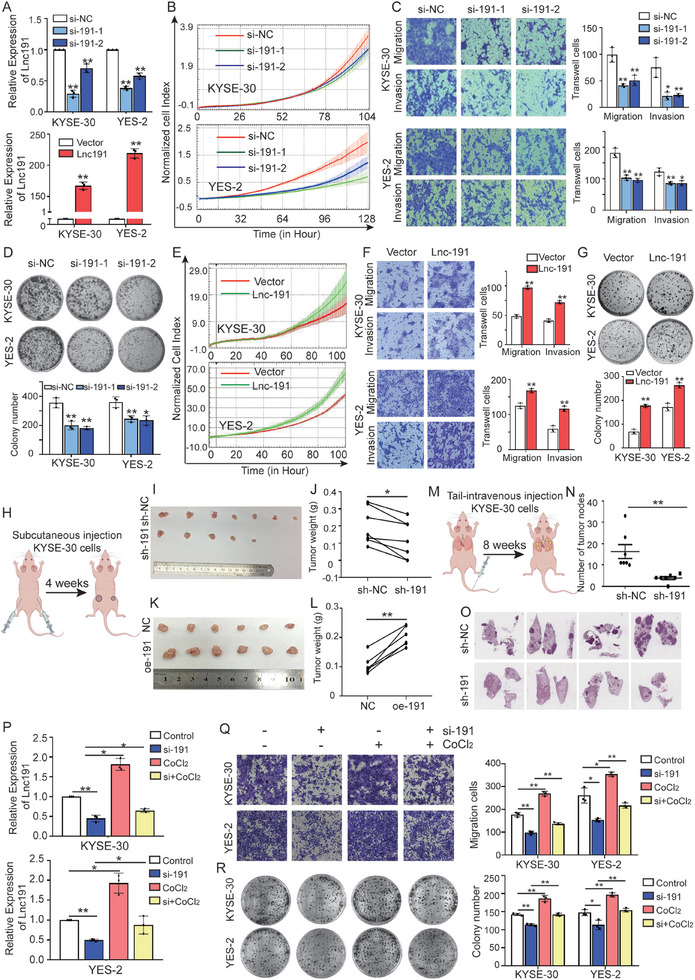
*Lnc191* promotes ESCC cell proliferation and metastasis in vitro and in vivo. A) QPCR analysis was performed to detect the RNA level of *lnc191* in KYSE‐30 and YES‐2 cells with *lnc191* siRNA or plasmid. B) The growth of KYSE‐30 and YES‐2 cells with or without *lnc191* knockdown was monitored by RTCA assays. C) Migration and invasion of KYSE‐30 and YES‐2 cells with or without *lnc191* knockdown were evaluated by transwell assays. D) Colony formation of KYSE‐30 and YES‐2 cells with or without *lnc191* knockdown were evaluated. E) The growth of KYSE‐30 and YES‐2 cells with or without *lnc191* plasmid was monitored by RTCA assays. F) Migration and invasion of KYSE‐30 and YES‐2 cells with or without *lnc191* plasmid. G) The effects of *lnc191* overexpression on the colony formation of KYSE‐30 and YES‐2 cells. H) Schematic diagram of subcutaneous injection model construction in nude mice. I, J) The representative images (I) and weight (J) of xenograft tumors of sh‐191 cells and controls. K, L) The representative images (K) and weight (L) of xenograft tumors of *lnc191* overexpressed cells and negative controls. M) Schematic diagram of mouse lung metastasis model construction in nude mice. N, O) HE staining of lungs from the tail vein injection model and statistic graph of the number of relative lung metastatic nodes using KYSE‐30 cells with *lnc191* knockdown. P) QPCR detected the relative expression of *lnc191* in KYSE‐30 and YES‐2 with *lnc191* knockdown or hypoxic condition. Q, R) Transwell (Q) and colony formation assays (R) were used to detect the migration and proliferation of KYSE‐30 and YES‐2 with *lnc191* knockdown or hypoxic conditions. Student's unpaired *t*‐test and Student's paired *t*‐test were used to calculate the *p‐value*. Three independent experiments were performed to obtain the mean ± SD value (n = 3 replicate experiments). *p < 0.05, **p < 0.01.

The elevated *lnc191* levels in clinical ESCC tissues prompted us to explore whether it is functionally important in ESCC cell growth and metastasis. To this end, we initially measured the *lnc191* expression in various ESCC cell lines and the immortalized normal esophageal epithelium cell lines NE2 and NE3. Notably, ESCC cells exhibited high expression of *lnc191*, while NE2 and NE3 cells displayed minimal to no expression (Figure S2F, Supporting Information). Then KYSE‐30 and YES‐2 were selected for subsequent experiments as both cell lines exhibited high expression levels of *lnc191* and were easy to transfect. Subsequently, two independent siRNAs and overexpression plasmid were designed to interrupt or enhance the *lnc191* expression in ESCC cell lines successfully (**Figure** **3**A). Accordingly, the knockdown of *lnc191* significantly decreased the proliferation rate of KYSE‐30 and YES‐2 (Figure 3B). Furthermore, *lnc191* was found to be required for the enhancement of migration and invasion of ESCC cells (Figure 3C), and further downregulated *lnc191* resulted in much fewer colonies in colony formation assay (Figure 3D). In contrast, *lnc191* overexpression markedly enhanced KYSE‐30 and YES‐2 cells’ proliferation, migration, invasion, and colony formation (Figure 3E–G), strengthening the oncogenic role of *lnc191*.

To further assess the role of *lnc191* in ESCC cells in an in vivo setting, we conducted subcutaneous injections of KYSE‐30 cells with either *lnc191* knockdown or overexpression, along with control cells, into the right or left flank of Balb/c nude mice, respectively (Figure 3H). After 4 weeks, tumors in the *lnc191*‐knockdown group weighed significantly less than those in the control group (Figure 3I,J). While tumors in the *lnc191*‐overexpression group were significantly heavier (Figure 3K,L). Additionally, we established tail vein injection models using KYSE‐30 cells to reveal the impact of *lnc191* on the metastasis of ESCC cells in vivo (Figure 3M). Upon sacrifice, the results of HE staining indicated a notably reduced number of lung metastatic nodes in the *lnc191* deficiency group (Figure 3N,O).

Due to the specific transcriptional activation of *lnc191* by HIF‐1α, we detected the role of hypoxia in *lnc191‐induced* proliferation and metastasis of ESCC cells. Firstly, the diminished levels of *lnc191* were restored under hypoxic conditions (Figure 3P). Consistently, functional results demonstrated that CoCl_2_‐induced hypoxia significantly enhanced the migration and colony formation abilities of ESCC cells, while these oncogenic properties were restored in *lnc191*‐silenced KYSE‐30 and YES‐2 cells (Figure 3Q,R). Taken together, these consistent findings provide evidence that hypoxia‐induced *lnc191* definitely promotes the malignant behaviors of ESCC cells both in vitro and in vivo.

### 2.4 *Lnc191* Directly Interacts with GRP78 in ESCC Cells

**Figure 4:**
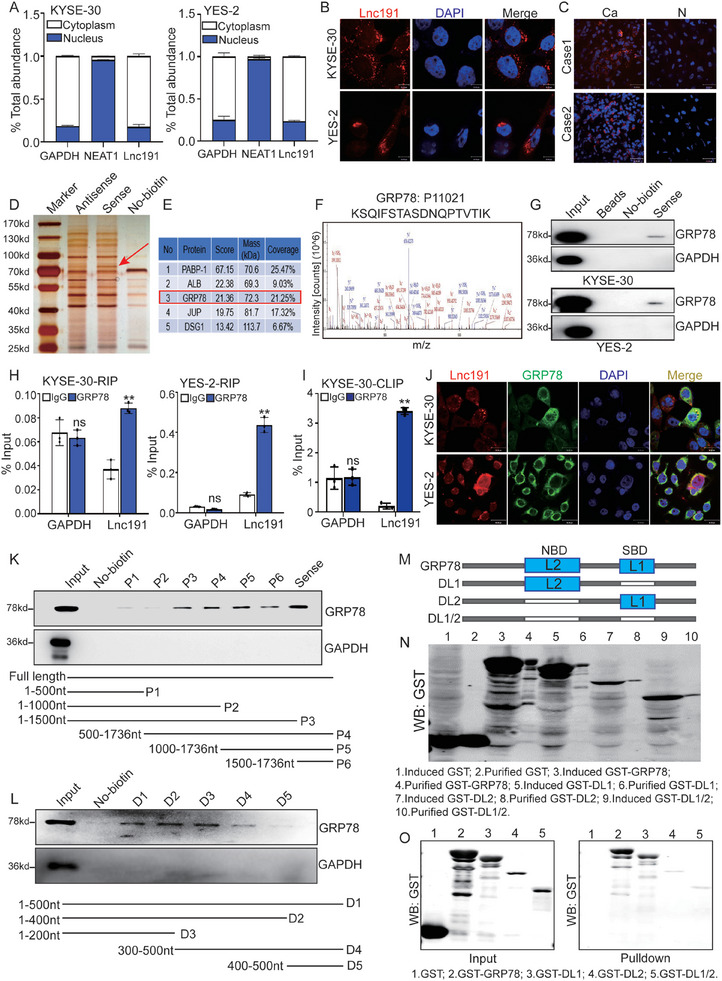
*Lnc191* directly interacts with GRP78 in ESCC cells. A) The expression level of *lnc191* in the subcellular fractions of KYSE‐30 and YES‐2 cells was detected by qPCR. Neat1 and GAPDH were used as nuclear and cytoplasmic markers, respectively. B, C) FISH assay was conducted to determine the subcellular location of *lnc191* in KYSE‐30 and YES‐2 cells (B) and ESCC tissues (C). D) Protein retrieved from the *lnc191* RNA pull‐down assays were analyzed by SDS‐PAGE with silver staining. E) The top proteins analyzed by MS were shown. F) Amino acid sequence of GRP78 as detected by MS. G) Western blotting of GRP78 in KYSE‐30 and YES‐2 protein retrieved from the *lnc191* RNA pull‐down assays. H) RIP assays were performed to confirm the association of GRP78 and *lnc191*. QPCR was used to detect the enrichment of *lnc191*. I) UV CLIP assay was performed to confirm the direct interaction between GRP78 and *lnc191*. J) The colocalization of *lnc191* (red) and GRP78 (green) was evaluated by confocal. K, L) Western blotting of GRP78 from RNA pull‐down assays with full‐length or truncated *lnc191* RNA probes. M) Schematic of deletion mutants of GRP78. L1 is the Substrate‐binding domain (SBD) and L2 is the Nucleotide‐binding domain (NBD). N) Western blot of purified deletion mutants of GRP78 with GST‐tag. O) Western blot of deletion mutants of GRP78 proteins retrieved by in vitro‐transcribed *lnc191*. Student's unpaired *t*‐test was used to calculate the *p‐value*. Three independent experiments were performed to obtain the mean ± SD value (n = 3 replicate experiments). **p < 0.01, ns: not significant.

The mechanism of lncRNAs is closely associated with their subcellular localization,^[^ ^21^ ^]^ thus we examined the subcellular localization of *lnc191* by performing cellular fraction followed RT‐qPCR analysis. Accordingly, *lnc191* was found to localize mainly in the cytoplasm of KYSE‐30 and YES‐2 cells (**Figure** **4**A), consistent with the results of FISH analysis both in ESCC cells and clinical samples (Figure 4B,C). Subsequently, we employed an RNA pulldown assay using in vitro transcribed biotin‐labeled RNAs, followed by mass spectrometric analyses, to explore the proteins that potentially interact with *lnc191*. Consequently, a distinct band within the size range of 70 kd to 100 kd exhibited specific enrichment in the *lnc191* pulldown (Figure 4D). Several potentially significant *lnc191*‐interacting proteins with high scores were documented in Figure 4E. Among the putative binding targets, PABP‐1 was detected both in the sense and antisense group, not a specific *lnc191* binding protein (Figure S2G). While GRP78, an endoplasmic reticulum chaperone protein was identified with a high abundance (coverage = 21.25%) among the potential interacting proteins of lncRNA (Figure 4F). Furthermore, the interaction between *lnc191* and GRP78 was validated in the RNA pull‐down samples obtained from two ESCC cell lines (Figure 4G). Consistently, PCR analysis confirmed the presence of *lnc191* in the RNA immunoprecipitation (RIP) products utilizing a GRP78 antibody (Figure 4H). Moreover, UV cross‐linking and immunoprecipitation (CLIP) revealed that *lnc191* directly interacts with GRP78 (Figure 4I; Figure S2H, Supporting Information). These results, in line with the data from immunofluorescence (IF) assays (Figure 4J), strongly suggest the co‐localization and interaction of *lnc191* and GRP78.

To further identify the specific binding fragment of *lnc191* that interacts with GRP78, a series of truncated mutants of *lnc191* were generated and subjected to RNA pulldown assay. The results indicated that a region spanning 1000–1500 nt of *lnc191* may be responsible for its interaction with GRP78 (Figure 4K). Subsequently, truncated mutants of this 500 nt region of *lnc191* were designed, revealing that the 1000–1200 nt region of *lnc191* serves as the specific binding site for GRP78 (Figure 4L). Moreover, previous research has identified two major functional domains of GRP78, namely the nucleotide‐binding domain (NBD) and substrate binding domain (SBD).^[^ ^22^ ^]^ In order to investigate the specific binding domain of GRP78 to *lnc191* in detail, a series of GRP78 fragments and GST vectors in the forms of GRP78 wild‐type or with one or two binding domains deleted were constructed (Figure 4M,N). Subsequent biotinylated *lnc191*‐pulldown assays confirmed that the NBD of GRP78 served as an independent domain for the physical interaction between GRP78 and *lnc191* (Figure 4O). Collectively, these findings provide compelling evidence that *lnc191* directly interacts with GRP78 protein on its nucleotide‐binding domain in ESCC cell lines.

### 2.5 Binding to GRP78 is Responsible for *Lnc191* to Promote ESCC Progression

**Figure 5:**
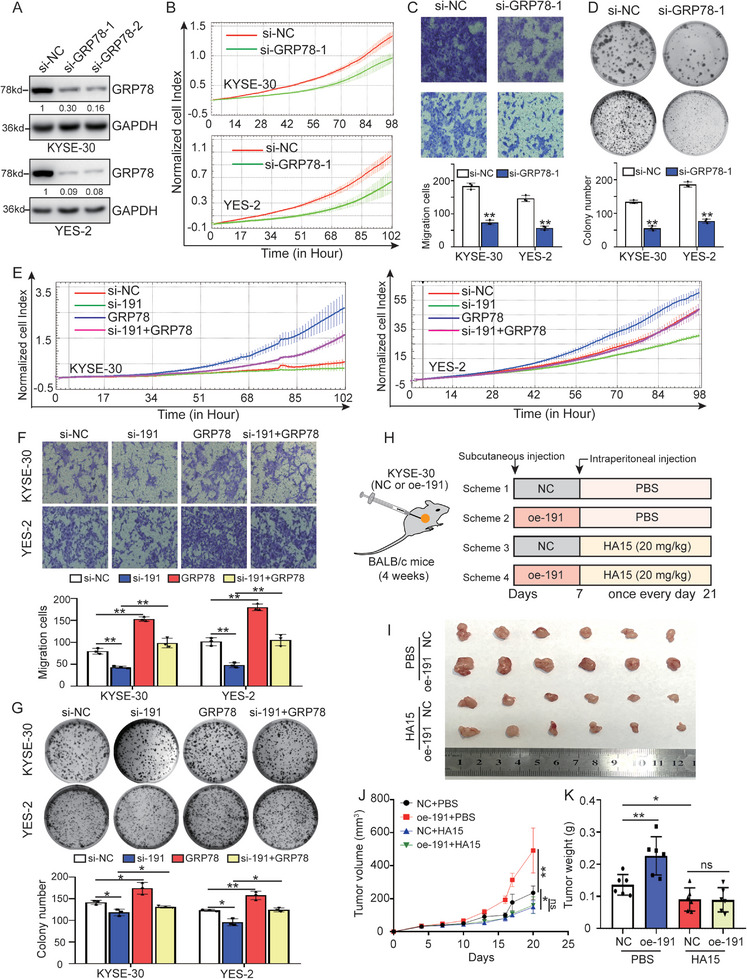
*Lnc191* promotes ESCC progression via binding to GRP78. A) Western blot detected the GRP78 in KYSE‐30 and YES‐2 with GRP78 knockdown. B) The growth of KYSE‐30 and YES‐2 cells with GRP78 knockdown was monitored by RTCA assays. C, D) Transwell (C) and colony formation assays (D) were used to detect the migration and proliferation of KYSE‐30 and YES‐2 with GRP78 knockdown. E) The growth of KYSE‐30 and YES‐2 cells with *lnc191* knockdown or GRP78 overexpression was monitored by RTCA assays. F, G) Transwell (F) and colony formation assays (G) were used to detect the migration and proliferation of KYSE‐30 and YES‐2 with *lnc191* knockdown or GRP78 overexpression. H) Schematic presentation of the experimental protocol. I) Representative pictures of tumors. n = 6 per group. J, K) Tumor volume (J) and weight (K) of mice from each group. Student's unpaired *t*‐test was used to calculate the *p‐value*. Three independent experiments were performed to obtain the mean ± SD value (n = 3 replicate experiments). *p < 0.05, **p < 0.01.

GRP78 has been implicated in tumor progression across various cancers and is closely correlated with unfavorable prognosis of patients with glioma, lung cancer, and head and neck cancer (Figure S3A,B, Supporting Information). Additionally, our team has clarified the GRP78 signaling pathway during ER stress‐induced EMT in ESCC.^[^ ^23^ ^]^ In this study, our results confirmed the upregulation of GRP78 in Cohort 2 of ESCC samples (Figure S3C, Supporting Information). To further ascertain whether GRP78 truly functions as a target of *lnc191* in ESCC, we explored the role of GRP78 in the malignant phenotypes of ESCC cells. Firstly, the knockdown efficiency was initially confirmed (**Figure** **5**A), and the significant suppression of cell proliferation, colony formation, and migration was observed upon knockdown of GRP78 (Figure 5B–D), indicating the crucial role of GRP78 in ESCC development.

To further investigate the impact of GRP78 on *lnc191*‐induced ESCC progression, the cell growth rate, migration, and colony formation ability were assessed in ESCC cells manipulated for both *lnc191* and GRP78. As depicted in Figure 5E, the overexpression of GRP78 partially overcomes the decreased cell proliferation induced by *lnc191* knockdown in ESCC cells. Consistently, supplementation of GRP78 abrogated the diminished migration and colony formation in *lnc191* knockdown ESCC cells (Figure 5F,G). In addition, to elucidate how GRP78 binding influences ESCC progression via *lnc191* in vivo, we developed animal models utilizing KYSE‐30 cells with stable overexpression of *lnc191* and the GRP78 inhibitor HA15 (Figure 5H). The results indicated that *lnc191* overexpression led to an increase in tumor volume and weight of ESCC, while inhibition of GRP78 abrogated this effect (Figure 5I–K). These findings underscore the critical involvement of GRP78 in the *lnc191*‐mediated enhancement of proliferation and migration in ESCC cells.

### 2.6 *Lnc191* Regulates ERK/MAPK Signaling Pathway Through Interacting with GRP78 in ESCC

**Figure 6:**
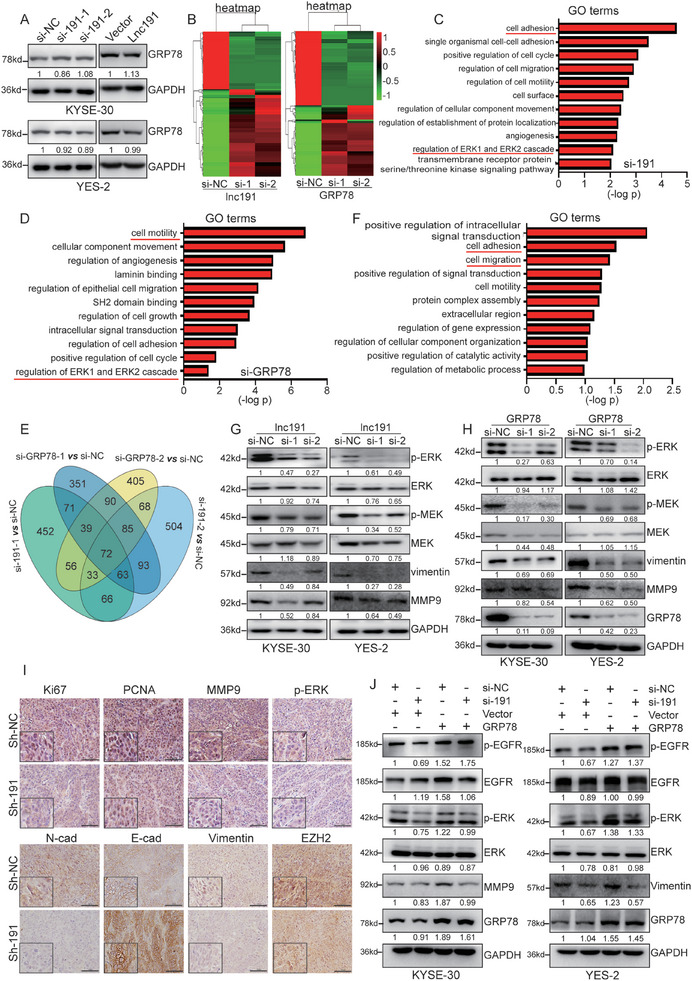
*Lnc191* and GRP78 regulate the ERK/MAPK signaling pathway in ESCC cell lines. A) Western blot detected the GRP78 in KYSE‐30 and YES‐2 with *lnc191* knockdown or overexpression. B) Heatmap of DEGs by RNA‐seq in KYSE‐30 cells with *lnc191* or GRP78 knockdown. C, D) The significantly enriched GO terms for DEGs from RNA‐seq in *lnc191*‐knockdown (C) and GRP78‐knockdown (D) KYSE‐30 cells. E) Venn diagram showing the downstream target genes regulated by *lnc191* and GRP78. F) The significantly enriched GO terms for 72 DEGs in *lnc191* and GRP78 knockdown. G, H) Western blot showing the expression level of p‐ERK and p‐MEK in KYSE‐30 and YES‐2 with *lnc191* (G) or GRP78 knockdown (H). I) Immunohistochemical staining of Ki67, PCNA, MMP9, p‐ERK, N‐cad, E‐cad, Vimentin, and EZH2 in xenograft tumors of sh‐191 cells and controls, respectively. J) Western blot detecting the p‐EGFR, p‐ERK, MMP9, and Vimentin in KYSE‐30 and YES‐2 with *lnc191* knockdown or GRP78 overexpression.

We then proceeded to explore the molecular mechanism and outcomes of this interaction. Intriguingly, *lnc191* had no influence on the protein expression level of GRP78 (**Figure** **6**A). Subsequently, we identified the specific signaling pathway responsible for the progression of ESCC mediated by *lnc191* and GRP78 respectively. Firstly, RNA sequencing and subsequent pathway enrichment analyses were conducted to compare control cells with *lnc191*‐ or GRP78‐silenced cells in KYSE‐30 and get the changed genes in these two data sets (Figure 6B). Gene ontology (GO) and GSEA analysis revealed that the affected genes in *lnc191* knockdown cells and GRP78‐silenced cells were predominantly enriched in signaling pathways related to cell migration, cell adhesion, cell cycle, and ERK1/ERK2 (Figure 6C,D; Figure S3D, Supporting Information). KEGG analysis revealed that MAPK signaling pathway is significantly overlapped in the top 30 enriched pathways of these two data sets (Figure S3E). We further examined the intersection of these two groups of data, and identified a total of 72 genes that overlapped between the *lnc191*‐ and GRP78‐silenced cells, also exhibiting enrichment in cell adhesion, cell motility, and cell migration (Figure 6E,F), supporting the hypothesis that *lnc191* and GRP78 might orchestrate the common downstream target genes.

Notably, the ERK1 and ERK2 cascade and MAPK signaling pathway were found to be significantly altered in *lnc191*‐silenced ESCC cells, which is consistent with the previous reports that GRP78 is overexpressed in cancer cells to contribute to activating the ERK1/2 signaling pathway.^[^ ^24^, ^25^ ^]^ Accordingly, we validated the impact of *lnc191* and GRP78 on the ERK/MAPK signaling pathway. Our findings showed a notable reduction in p‐ERK, p‐MEK, and downstream targets vimentin and MMP9 expression following the knockdown of *lnc191* and GRP78 in ESCC cells (Figure 6G,H). In addition, immunohistochemical analysis demonstrated that proliferative and migratory biomarkers Ki67, PCNA, MMP9, N‐cad, Vimentin, and EZH2 were reduced, while E‐cad expression was elevated in tumor tissues from *lnc191*‐knockdown xenograft mice, and the expression of p‐ERK was also reduced, suggesting that knockdown of *lnc191* suppressed ESCC proliferation, metastasis and p‐ERK pathway in vivo (Figure 6I; Figure S4A, Supporting Information). Moreover, the enhanced expression of GRP78 partially mitigated the decrease in p‐ERK and upstream p‐EGFR induced by *lnc191* knockdown (Figure 6J). Collectively, these results provide evidence that both *lnc191* and GRP78 play vital roles in promoting the development and progression of ESCC by regulating the GRP78/p‐ERK signaling axis.

### 2.7 *Lnc191* Facilitates the Membrane Translocation of GRP78 and the Formation of GRP78/EGFR Complex

**Figure 7:**
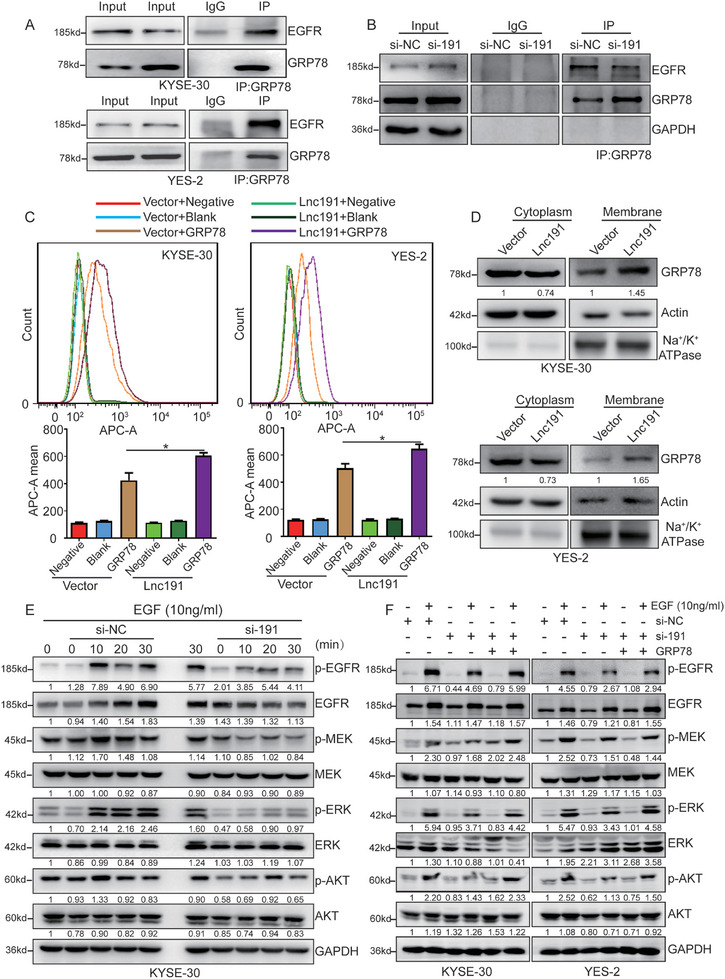
*Lnc191* facilitates the membrane translocation of GRP78 and the formation of GRP78/EGFR complex. A) The interaction between GRP78 and EGFR was evaluated using IP assays. B) IP and western blot analysis for GRP78 and EGFR in KYSE‐30 with *lnc191* knockdown. C) The abundance of GRP78 on the membrane of ESCC cells transfected with the indicated plasmids was reflected by flow cytometry. D) The subcellular localization of GRP78 in KYSE‐30 and YES‐2 with *lnc191* overexpression was analyzed by western blot. Actin and Na^+^/K^+^ ATPase were used as cytoplasmic and membrane markers, respectively. E) KYSE‐30 cells transfected with either control siRNA or *lnc191* siRNA were stimulated with 10 ng mL^−1^ of EGF for the indicated duration. F) KYSE‐30 or YES‐2 cells transfected with either *lnc191* siRNA or GRP78 plasmid were stimulated with 10 ng mL^−1^ of EGF for the indicated duration. Cell lysates were analyzed by western blotting. Student's unpaired *t*‐test was used to calculate the *p*‐value. Three independent experiments were performed to obtain the mean ± SD value (n = 3 replicate experiments). *p < 0.05.

Subsequently, we elucidated the detailed mechanism by which *lnc191* regulated the ERK/MAPK pathway through interacting with GRP78. Interestingly, our results indicated that GRP78 could not directly interact with ERK or MEK but rather interacts directly with EGFR (**Figure** **7**A; Figure S4B, Supporting Information), a transmembrane tyrosine kinase receptor, which is extensively studied for its role in controlling cell proliferation, differentiation, migration, and other processes through truncation or ligand binding.^[^ ^26^, ^27^ ^]^ Consistently, our results further demonstrated that the deletion of *lnc191* impaired the binding between GRP78 and EGFR (Figure 7B). Conversely, GRP78 is a cytoplasmic and membranal distribution protein, which promotes us to hypothesize that GRP78 might translocate to the cell membrane in order to bind with EGFR, and its interaction with *lnc191* might take part in this procession. To test the hypothesis, *lnc191* overexpressed ESCC cells were treated with an anti‐GRP78 antibody to the detect of GRP78 expression on the cell surface. The results demonstrated a significant increase in fluorescence intensity in cells overexpressing *lnc191*, suggesting that *lnc191* overexpression facilitated GRP78 translocation to the membrane (Figure 7C). To confirm this notion, the protein in the membrane and cytoplasm was separately extracted, and an increased localization of GRP78 on the cell membrane was investigated in the cell's overexpression of *lnc191*, accompanied by a decrease in the level of GRP78 in the cytoplasm (Figure 7D), thus our results provided definite data that *lnc191* enhanced the translocation of GRP78 to the cell membrane.

To further investigate the impact of *lnc191*‐mediated GRP78 relocation on the activation of EGFR, the reported phosphorylation sites^[^ ^28^ ^]^ that influenced the activity of EGFR were examined. The obtained data revealed a significant decrease in EGFR Tyr845 in *lnc191*‐silenced ESCC cells, while the site of Tyr992, Tyr1148, Tyr1045, Thr669, and Ser1046/1047 showed modest phosphorylation change (Figure S4C, Supporting Information). Furthermore, in order to assess the activation status of EGFR and key downstream signal pathways (such as MEK, ERK, and AKT), cells in which *lnc191* was knocked down or not were treated with exogenous soluble EGF. The findings revealed significant impairment in the activation of p‐EGFR (Tyr845), p‐MEK, p‐ERK, and p‐AKT in cells lacking *lnc191* when stimulated with EGF (Figure 7E; Figure S4D, Supporting Information). Moreover, the impairment of these signaling pathways was significantly reversed by overexpression of GRP78 (Figure 7F), suggesting that the *lnc191*‐GRP78 axis plays a crucial role in EGFR activity and the functioning of downstream proteins. In conclusion, these results collectively validate that *lnc191* directly interacts with GRP78, leading to the trans‐localization of GRP78 to the cell membrane, where it binds with EGFR and promotes its phosphorylation on the Tyr845 site.

### 2.8 The HIF‐1α/*Lnc191*/GRP78/p‐ERK Axis is Involved in ESCC Development

**Figure 8:**
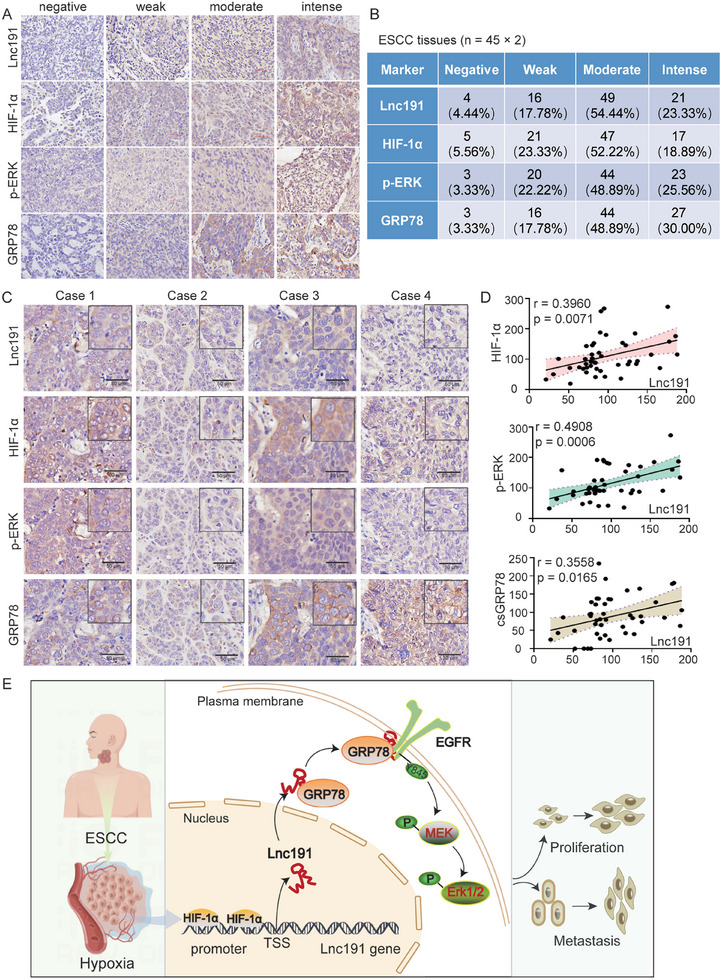
The HIF‐1α/*Lnc191*/p‐ERK axis is involved in ESCC development. A) The representative expression intense of *lnc191*, HIF‐1α, p‐ERK, and GRP78 with IHC. B) The number of cases with different staining intensities of HIF‐1α, *lnc191*, GRP78, and p‐ERK. C) The representative images of *lnc191*, HIF‐1α, p‐ERK, and GRP78 in ESCC tissue chips with the same location. D) The correlation between the expression of *lnc191* and HIF‐1α, p‐ERK, and csGRP78 using IHC assays. E) Model of HIF‐1α/*Lnc191*/GRP78/p‐ERK axis regulating ESCC progression. The Pearson correlation test was used to calculate the *p*‐value.

In order to delve deeper into the role of the HIF‐1α/*lnc191*/GRP78/p‐ERK axis in the context of clinical samples of ESCC, an examination of their expression level was performed on ESCC tissue chip (Figure S5A–D, Supporting Information). The staining intensity of HIF‐1α, *lnc191*, GRP78, and p‐ERK was categorized as negative, weak, moderate, or intense, and the representative stained tissue slides from tumors of various grades are presented in **Figure** **8**A. Then we counted the proportion of tissues with different staining intensities (Figure 8B). Our findings indicated that tissues with high *lnc191* expression also exhibited elevated levels of HIF‐1α, p‐ERK, and cell surface GRP78 (csGRP78) (Figure 8C). Furthermore, correlation analyses revealed a positive association between *lnc191* levels and the protein level of HIF‐1α (r = 0.3960, p = 0.0071), p‐ERK (r = 0.4908, p = 0.0006) and csGRP78 (r = 0.3558, p = 0.0165) (Figure 8D). In conclusion, it is postulated that the dysregulation of the HIF‐1α/*Lnc191*/p‐ERK axis plays a significant role in ESCC development.

In summary, our findings have revealed that under hypoxic conditions, HIF‐1α transcriptionally activates *lnc191*, which in turn facilitates the progression of ESCC by directly binding to GRP78. Subsequently, *lnc191* expedites the translocation of GRP78 to the cell membrane, where it forms a complex with EGFR, ultimately leading to the Tyr845 phosphorylation of EGFR and activation of the ERK/MAPK pathway (Figure 8E). These discoveries have illuminated novel molecular mechanisms underlying ESCC progression mediated by hypoxic and EGFR pathways, thereby offering valuable insights for the development of innovative therapeutic targets and prognostic markers.

## 3 Discussion

Hypoxia is a critical orchestra of the tumor microenvironment to regulate tumorigenesis.^[^ ^29^ ^]^ HIF‐1α activation plays a crucial role in cancer processes like proliferation, metastasis, invasion, and angiogenesis via various signaling pathways.^[^ ^30^, ^31^ ^]^ However, the precise mechanism underlying hypoxia is still unclear. Recent studies show that hypoxia regulates lncRNAs by binding HIF‐1α to HREs in promoters, influencing their transcription. In the present study, we identified *lnc191* as a hypoxia‐inducible lncRNA. *Lnc191* expression was increasing under low oxygen conditions or by overexpressing HIF‐1α. Two potential HREs in the *lnc191* promoter were identified using the JASPAR database and verified they were the HIF‐1α binding sites.

In recent years, lncRNAs, which exhibit aberrant expression across numerous tumor types, have been identified as playing pivotal roles in the pathogenesis of various malignancies, including lung,^[^ ^32^ ^]^ prostate,^[^ ^33^ ^]^ breast,^[^ ^34^ ^]^ gastric^[^ ^35^ ^]^ and pancreatic cancers.^[^ ^36^ ^]^ In the study, we identified that *lnc191* was upregulated in ESCC tissues and cell lines. Subsequent correlation analysis demonstrated that elevated *lnc191* expression is significantly associated with poor patient prognosis. In addition, functional studies revealed that the upregulation of *lnc191* enhanced ESCC cell proliferation and metastasis both in vitro and in vivo. Overall, the findings suggest that *lnc191* functions as an oncogene and presents a potential therapeutic target for ESCC. Interestingly, in the process of our research, lncRNA EPIC1 (567 nt), a different variant with *lnc191*, was reported to be upregulated in several cancers, including breast cancer and lung cancer, by interacting with MYC.^[^ ^37^, ^38^, ^39^ ^]^ In our study, we identified a new variant of EPIC1 in ESCC named *lnc191* (1736 nt), which was determined through RACE and northern blot techniques. This finding represents the first report of *lnc191*’s involvement in the progression of ESCC and suggests different variants play important roles in various cancers.

Additionally, we successfully identified GRP78 as a direct binding protein of *lnc191*. GRP78, also named BIP or HSP5A, is a multifunctional protein with diverse activities beyond its well‐established role in the unfolded protein under ER stress.^[^ ^40^ ^]^ As an oncogene, GRP78 is critically involved in the survival, proliferation, and development of chemoresistance in tumor cells across a range of cancers, including ovarian cancer,^[^ ^41^ ^]^ breast cancer,^[^ ^42^ ^]^ prostate cancer,^[^ ^43^ ^]^ and colorectal cancer.^[^ ^44^ ^]^ In the study, we revealed that binding to GRP78 is responsible for *lnc191* to promote ESCC progression in vitro and in *vivo*. GRP78/BIP is a prominent chaperone found in the endoplasmic reticulum (ER) of normal tissues. However, in cancerous cells, GRP78 is also observed in various cellular compartments, including the mitochondria, cytosol, nucleus, and cell surface.^[^ ^45^ ^]^ Notably, GRP78 is abnormally localized on the surface of numerous cancer cells, such as those found in the lung, breast, colon, AML, and liver cancers, while its expression is rare in normal cells.^[^ ^46^, ^47^, ^48^, ^49^ ^]^ This aberrant localization offers a promising avenue for tumor‐specific therapeutic strategies and targeted drug delivery, minimizing damage to healthy tissues. Moreover, accumulating evidence suggests that cell surface GRP78 (csGRP78) enhances cancer aggressiveness and has been identified as a potential target or anticancer intervention.^[^ ^46^, ^50^ ^]^ As a receptor involved in cell surface signaling, csGRP78 can be activated by multiple ligands, leading to the activation of various downstream signaling pathways that regulate cancer cell proliferation, survival, and apoptosis.^[^ ^51^ ^]^ In this study, we observed that *lnc191* interacts with GRP78 and facilitates its translocation to the cell membrane. Furthermore, we demonstrated that *lnc191* enhances the binding of GRP78 with EGFR. These newly identified functions of GRP78 are contingent upon its subcellular localization.

Furthermore, our findings revealed a unique mechanism that *lnc191* accelerated the phosphorylation of EGFR by regulating the GRP78 translocation to the cell surface and its subsequent binding to EGFR. This discovery broadens our comprehension of the mechanisms by which lncRNA molecules influence EGFR phosphorylation. The overexpression and amplification of EGFR are consistently observed in ESCC and are correlated with tumor progression and unfavorable prognoses.^[^ ^52^, ^53^ ^]^ Aberrant activation of EGFR is implicated in various cellular processes, including reduced apoptosis, cell cycle redistribution, increased DNA repair and hypoxia.^[^ ^54^ ^]^ Recent evidence suggests that phosphorylation at Y845 of EGFR is prevalent in several types of cancer cells, and associated with cell proliferation, high malignancy, and drug resistance.^[^ ^55^, ^56^ ^]^ These reports demonstrate that under specific conditions, the Y845 phosphorylation of EGFR is essential for EGFR‐dependent malignancy in cancer cells.

Our analysis of gene expression profiles in ESCC cells provides evidence suggesting that *lnc191* potentially modulates the ERK1/2 and MAPK signaling pathways. The activation of the ERK pathway by GRP78 is likely to contribute to tumor cell proliferation and metastasis. Cell‐surface GRP78 has the ability to bind to Src, and multiple lines of evidence suggest that Src protein can activate the MAPK pathway. As a receptor present on the surface of tumor cells, csGRP78 can interact with various signaling molecules, leading to the initiation of downstream cascades involving STAT3, RAS/MAPK and PI3K/AKT/mTOR. This ultimately promotes cellular proliferation and survival.^[^ ^57^, ^58^ ^]^ Given the wide range of cellular processes regulated by MAPK, including cell metabolism, proliferation, motility, apoptosis, survival, and differentiation, it is not surprising that *lnc191* can activate the GRP78/EGFR/ERK pathway in ESCC cells, thereby promoting tumor progression. Therefore, it is speculated that *lnc191* may be an important marker for predicting the efficacy of EGFR antibody drugs in the treatment of ESCC.

In summary, we have identified a lncRNA called *lnc191* and elucidated the regulatory mechanisms of *lnc191* induced by hypoxia in promoting the progression of ESCC. Specifically, *lnc191* interacts with GRP78 and facilitates the phosphorylation activity of EGFR at the Y845 site, thereby activating the p‐EGFR/p‐ERK signaling pathway and accelerating ESCC progression. These findings provide insights into the mechanisms underlying hypoxia‐induced ESCC progression through the regulation of the GRP78/EGFR/p‐ERK axis. Moreover, these findings indicate that *lnc191* could serve as a potential biomarker for EGFR antibody therapeutic strategy and as the diagnosis of treatment with ESCC.

## 4 Experimental Section

### Clinical Specimens

The ESCC tissues and the matched adjacent non‐neoplastic tissues were collected at the Fourth Hospital of Hebei Medical University (Cohort 2 was from Dec 2015 to Dec 2016, Cohort 3 was from Mon 2017 to Dec 2018) and Chinese PLA General Hospital (Cohort 1). Informed consent for the utilization of samples was secured from all patients, and ethical approval was granted by the ethics committee of the Fourth Hospital of Hebei Medical University (Approval No. 2017MEC112). Each sample was diagnosed by a team of 2–3 experienced pathologists. The inclusion criteria specified that patients must have primary ESCC and must have undergone surgery as the initial treatment modality.

### Cell Culture

The human ESCC cell lines utilized in this study were generously provided by Dr. Yutaka Shimada from Kyoto University and were maintained in RPMI‐1640 (Gibico, USA) supplemented with 10% FBS (BI, Israel). Immortalized esophageal epithelium cell lines NE2 and NE3 were kindly supplied by Prof. Enmin Li from Shantou University and maintained in the EpiLife/dKSFM (1:1) medium (Thermo, USA). All cell lines underwent authentication through short tandem repeat (STR) profiling.

### TCGA Analysis

To investigate gene expression within the ESCC cohort from TCGA, RNA sequence fragments were acquired per kilobase million (FPKM) values and raw read counts of the ESCA cohort from the TCGA GDC portal. The dataset encompasses a total of 173 samples, comprising 161 primary tumor samples (81 ESCCs and 80 EACs), 1 metastatic ESCC sample, and 11 adjacent normal tissue samples. For the purpose of gene expression analysis, 81 primary ESCC samples were utilized alongside 11 adjacent normal tissue samples.

### RNAseq Analysis

LncRNA sequencing analysis was performed at Sinotech Genomics (Shanghai, China), and the files were deposited in the Sequence Read Archive (SRA) database under accession PRJNA1124251. The mRNA sequencing of KYSE‐30 knockdown of *lnc191* or GRP78 was performed at CapitalBio Technology (Beijing, China), and the files were deposited in the SRA database under accession PRJNA1123329. Total RNA was extracted using TRIzol (Invitrogen, Carlsbad, CA, USA). The differentially expressed genes were screened according to fold change ≥ 1.5.

### Antibodies, siRNA, Plasmids and Primers

The following antibodies were used in this study: the anti‐HIF‐1α (Cat# 36169), anti‐GST (Cat# 2624), anti‐p‐ERK (Cat# 4370), anti‐ERK (Cat# 4695), anti‐p‐MEK (Cat# 9121), anti‐MEK (Cat# 9122), anti‐MMP9 (Cat# 13667), anti‐p‐EGFR(Y845) (Cat# 6963), anti‐p‐EGFR(Y992) (Cat# 2235), anti‐p‐EGFR(Y1148) (Cat# 4404), anti‐p‐EGFR(Y1045) (Cat# 2237), anti‐p‐EGFR(T669) (Cat# 3056), anti‐EGFR (Cat# 4267), anti‐Na, K‐ATPase α1 (Cat# 23565), anti‐p‐AKT (Cat# 4060), anti‐AKT (Cat# 4691) were purchased from Cell Signaling Technology (Massachusetts, USA); anti‐GRP78 (Cat# 11587‐1‐AP), anti‐actin (Cat# 66009‐1‐Ig), anti‐GAPDH (Cat# 60004‐1‐Ig), anti‐Vimentin (Cat# 10366‐1‐AP), were purchased from Proteintech (Wuhan, China).

The siRNAs of *lnc191* and GRP78 were synthesized by GenePharma (Suzhou, China). *Lnc191* and GRP78 plasmids were synthesized by Genenary (Shanghai, China). The sequences of siRNAs and primers of qPCR, RACE and CHIP were provided in Table S1 (Supporting Information).

### Cell Proliferation and Transwell Assays

The experimental procedures were conducted as outlined in prior studies.^[^ ^59^ ^]^ In summary, cell proliferation assays were executed using the xCELLigence Real‐Time Cell Analyser (RTCA)‐MP system (Acea Biosciences/Roche, Switzerland), with automated cell detection occurring at 15 min intervals. The ESCC cells were seeded in the upper chamber, and cells that migrated or invaded were subsequently stained with crystal violet and quantified.

### Fluorescence in Situ Hybridization (FISH)

The fluorescent probe for *lnc191* was customized in Biosearch. Cell or tissue samples were fixed with 4% paraformaldehyde and permeabilized using 0.5% Triton X‐100. The samples were then incubated overnight at 37°C with a 1% probe solution in an SSC buffer. Subsequently, nuclear staining was performed using DAPI. Imaging was conducted using a confocal laser scanning microscope (Leica, Germany).

### Chromatin Immunoprecipitation (CHIP) Assay

The procedure was conducted in accordance with the guidelines provided by the CHIP analysis kit (ThermoFisher, Shanghai, China). ESCC cells were cross‐linked with formaldehyde for 10 min at room temperature, and the reaction was quenched using a glycine buffer. The harvested cells underwent sonication to obtain chromatin fragments of 200–500 bp, and the fragments were incubated with HIF‐1α or control antibody at 4°C overnight. Then magnetic beads were introduced to the complex and incubated for an additional 3h. Finally, the beads were subjected to a series of washes, and Proteinase K was added to facilitate the purification of DNA using a column. QPCR was then performed to assess the enrichment at the HIF‐1α promoter.

### Dual‐Luciferase Reporter Assay

293T and YES‐2 cells were cultured in 12‐well plates. Upon reaching a confluence of 60–80%, the cells were co‐transfected with either the HIF‐1α plasmid or a vector control, alongside the *lnc191* promoter or its mutants (MUT1, MUT2 or MUT1/2) for a duration of 48h. Subsequently, luciferase activity was quantified using the Dual Luciferase Reporter Assay System kit (Promega) and normalized to *R. reniformis* luciferase activity.

### RNA Pulldown Assay

Full‐length sense and antisense *lnc191* or its fragments were linearized using the appropriate restriction enzymes BamHI or EcoRI. Subsequently, in vitro transcription was performed utilizing the MEGAscript T7 Transcription Kit (Life Technology) and Bio‐16‐UTP (Life Technology), following the manufacturer's protocol. For the RNA pulldown assay, the labeled probes were denatured at 95°C for 2 min and immediately placed on ice. Streptomycin beads were blocked with tRNA and 20% BSA, while cell lysates were pre‐cleared using streptomycin beads. The probes, cell lysates and beads were mixed together, washed with wash buffer containing 100 mm NaCl 6 times, and got proteins that could bind probes eventually.

### RNA Immunoprecipitation (RIP)

RIP assays were conducted utilizing the Magna RIP RNA‐Binding Protein Immunoprecipitation Kit (Merck Millipore) in accordance with the manufacturer's protocol. Subsequently, the coprecipitated RNAs were quantified using quantitative RT‐PCR.

### Cross‐Linking and Immunoprecipitation (CLIP)

The CLIP assay was performed according to the previously reported methods.^[^ ^60^ ^]^ Briefly, KYSE‐30 cells were cross‐linked at 254 nm wavelength with 400 mJ cm^−2^ of UVB and lysed using 500 µL ice‐cold Buffer A. Then IgG and GRP78 antibodies were separately incubated with the lysis. Protein G Sepharose beads were added to each mixture. Finally, the obtained protein G beads were Sequentially washed using Low‐salt buffer, High‐salt buffer, LiCl buffer, and TE buffer (pH 8.0) and subjected to Trizol. Then the coprecipitated RNAs were quantified through q‐PCR.

### GST Pull Down

Recombinant GST‐tagged GRP78 plasmids (WT, D1, D2, and D1/2) were constructed (Generay, Shanghai, China) and transformed into BL21 (DE3) (TransGen Biotech). Corresponding proteins were purified using Pierce GST Spin Purification Kit (ThermoFisher Scientific) in accordance with the manufacturer's protocol, and incubated with biotinylated *lnc191*, then pulled down by streptomycin beads. The bound protein was eluted by boiling in the protein loading buffer and subjected to a western blot using primary anti‐GST antibody.

### Separation of Biotin‐Labeled Membrane Protein from Plasma Protein

Excess EZ‐Link Sulfo‐NHS‐SS‐Biotin (ThermoFisher Scientific) was dissolved in the PBS, and then incubated with ESCC cells for 30 min at room temperature. 1% NP‐40 containing protease inhibitors was used to lyse the cells. Next, cell lysate and streptavidin beads were incubated together overnight at 4°C. The supernatant was discarded after centrifugation, and then western blot analysis was performed.

### Immunohistochemistry (IHC)

The IHC of clinical tissues was performed on a tissue chip (OD‐CT‐DgEso04‐004, OUTDO BIOTECH Co., Ltd, Shanghai), which contained 45 ESCC patients. The proportion of positively stained tumor cells per slide, expressed as a percentage (ranging from 0% to 100%), was multiplied by the predominant staining intensity pattern, categorized as follows: 0 for negative, 1 for weak, 2 for moderate, and 3 for intense. Consequently, the composite score could vary from 0 to 300.

### Animal Study

Male BALB/c nude mice (aged 4–5 weeks) were purchased from Beijing HFK Bioscience (Beijing, China). The mice were housed in the Laboratory Animal Center at the Fourth Hospital of Hebei Medical University under pathogen‐free conditions, following the protocol approved by the Animal Ethics Committee of the Fourth Hospital of Hebei Medical University (IACUC‐4th Hos Hebmu‐2022062).

For subcutaneous tumor model experiments, 1 × 10^6^ *lnc191* overexpression or knockdown KYSE‐30 cells and control cells were injected subcutaneously into separate flanks of the mice. Tumor volume was measured every week until the mice were sacrificed 3–4 weeks later. HA15 (MedChemExpress) treatment was performed by intraperitoneal injection when the subcutaneous tumors in the mice reached 5 mm in diameter at 20mg/kg/day for 9 days.

For tail vein metastasis model experiments, 1 × 10^7^ sh‐*lnc191* or sh‐NC KYSE‐30 cells were injected into the tail vein of the mice. After 2 months, the mice were sacrificed and the lung tissues were collected and analyzed by histopathological to determine the number of metastatic tumor nodules.

### Statistical Analysis

Statistical analyses were conducted utilizing SPSS 21.0 software. Data were derived from three independent experiments, with each experiment conducted in triplicate. Quantitative data are reported as the mean ± standard deviation and were analyzed using the Student's *t*‐test or one‐way ANOVA. Categorical data were presented as proportions and were compared using the chi‐square test. A *p*‐value less than 0.05 was considered indicative of statistical significance (*p value < 0.05), and all statistical tests were two‐tailed.

## Conflict of Interest

The authors declare no conflict of interest.

## Author Contributions

S.W. and X.F.: acquisition, analysis, and interpretation of data, statistical analysis, drafting of the manuscript; X.L., W.Z., Z.Z., and S.D.: acquisition, analysis, and interpretation of data; H.L., Y.L., and B.S.: supply of ESCC tissue specimens; L.Z., Q.Z., and Y.S.: funding acquisition, study concept and design, study supervision.

## Supporting information



Supporting Information

## Data Availability

The RNA‐seq data in this study has been deposited in the Sequence Read Archive with accession code PRJNA1124251 and 1123329. The TCGA data was accessed from GDC data portal (https://portal.gdc.cancer.gov).
