# Supplementary material for: Hypoxia Induced Lnc191 Upregulation Dictates the Progression of Esophageal Squamous Cell Carcinoma by Activating GRP78/ERK Pathway
Source: Adv Sci (Weinh). 2024 Dec 4;12(4):2406674. doi: 10.1002/advs.202406674 (PMC11775527; doi:10.1002/advs.202406674)
Supplement: Supplementary file 1 — Supporting Information [file ADVS-12-2406674-s001.docx]

**Supporting Information**

**Hypoxia Induced *Lnc191* Upregulation Dictates the Progression of Esophageal Squamous Cell Carcinoma by Activating GRP78/ERK Pathway**

Sisi Wei^#^, Xinyi Fan^#^, Xiaoya Li, Wei Zhou, Zhihua Zhang, Suli Dai, Huilai Lv, Yueping Liu, Baoen Shan, Lianmei Zhao^*^, Qimin Zhan^*^, Yongmei Song^*^

**
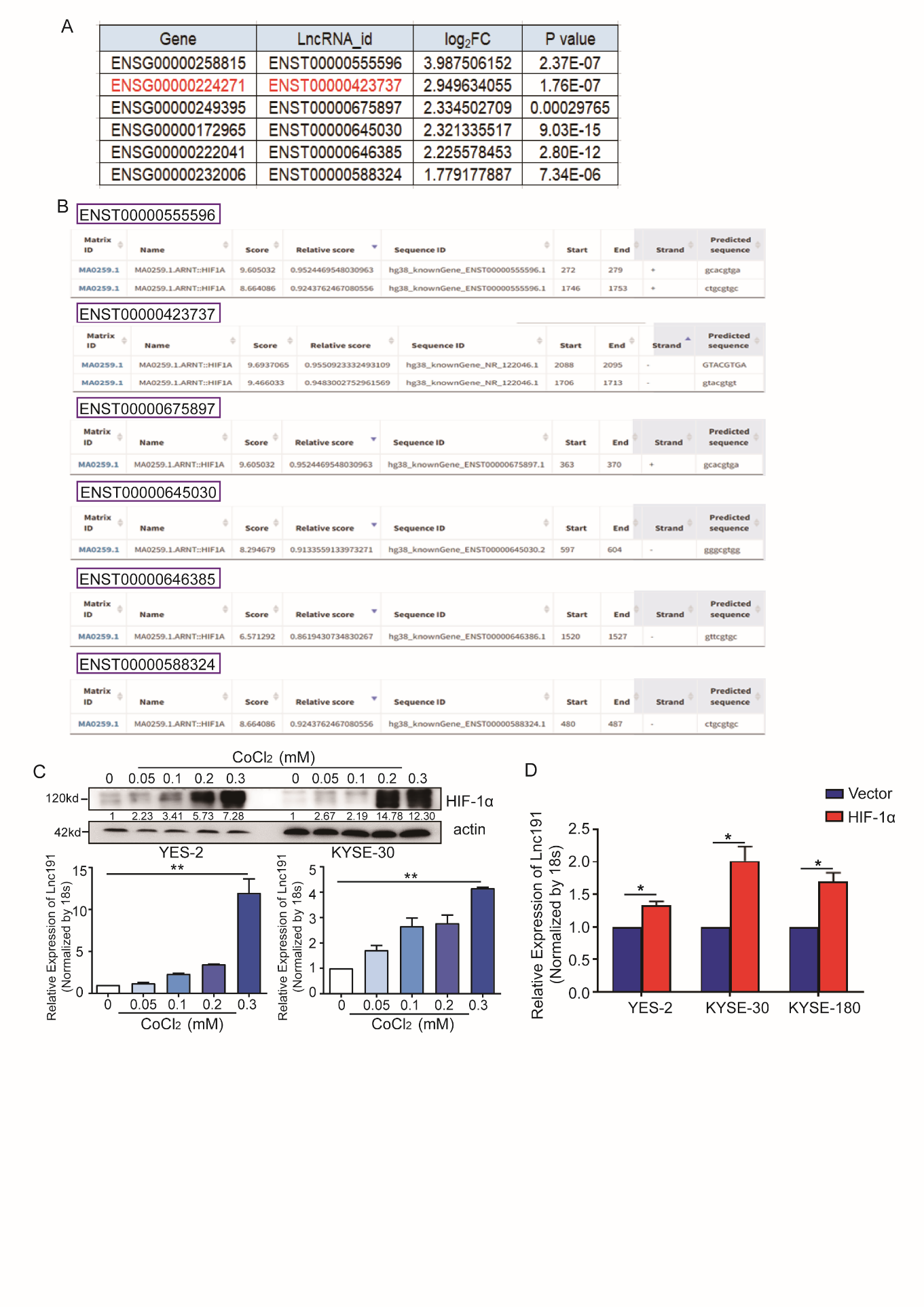
Figure S1. *Lnc191* is transcriptionally upregulated by HIF-1α.**

A) The expression of 6 screened differentially expressed lncRNAs in TCGA dataset. B) The specific binding sites of HIF-1α in promoters of 6 screened differentially expressed lncRNAs. C) Western blot and qPCR were performed to determine the HIF-1α (Top) and *lnc191* level (down) in KYSE-30 and YES-2 cells after treatment with CoCl_2_ (0, 50, 100, 200, 300μM) for 18h. D) QPCR was performed to determine the relative *lnc191* RNA level in YES-2, KYSE-30 and KYSE-180 with HIF-1α plasmid overexpression for 48h. Student’s unpaired t-test was used to calculate the *p* value. Three independent experiments were performed to obtain the mean ± SD value (n = 3 replicate experiments). **p* < 0.05, ***p* < 0.01.


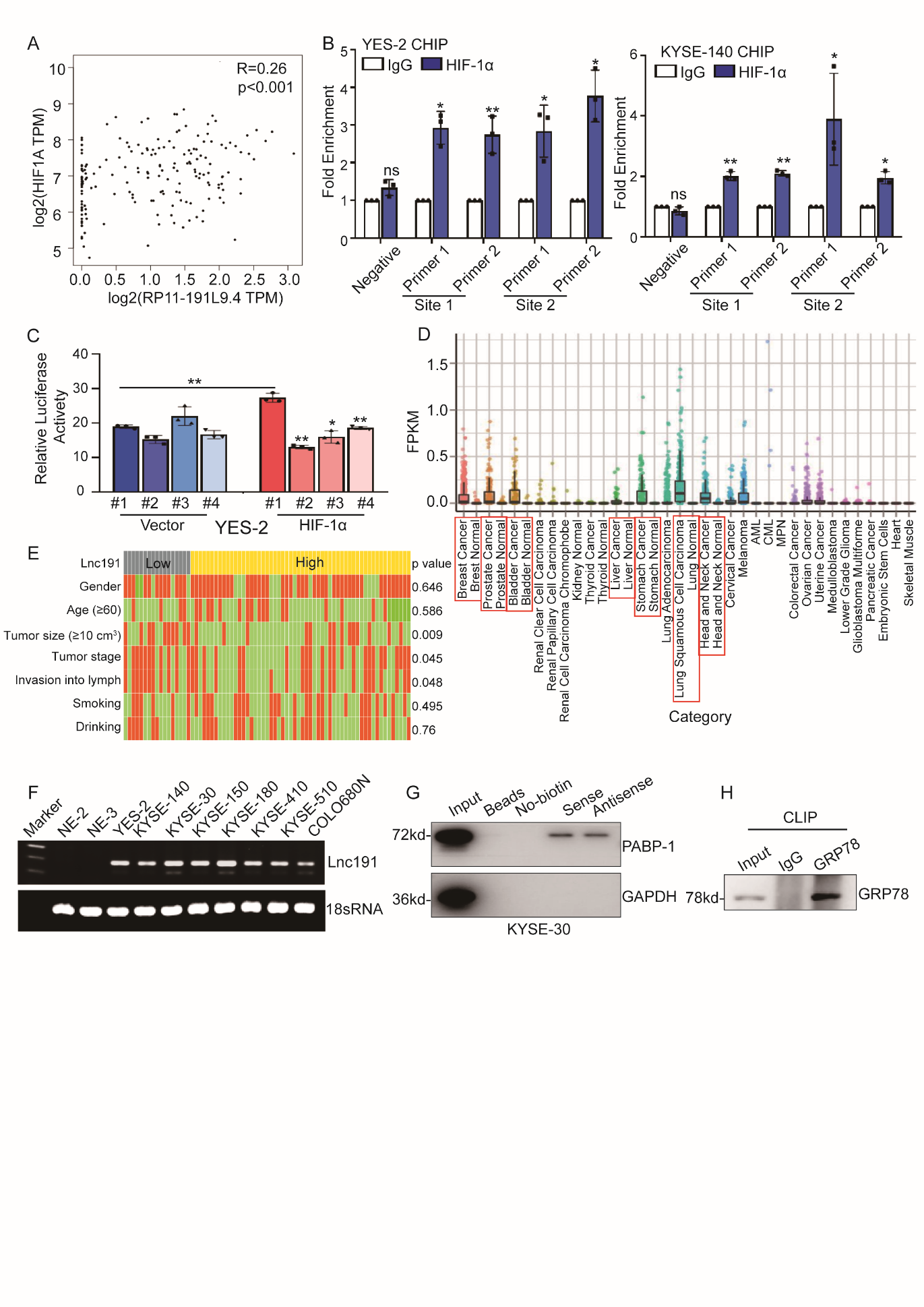


**Figure S2. HIF-1α is the transcriptional factor of *lnc191*.**

A) The correlation between *lnc191* and HIF1A was analyzed in GEPIA. B) CHIP assays investigating the binding capacity of HIF-1α to each HRE was conducted in YES-2 and KYSE-140 cells. C) The relative luciferase reporter activity of HREs on the *lnc191* promoter in YES-2 cells. D) Expression levels of *lnc191* in TCGA normal and tumor samples. Each point represents one tissue sample. E) The relationship between the *lnc191* expression and clinicopathological parameters in ESCC. F) Basal level of *lnc191* was determined using RT-PCR assay in the immortalized esophageal epithelium cell lines NE2 and NE3 and several ESCC cell lines. G) Western blotting of PABP-1 in KYSE-30 and YES-2 protein retrieved from the *lnc191* RNA pull-down assays. H) The efficiency of GRP78 immunoprecipitation was confirmed by western blot. Student’s unpaired t-test was used to calculate the *p* value. Three independent experiments were performed to obtain the mean ± SD value (n = 3 replicate experiments). **p* < 0.05 and ***p* < 0.01, ns: not significant.

**
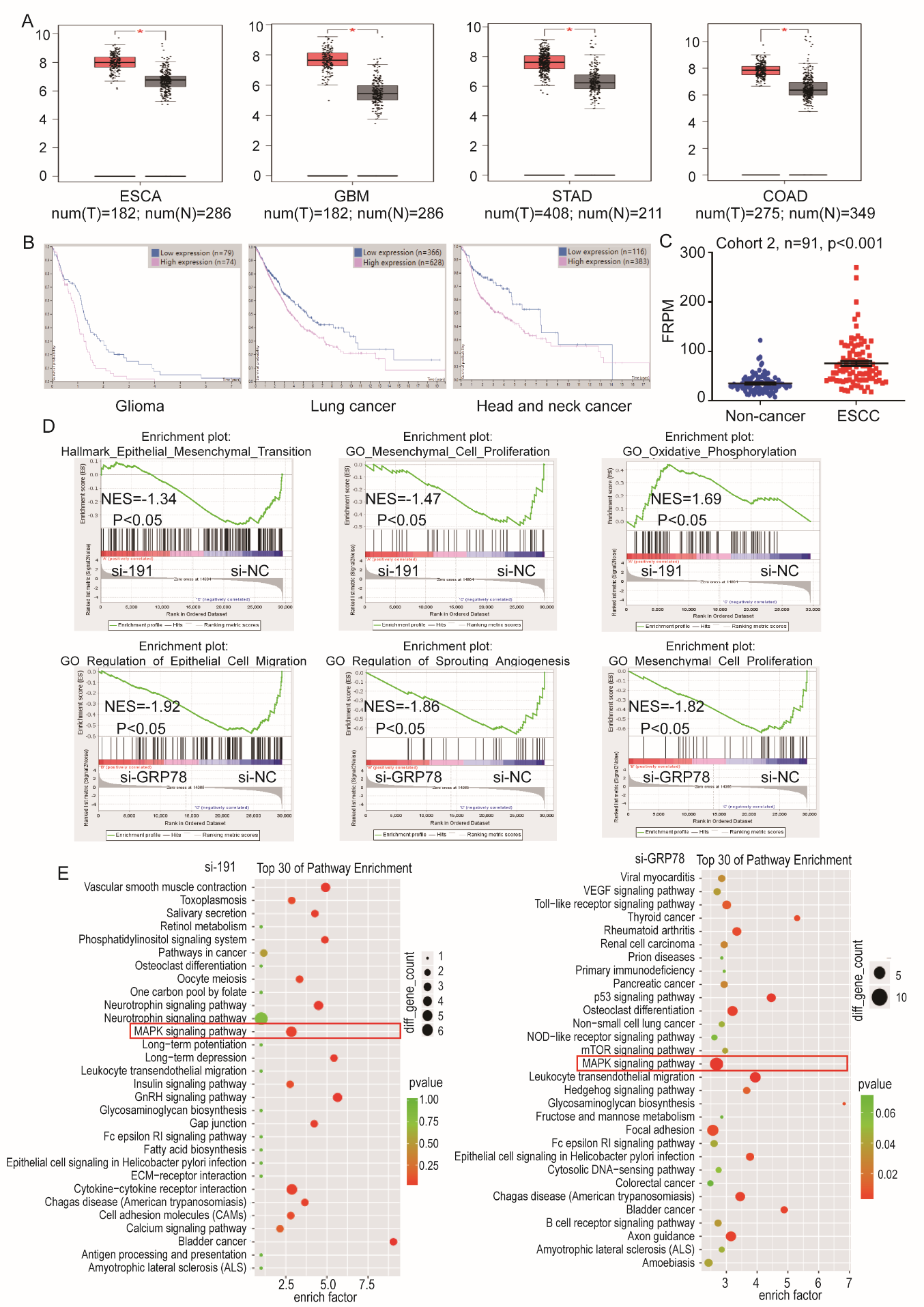
Figure S3. *Lnc191* and GRP78 regulate ERK/MAPK signaling pathway in ESCC.**

A) Boxplots showed the expression of GRP78 in patients with ESCA, GBM, STAD and COAD from GEPIA. B) The overall survival of two groups (GRP78 high expression and GRP78 low expression) in patients with Glioma, Lung cancer and Head and neck cancer. C) The expression of GRP78 in ESCC tissues compared with corresponding normal tissues in Cohort 2. D) Representative GSEA plots from RNA-seq in *lnc191* or GRP78 knockdown. Normalized enrichment score (NES) and p value are indicated. E) KEGG analysis of the top 30 enriched pathways from RNA-seq in *lnc191* or GRP78 knockdown.

**
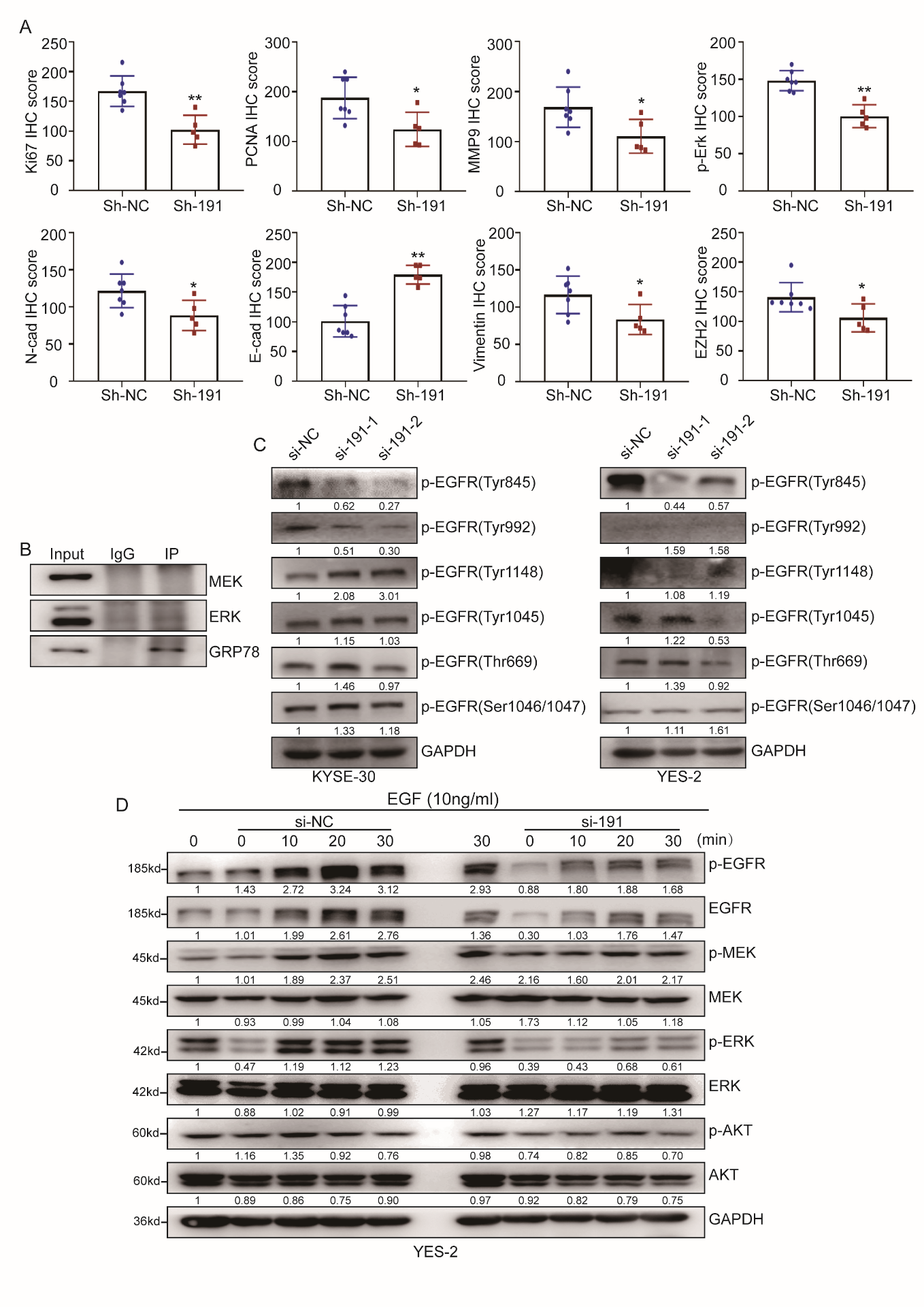
Figure S4. *Lnc191* facilitates the phosphorylation of EGFR at Y845 in ESCC.**

A) The statistic of Ki67, PCNA, MMP9, p-ERK, N-cad, E-cad, Vimentin and EZH2 IHC in Fig 6I. B) The interaction between GRP78 and MEK or ERK were evaluated using IP assays. C) Western blot detected the expression of different phosphorylation sites in KYSE-30 and YES-2 with *lnc191* knockdown. D) YES-2 cells transfected with either control siRNA or *lnc191* siRNA were stimulated with 10ng/ml of EGF for indicated duration. Cell lysates were analyzed by western blotting.


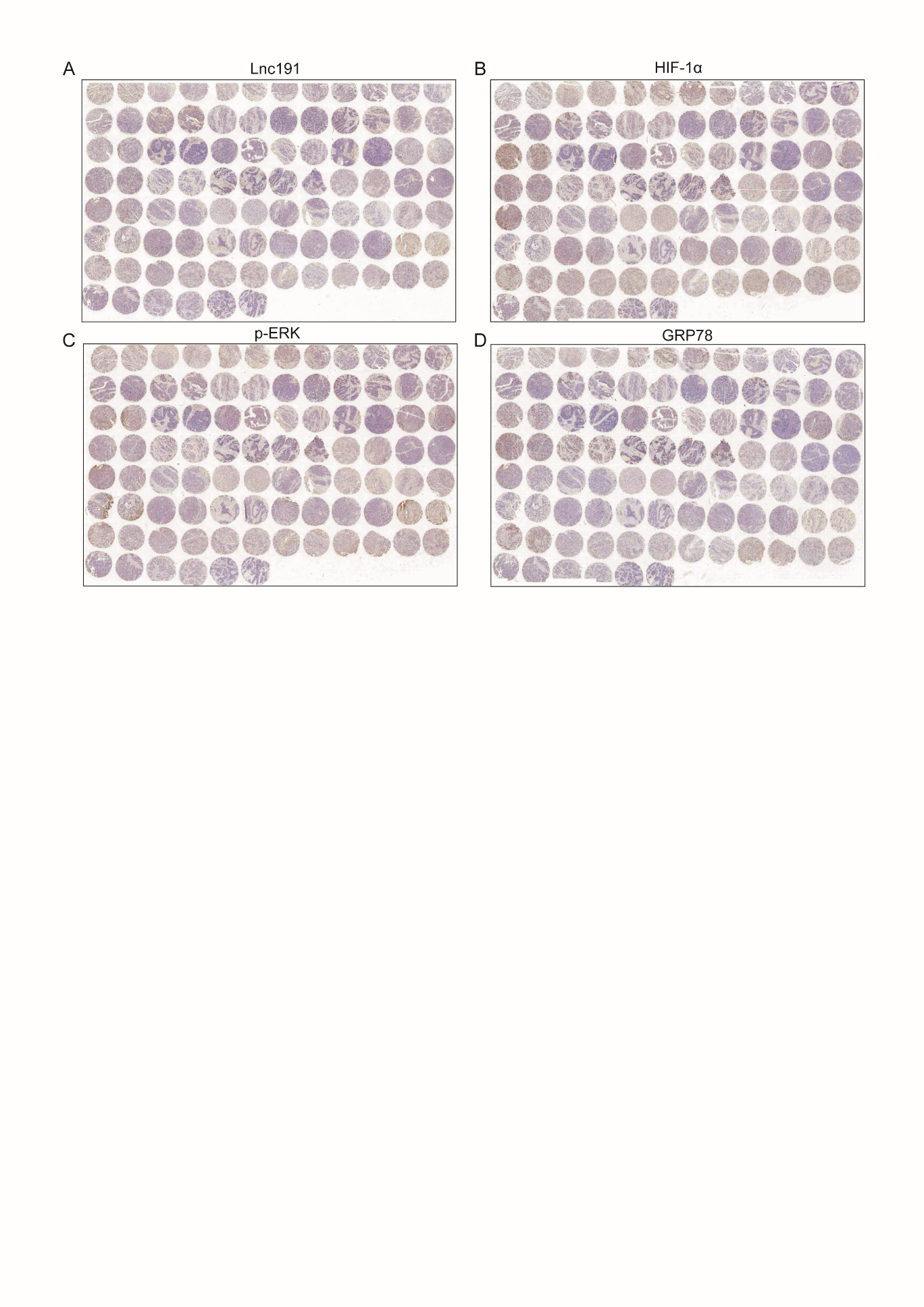


**Figure S5. The expression of HIF-1α/*Lnc191*/p-ERK axis in ESCC tissues.**

A-D) The expression of *lnc191* (A), HIF-1α (B), p-ERK (C) and GRP78 (D) were detected in ESCC tissue chips.

**Supplemental table S1. Si sequences and primer sequences for q-PCR, CHIP and RACE.**

| siRNA | 5’-3’ |
| --- | --- |
| Si-lnc191-1 | CCCUUCUGGGUGUAAAGUUTT |
| Si-lnc191-1 | GCUUCAGGCUUGACCAAAUTT |
| Si-GRP78-1 | GCCACCAAGAUGCUGACAUTT |
| Si-GRP78-2 | GGUUACCCAUGCAGUUGUUTT |

| Gene name | Forward Sequence (5'-3') | Reverse Sequence (5'-3') |
| --- | --- | --- |
| lnc191 | CTCCGGGTTTCAGATCCCAC | CCGATGGGTAGCAGTGTCTC |
| 172965 | CCTGATAACGGGAACCAGCG | GGAGGGGGATGAGTCGTGAT |
| 222041 | TGGCACAGTCTTTTCTCTACTC | TGCAGACGGAGGTTGGAATG |
| 232006 | GACCACTTTCAGGTGTCCCC | GCATCCTGGCTCCTTCATCA |
| 249395 | TTGGTCAGCCACATTCATGGT | GCCAATGACTCTCCAGCCAA |
| 258815 | CTTCTTCTGTCAACCTGGTGC | CAGAGGTCTGATCCTGTATGGC |
| ACTIN | CTCCATCCTGGCCTCGCTGT | GCTGTCACCTTCACCGTTCC |
| 18sRNA | CAGCCACCCGAGATTGAGCA | TAGTAGCGACGGGCGGGTGT |

| CHIP | Forward Sequence (5'-3') | Reverse Sequence (5'-3') |
| --- | --- | --- |
| Site1-1 | GGGAGGGGAAGATGTGAGTG | GGACAGCAGATCGTGGGACT |
| Site1-2 | AGGGTTCTGCAGAGAAACAGAG | AACTTGCACTGCCAGCATTC |
| Site2-1 | CAGTCCGCCATTGCAAACAC | GCTGAGGCCACACTTCACGTT |
| Site2-2 | CCAGAAACGCCCTCACAGAC | ACACACACTCCTCCCATGCAC |

| RACE | 5’-3’ |
| --- | --- |
| 3’RACE-1 | TGCTCTTTAAGTCACTGGGCGG |
| 3’RACE-2 | AACTCTTGGCTACCTCACCCCC |
| 5’RACE-1 | AGCCTCATCTGAGCCGCTG |
| 5’RACE-2 | CCTGAAGCCTCATCTGAGCCGC |

**Supplemental table S2. Upregulated lncRNAs in CoCl_2_ induction and overexpression of HIF-1α plasmid models.**

| Gene | lncRNA_id | 18h_FPKM | 0h_FPKM | HIF_FPKM | NC_FPKM |
| --- | --- | --- | --- | --- | --- |
| ENSG00000172965 | ENST00000308604 | 0.18009771 | 0.08056366 | 0.49865807 | 0.126859747 |
| ENSG00000175611 | ENST00000321517 | 0.86170538 | 0.03504424 | 0.433820732 | 0.05518249 |
| ENSG00000259728 | ENST00000339094 | 0.39303184 | 0.02068425 | 0.469448869 | 0.065108435 |
| ENSG00000198496 | ENST00000356906 | 0.55907632 | 0.13637132 | 1.313470677 | 0.07160182 |
| ENSG00000115355 | ENST00000366287 | 0.13434316 | 0.04006409 | 0.165348923 | 0.063087009 |
| ENSG00000263528 | ENST00000367119 | 0.80629186 | 0.33935334 | 0.481543448 | 0.233800577 |
| ENSG00000204177 | ENST00000374336 | 0.33585789 | 0.1334835 | 0.82667449 | 0.052572508 |
| ENSG00000186312 | ENST00000380331 | 1.52009076 | 0.06799869 | 1.262657131 | 0.107074282 |
| ENSG00000205763 | ENST00000381639 | 1.52588607 | 0.51175247 | 7.394193649 | 1.12821796 |
| ENSG00000169031 | ENST00000396588 | 0.29186566 | 0.12047981 | 0.207225185 | 0.031628988 |
| ENSG00000225549 | ENST00000404721 | 0.5250736 | 0.15658852 | 0.646258113 | 0.246572466 |
| ENSG00000141013 | ENST00000409873 | 0.99483985 | 0.39543537 | 0.61214067 | 0.207588062 |
| ENSG00000157212 | ENST00000411526 | 0.15730053 | 0.03518286 | 0.580740358 | 0.276893077 |
| ENSG00000064655 | ENST00000414085 | 1.41012675 | 0.42033101 | 0.867788672 | 0.330929395 |
| ENSG00000275266 | ENST00000414790 | 1.89476017 | 0.13179634 | 17.25565919 | 4.595653759 |
| ENSG00000204256 | ENST00000415875 | 0.4411861 | 0.13157142 | 1.357386268 | 0.621330517 |
| ENSG00000203497 | ENST00000416249 | 0.4958301 | 0.1109006 | 0.457699013 | 0.174629876 |
| ENSG00000218510 | ENST00000416769 | 2.99986497 | 0.82551961 | 1.363149142 | 0.194995092 |
| ENSG00000206341 | ENST00000420084 | 2.1411728 | 0.95749309 | 0.564634368 | 0.143572445 |
| ENSG00000261105 | ENST00000422931 | 0.11492941 | 0.05141172 | 0.212181854 | 0.080955584 |
| ENSG00000256274 | ENST00000422992 | 1.50134405 | 0.19180153 | 1.187842957 | 0.151006617 |
| ENSG00000205485 | ENST00000423084 | 0.86389115 | 0.35660735 | 1.840093149 | 0.561523301 |
| ENSG00000224271 | ENST00000423737 | 0.35602787 | 0.04898072 | 0.758341419 | 0.308541357 |
| ENSG00000252105 | ENST00000424684 | 0.06636444 | 0.008482 | 0.087506496 | 0.026699053 |
| ENSG00000233325 | ENST00000424756 | 0.28649549 | 0.12815891 | 0.528925948 | 0.201805707 |
| ENSG00000215837 | ENST00000425425 | 0.85211945 | 0.3386667 | 1.398201548 | 0.266634541 |
| ENSG00000232915 | ENST00000427150 | 0.47604438 | 0.10644146 | 0.219680171 | 0.055887135 |
| ENSG00000182109 | ENST00000427240 | 0.12228036 | 0.05470005 | 0.225753141 | 0.086133555 |
| ENSG00000243004 | ENST00000428100 | 0.52992503 | 0.09482119 | 0.391337535 | 0.149310406 |
| ENSG00000226383 | ENST00000428651 | 3.34884905 | 0.99835885 | 1.545493882 | 0.262020854 |
| ENSG00000273974 | ENST00000429124 | 0.86128779 | 0.17117532 | 0.706656232 | 0.134768136 |
| ENSG00000196204 | ENST00000429902 | 0.1499456 | 0.06707563 | 0.968829355 | 0.316756734 |
| ENSG00000188738 | ENST00000429929 | 0.45188153 | 0.20204547 | 2.085438902 | 0.954589612 |
| ENSG00000234450 | ENST00000431047 | 1.14796692 | 0.30801651 | 0.423873681 | 0.161724204 |
| ENSG00000185973 | ENST00000433624 | 0.49648558 | 0.1305941 | 0.161726942 | 0.041143702 |
| ENSG00000205885 | ENST00000435921 | 0.34760117 | 0.07774675 | 0.320869413 | 0.122424092 |
| ENSG00000203739 | ENST00000437190 | 0.09784836 | 0.04377081 | 1.174067295 | 0.137778675 |
| ENSG00000241990 | ENST00000439423 | 0.71551097 | 0.30113658 | 0.310816772 | 0.05929431 |
| ENSG00000228348 | ENST00000442409 | 0.32207539 | 0.09604998 | 0.396408864 | 0.151245314 |
| ENSG00000120658 | ENST00000444442 | 0.05089016 | 0.02275403 | 0.164405903 | 0.07167553 |
| ENSG00000172476 | ENST00000445990 | 0.15730053 | 0.07036573 | 0.290407127 | 0.110801551 |
| ENSG00000136002 | ENST00000446213 | 3.70939796 | 1.15239441 | 3.459880818 | 1.484750535 |
| ENSG00000131374 | ENST00000449613 | 0.86824398 | 0.29120338 | 0.400736472 | 0.152896464 |
| ENSG00000276991 | ENST00000450963 | 0.45325503 | 0.13510639 | 0.278932073 | 0.106423374 |
| ENSG00000235052 | ENST00000451217 | 0.43602603 | 0.19495616 | 0.402494088 | 0.153567062 |
| ENSG00000088340 | ENST00000452272 | 0.38236129 | 0.17104285 | 0.705912708 | 0.269333002 |
| ENSG00000163904 | ENST00000455173 | 0.99746424 | 0.44605735 | 1.53443665 | 0.468171017 |
| ENSG00000143390 | ENST00000455503 | 1.04561583 | 0.35966206 | 1.039375182 | 0.113311619 |
| ENSG00000115109 | ENST00000455707 | 0.5186814 | 0.07734111 | 0.319195311 | 0.121785357 |
| ENSG00000123983 | ENST00000456830 | 1.19487903 | 0.42740388 | 0.441195442 | 0.168333126 |
| ENSG00000179818 | ENST00000457770 | 0.18174394 | 0.08130007 | 0.335534377 | 0.128019343 |
| ENSG00000174839 | ENST00000470427 | 0.27311521 | 0.12217346 | 0.504223363 | 0.192380715 |
| ENSG00000243648 | ENST00000471205 | 0.19962638 | 0.08929948 | 0.368548803 | 0.140615623 |
| ENSG00000212951 | ENST00000473402 | 0.87014386 | 0.19452956 | 0.803226714 | 0.306308828 |
| ENSG00000243007 | ENST00000477007 | 0.20043132 | 0.08965956 | 0.370034887 | 0.141182622 |
| ENSG00000241709 | ENST00000486808 | 0.49706968 | 0.14823713 | 0.917530848 | 0.233421935 |
| ENSG00000230561 | ENST00000499346 | 0.10805863 | 0.03222546 | 0.398926456 | 0.101437054 |
| ENSG00000133812 | ENST00000499953 | 0.17461464 | 0.05207393 | 0.214914876 | 0.081998338 |
| ENSG00000183323 | ENST00000508046 | 0.79214291 | 0.3541833 | 0.913937368 | 0.278850865 |
| ENSG00000055163 | ENST00000508443 | 0.07718473 | 0.02301819 | 0.189997209 | 0.072455038 |
| ENSG00000120306 | ENST00000508713 | 0.06572822 | 0.0147012 | 0.060673489 | 0.023149283 |
| ENSG00000109790 | ENST00000509449 | 0.47289927 | 0.1409843 | 0.872915183 | 0.369971299 |
| ENSG00000240376 | ENST00000510327 | 0.1606041 | 0.07184352 | 1.185873688 | 0.226143997 |
| ENSG00000253540 | ENST00000511564 | 0.57244109 | 0.17063341 | 0.352278895 | 0.134408024 |
| ENSG00000143436 | ENST00000512280 | 0.2602459 | 0.1164166 | 0.720573965 | 0.183315655 |
| ENSG00000252827 | ENST00000517018 | 0.69278004 | 0.15495171 | 3.197188561 | 1.219487278 |
| ENSG00000253923 | ENST00000517692 | 0.25490753 | 0.04561143 | 0.376486778 | 0.071822134 |
| ENSG00000196611 | ENST00000525739 | 0.20695558 | 0.08412988 | 0.243124096 | 0.052983775 |
| ENSG00000168040 | ENST00000527232 | 2.62700363 | 0.80377394 | 1.403776172 | 0.681566077 |
| ENSG00000160972 | ENST00000528207 | 0.28403982 | 0.1185414 | 0.699118565 | 0.106680493 |
| ENSG00000160172 | ENST00000529253 | 2.1948697 | 0.23938735 | 0.889417841 | 0.263891228 |
| ENSG00000137502 | ENST00000533708 | 0.24100348 | 0.10775759 | 0.556117041 | 0.084880704 |
| ENSG00000205885 | ENST00000535078 | 0.30588903 | 0.13683428 | 0.564730166 | 0.215466401 |
| ENSG00000255717 | ENST00000537068 | 1.02733002 | 0.49470773 | 2.334040741 | 0.890306142 |
| ENSG00000256268 | ENST00000541391 | 0.40610268 | 0.07266526 | 1.199437603 | 0.114422517 |
| ENSG00000231887 | ENST00000541449 | 0.15813457 | 0.04715922 | 0.486528694 | 0.074259364 |
| ENSG00000153179 | ENST00000545821 | 0.20625298 | 0.09226378 | 0.380782788 | 0.145283362 |
| ENSG00000257698 | ENST00000546580 | 1.65399718 | 0.15569178 | 1.928424555 | 0.612947196 |
| ENSG00000106992 | ENST00000548587 | 0.53563543 | 0.09584297 | 1.582016838 | 0.754294933 |
| ENSG00000167566 | ENST00000548872 | 0.50060651 | 0.10109524 | 0.298113423 | 0.022750676 |
| ENSG00000062485 | ENST00000549565 | 1.24578867 | 0.55707057 | 1.609859305 | 0.350834923 |
| ENSG00000006432 | ENST00000554032 | 0.27014656 | 0.12084549 | 1.994716883 | 0.190289621 |
| ENSG00000119707 | ENST00000554385 | 0.58364346 | 0.17397262 | 0.359172806 | 0.137038318 |
| ENSG00000279765 | ENST00000554669 | 0.94598036 | 0.15380624 | 0.476227084 | 0.121153253 |
| ENSG00000183032 | ENST00000556667 | 4.13353336 | 1.68669938 | 2.384548951 | 1.01873388 |
| ENSG00000133997 | ENST00000557241 | 0.65242944 | 0.25526757 | 0.602178682 | 0.172280324 |
| ENSG00000258446 | ENST00000557756 | 1.64049399 | 0.44016881 | 0.605733677 | 0.231110826 |
| ENSG00000245534 | ENST00000558140 | 0.51480174 | 0.05117508 | 1.267123615 | 0.241668256 |
| ENSG00000253438 | ENST00000561978 | 0.27448627 | 0.11158191 | 0.737003828 | 0.316279691 |
| ENSG00000185596 | ENST00000562189 | 0.42124549 | 0.05381356 | 0.388821951 | 0.042388971 |
| ENSG00000267127 | ENST00000562391 | 0.18409988 | 0.03294159 | 0.271907117 | 0.051871541 |
| ENSG00000260302 | ENST00000563722 | 0.11714132 | 0.03493412 | 0.36040578 | 0.055009097 |
| ENSG00000260001 | ENST00000564226 | 0.6876458 | 0.17569171 | 0.543990621 | 0.138392451 |
| ENSG00000261541 | ENST00000565549 | 0.03595874 | 0.01072369 | 0.066375513 | 0.016886082 |
| ENSG00000260996 | ENST00000566954 | 0.83110401 | 0.21581092 | 0.247463084 | 0.075503309 |
| ENSG00000260265 | ENST00000567197 | 0.5223149 | 0.15569178 | 1.928424555 | 0.367792845 |
| ENSG00000259820 | ENST00000568248 | 2.55166213 | 1.03085688 | 1.012527821 | 0.481383745 |
| ENSG00000157349 | ENST00000570278 | 0.22415769 | 0.10022549 | 0.827571379 | 0.236764053 |
| ENSG00000141258 | ENST00000573007 | 0.44983681 | 0.10061344 | 0.622758494 | 0.158431177 |
| ENSG00000135740 | ENST00000573063 | 0.03713632 | 0.0166123 | 0.068560816 | 0.026158603 |
| ENSG00000133392 | ENST00000574212 | 0.17837429 | 0.05319514 | 0.439084459 | 0.16744394 |
| ENSG00000177337 | ENST00000574411 | 8.89340763 | 2.94190701 | 0.899692667 | 0.343095917 |
| ENSG00000141219 | ENST00000579037 | 0.30970073 | 0.13853938 | 0.571767302 | 0.218151341 |
| ENSG00000128487 | ENST00000580225 | 0.76472258 | 0.22805713 | 0.941216944 | 0.359110669 |
| ENSG00000252072 | ENST00000580623 | 0.17752488 | 0.07941275 | 0.327745186 | 0.125047465 |
| ENSG00000227543 | ENST00000582858 | 1.30236597 | 0.38820964 | 0.601002739 | 0.152896464 |
| ENSG00000204650 | ENST00000583740 | 0.46282093 | 0.08281404 | 0.512587066 | 0.130403315 |
| ENSG00000155657 | ENST00000585487 | 4.87181097 | 1.94304382 | 2.673806011 | 0.463566687 |
| ENSG00000266504 | ENST00000585761 | 0.53257465 | 0.07941275 | 0.327745186 | 0.125047465 |
| ENSG00000130023 | ENST00000586101 | 0.7013565 | 0.21818546 | 0.506612125 | 0.085879223 |
| ENSG00000196628 | ENST00000586467 | 0.2234021 | 0.09993515 | 0.618560122 | 0.157363102 |
| ENSG00000088053 | ENST00000586845 | 0.80897416 | 0.40193699 | 0.995512674 | 0.253134058 |
| ENSG00000214425 | ENST00000587960 | 0.65922187 | 0.29478933 | 0.527344108 | 0.185684982 |
| ENSG00000232006 | ENST00000588324 | 0.1606041 | 0.03592176 | 0.148253073 | 0.056564281 |
| ENSG00000235641 | ENST00000588550 | 0.11920136 | 0.05332271 | 0.550115778 | 0.251810209 |
| ENSG00000267284 | ENST00000589662 | 0.77515739 | 0.25998274 | 2.861816035 | 0.272871607 |
| ENSG00000186577 | ENST00000593917 | 0.70340049 | 0.20966983 | 1.29839271 | 0.165157029 |
| ENSG00000268628 | ENST00000598007 | 0.69369605 | 0.05394418 | 0.50107874 | 0.212374301 |
| ENSG00000130304 | ENST00000598141 | 0.52213201 | 0.09342677 | 0.963858316 | 0.441196879 |
| ENSG00000141965 | ENST00000598782 | 0.59458095 | 0.21267944 | 0.658514963 | 0.167527704 |
| ENSG00000151208 | ENST00000601701 | 1.10790691 | 0.22519874 | 1.394518415 | 0.354602684 |
| ENSG00000227769 | ENST00000602182 | 0.27513082 | 0.08205007 | 0.338629712 | 0.129200333 |
| ENSG00000171044 | ENST00000602575 | 2.73948656 | 1.21401439 | 0.273345834 | 0.034769901 |
| ENSG00000154511 | ENST00000602631 | 0.33594214 | 0.15675649 | 0.161774466 | 0.041135214 |
| ENSG00000154358 | ENST00000602778 | 0.39036886 | 0.11636127 | 0.240232073 | 0.091657828 |
| ENSG00000253352 | ENST00000602847 | 1.18349923 | 0.56991056 | 0.672297817 | 0.256379367 |
| ENSG00000109670 | ENST00000603766 | 0.69450853 | 0.26620988 | 1.098839339 | 0.279407454 |
| ENSG00000223509 | ENST00000604301 | 0.63320978 | 0.07081392 | 0.584513707 | 0.111507294 |
| ENSG00000007001 | ENST00000604340 | 1.40727636 | 0.05036369 | 1.558765204 | 0.079305301 |
| ENSG00000271270 | ENST00000605830 | 0.45496962 | 0.08900777 | 0.209970198 | 0.080101915 |
| ENSG00000170873 | ENST00000606244 | 0.73242094 | 0.30566912 | 1.171760679 | 0.41259276 |
| ENSG00000196821 | ENST00000606496 | 3.26826866 | 1.21792414 | 4.776358576 | 0.958884517 |
| ENSG00000198976 | ENST00000606993 | 3.02165035 | 1.26112151 | 2.696012155 | 0.141826884 |
| ENSG00000272024 | ENST00000607201 | 0.97704114 | 0.43685507 | 0.450951607 | 0.17205548 |
| ENSG00000066777 | ENST00000607397 | 0.62524488 | 0.07987422 | 0.741938691 | 0.188687974 |
| ENSG00000084693 | ENST00000607407 | 0.15085574 | 0.06748276 | 0.696201151 | 0.106261882 |
| ENSG00000185630 | ENST00000607752 | 0.22936636 | 0.05860251 | 0.120987017 | 0.046161226 |
| ENSG00000271751 | ENST00000607857 | 0.23955165 | 0.10715937 | 0.442258564 | 0.168738748 |
| ENSG00000283930 | ENST00000608335 | 0.09852719 | 0.01888307 | 0.168887494 | 0.059467567 |
| ENSG00000116191 | ENST00000608517 | 1.10457445 | 0.17961733 | 0.880490344 | 0.42426205 |
| ENSG00000135899 | ENST00000609120 | 0.10553496 | 0.04720928 | 0.292207276 | 0.074338196 |
| ENSG00000226328 | ENST00000609284 | 1.68016628 | 0.18784669 | 7.237301541 | 0.197159344 |
| ENSG00000160255 | ENST00000609461 | 0.54029313 | 0.09667639 | 0.398994139 | 0.152231697 |
| ENSG00000164654 | ENST00000609497 | 0.1574999 | 0.05633714 | 0.581491202 | 0.177462798 |
| ENSG00000277778 | ENST00000613309 | 0.21897343 | 0.0653027 | 0.26951146 | 0.102829046 |
| ENSG00000225871 | ENST00000614292 | 1.09112856 | 0.0542331 | 1.119015996 | 0.085398269 |
| ENSG00000103423 | ENST00000616838 | 0.76866445 | 0.30549831 | 0.315356193 | 0.120320585 |
| ENSG00000183751 | ENST00000618631 | 0.61620208 | 0.18376504 | 0.758418612 | 0.289366035 |
| ENSG00000121060 | ENST00000619432 | 1.57119726 | 0.25545979 | 2.63676183 | 0.804701997 |
| ENSG00000264230 | ENST00000623463 | 1.09794507 | 0.5356271 | 0.737097607 | 0.281090631 |
| ENSG00000180336 | ENST00000623480 | 0.08562785 | 0.02552398 | 0.105390355 | 0.040190392 |
| ENSG00000129595 | ENST00000623705 | 0.49849395 | 0.03185612 | 0.262947427 | 0.050162307 |
| ENSG00000180773 | ENST00000623872 | 0.64303969 | 0.28754354 | 0.356092179 | 0.09059066 |
| ENSG00000203875 | ENST00000623910 | 0.47264946 | 0.21136474 | 0.727094387 | 0.332820467 |
| ENSG00000140836 | ENST00000624370 | 0.74051349 | 0.20695638 | 0.683478411 | 0.260741429 |
| ENSG00000145431 | ENST00000624427 | 0.91205445 | 0.20399606 | 0.841914239 | 0.321222846 |
| ENSG00000129636 | ENST00000624660 | 0.86970468 | 0.29915364 | 0.617492003 | 0.188402603 |
| ENSG00000170296 | ENST00000624722 | 0.67709234 | 0.1376292 | 0.284084556 | 0.086676863 |
| ENSG00000113369 | ENST00000625713 | 0.57658116 | 0.12446847 | 0.220207404 | 0.084003886 |
| ENSG00000228956 | ENST00000626982 | 0.13525706 | 0.06050495 | 0.624213005 | 0.190453244 |
| ENSG00000283208 | ENST00000630676 | 0.35632235 | 0.07969738 | 0.657839799 | 0.125495664 |
| ENSG00000149084 | ENST00000637880 | 0.61824587 | 0.05531236 | 0.342362257 | 0.087097737 |
| ENSG00000162576 | NONHSAT000290.2 | 0.31196842 | 0.09303586 | 0.767938511 | 0.146499122 |
| ENSG00000178585 | NONHSAT000821.2 | 0.25721587 | 0.11502471 | 0.553934593 | 0.12075735 |
| ENSG00000076864 | NONHSAT001428.2 | 0.05920517 | 0.00756698 | 0.062459522 | 0.023818808 |
| ENSG00000116478 | NONHSAT002195.2 | 0.52117397 | 0.11651377 | 0.481093851 | 0.091777956 |
| ENSG00000162526 | NONHSAT002201.2 | 0.20121611 | 0.05682777 | 0.156398383 | 0.067117199 |
| ENSG00000163866 | NONHSAT002317.2 | 0.58905249 | 0.28365648 | 0.585536794 | 0.127605409 |
| ENSG00000116898 | NONHSAT002418.2 | 1.79860739 | 0.73115512 | 3.320115353 | 1.381642859 |
| ENSG00000237950 | NONHSAT002827.2 | 0.01790597 | 0.00533995 | 0.077129426 | 0.016808734 |
| ENSG00000117862 | NONHSAT003230.2 | 0.13290633 | 0.0594534 | 0.245370727 | 0.093618423 |
| ENSG00000117620 | NONHSAT004770.2 | 0.14366176 | 0.06426465 | 0.397773489 | 0.10119448 |
| ENSG00000117543 | NONHSAT004816.2 | 1.68685097 | 0.77108278 | 0.553586586 | 0.211188759 |
| ENSG00000178104 | NONHSAT005870.2 | 0.36335503 | 0.06501629 | 0.670755202 | 0.102378042 |
| ENSG00000284738 | NONHSAT006552.2 | 0.27584333 | 0.09866816 | 0.407408 | 0.077720955 |
| ENSG00000000457 | NONHSAT007480.2 | 0.8860203 | 0.31697175 | 0.981358039 | 0.062381076 |
| ENSG00000214796 | NONHSAT008854.2 | 0.34007047 | 0.07603828 | 0.366210868 | 0.079807944 |
| ENSG00000186007 | NONHSAT008996.2 | 0.40296478 | 0.17236992 | 2.005288193 | 0.296101113 |
| ENSG00000230937 | NONHSAT009216.2 | 0.73668673 | 0.20806731 | 4.795475165 | 1.583518382 |
| ENSG00000136636 | NONHSAT009473.2 | 0.59074495 | 0.1509338 | 0.778941525 | 0.237662367 |
| ENSG00000233117 | NONHSAT011084.2 | 0.12444891 | 0.01854788 | 0.344596063 | 0.116837589 |
| ENSG00000227821 | NONHSAT011267.2 | 0.4119362 | 0.12284845 | 0.760384681 | 0.193443592 |
| ENSG00000148426 | NONHSAT011429.2 | 0.08550511 | 0.03823714 | 0.315691061 | 0.040132779 |
| ENSG00000223482 | NONHSAT015418.2 | 0.71585192 | 0.04002803 | 0.49551621 | 0.125997421 |
| ENSG00000171862 | NONHSAT015462.2 | 0.44932852 | 0.07535093 | 0.466541109 | 0.197736073 |
| ENSG00000173124 | NONHSAT015753.2 | 0.29452624 | 0.14182824 | 0.083654195 | 0.031917311 |
| ENSG00000280693 | NONHSAT016207.2 | 0.22113826 | 0.02198277 | 0.181450622 | 0.034615215 |
| ENSG00000177830 | NONHSAT017356.2 | 0.35454328 | 0.12681884 | 0.392666554 | 0.099895264 |
| ENSG00000005801 | NONHSAT017576.2 | 0.7701914 | 0.05300493 | 1.749835073 | 0.667464319 |
| ENSG00000133805 | NONHSAT017961.2 | 0.34146102 | 0.13088435 | 1.080599039 | 0.41218786 |
| ENSG00000149100 | NONHSAT018668.2 | 1.58302445 | 0.37749472 | 2.727334017 | 0.297204107 |
| ENSG00000174516 | NONHSAT022303.2 | 0.34022969 | 0.03803209 | 0.314034572 | 0.07486478 |
| ENSG00000174165 | NONHSAT022314.2 | 0.29253733 | 0.08719962 | 1.305033839 | 0.377642118 |
| ENSG00000077514 | NONHSAT022908.2 | 0.4631084 | 0.13810913 | 0.569991627 | 0.217473852 |
| ENSG00000246174 | NONHSAT023262.2 | 0.11745503 | 0.02101661 | 0.130084714 | 0.033093847 |
| ENSG00000256673 | NONHSAT026823.2 | 0.29713815 | 0.11389509 | 0.470126822 | 0.179342199 |
| ENSG00000139112 | NONHSAT026871.2 | 0.14263118 | 0.06380364 | 0.263324683 | 0.100468552 |
| ENSG00000111266 | NONHSAT026972.2 | 0.13470723 | 0.04015357 | 0.290123696 | 0.063226348 |
| ENSG00000050405 | NONHSAT028181.2 | 5.23222892 | 0.07550278 | 0.311608326 | 0.118890629 |
| ENSG00000149948 | NONHSAT029212.2 | 0.35298269 | 0.00444589 | 0.022944417 | 0.003502029 |
| ENSG00000258815 | NONHSAT029748.2 | 0.26628733 | 0.07941275 | 0.327745186 | 0.125047465 |
| ENSG00000257252 | NONHSAT029944.2 | 0.46147052 | 0.17688481 | 0.243418175 | 0.092873449 |
| ENSG00000246985 | NONHSAT029955.2 | 0.288994 | 0.12927657 | 1.067077349 | 0.203565641 |
| ENSG00000102710 | NONHSAT033234.2 | 2.46295786 | 0.20032045 | 4.133302329 | 0.315435047 |
| ENSG00000150907 | NONHSAT033339.2 | 0.08788361 | 0.0157253 | 0.259567119 | 0.123759848 |
| ENSG00000239827 | NONHSAT033360.2 | 0.45662569 | 0.17502775 | 0.602095221 | 0.091898399 |
| ENSG00000231607 | NONHSAT033790.2 | 0.62356411 | 0.03984869 | 0.493296155 | 0.062747832 |
| ENSG00000102805 | NONHSAT034393.2 | 0.069278 | 0.03099034 | 0.12790056 | 0.048799011 |
| ENSG00000134900 | NONHSAT035005.2 | 0.1865177 | 0.08343554 | 0.344347662 | 0.131381952 |
| ENSG00000269125 | NONHSAT035327.2 | 0.06860865 | 0.03069092 | 0.126664806 | 0.048327523 |
| ENSG00000225766 | NONHSAT035962.2 | 0.29543517 | 0.03303948 | 0.136357581 | 0.052025691 |
| ENSG00000100926 | NONHSAT036007.2 | 0.13046448 | 0.05833334 | 0.301047611 | 0.09185245 |
| ENSG00000100916 | NONHSAT036397.2 | 0.232035 | 0.0889367 | 0.07649159 | 0.023338302 |
| ENSG00000263945 | NONHSAT036688.2 | 0.06446032 | 0.00720881 | 0.594970411 | 0.05673413 |
| ENSG00000100614 | NONHSAT037217.2 | 13.7413134 | 0.21531798 | 6.63228898 | 0.23473515 |
| ENSG00000228966 | NONHSAT040509.2 | 0.16680191 | 0.07461601 | 2.155326043 | 0.352365293 |
| ENSG00000166145 | NONHSAT041891.2 | 0.84535659 | 0.15126238 | 2.496788479 | 0.238185648 |
| ENSG00000247556 | NONHSAT041921.2 | 0.02825223 | 0.01010572 | 0.093870383 | 0.00796028 |
| ENSG00000171914 | NONHSAT046579.2 | 0.87546519 | 0.17397262 | 0.359172806 | 0.137038318 |
| ENSG00000103942 | NONHSAT047869.2 | 1.61117227 | 0.65596693 | 0.710262035 | 0.220124676 |
| ENSG00000140563 | NONHSAT050160.2 | 4.68266018 | 0.11318978 | 0.175212765 | 0.044552239 |
| ENSG00000168904 | NONHSAT050868.2 | 2.69139967 | 1.21878788 | 0.628919203 | 0.14394086 |
| ENSG00000167543 | NONHSAT052714.2 | 0.15389154 | 0.06884077 | 0.710211391 | 0.216692158 |
| ENSG00000264538 | NONHSAT052822.2 | 0.36582865 | 0.16356947 | 0.759685155 | 0.321980542 |
| ENSG00000092871 | NONHSAT053044.2 | 0.57018789 | 0.20996705 | 0.123844335 | 0.047251404 |
| ENSG00000278845 | NONHSAT053302.2 | 0.57244109 | 0.08535728 | 0.352278895 | 0.134408024 |
| ENSG00000273604 | NONHSAT053347.2 | 3.02392326 | 0.16902749 | 0.627967881 | 0.266154258 |
| ENSG00000004897 | NONHSAT054285.2 | 0.50209058 | 0.22449497 | 0.46347804 | 0.176834799 |
| ENSG00000228782 | NONHSAT054309.2 | 1.29896257 | 0.46470783 | 1.27884288 | 0.609743639 |
| ENSG00000121057 | NONHSAT054880.2 | 0.15090154 | 0.0270013 | 0.222874686 | 0.042517657 |
| ENSG00000108370 | NONHSAT055362.2 | 0.39765574 | 0.11858971 | 0.978865622 | 0.186737548 |
| ENSG00000154217 | NONHSAT055431.2 | 0.11322772 | 0.0202602 | 0.083616084 | 0.03190277 |
| ENSG00000129673 | NONHSAT056013.2 | 0.8533385 | 0.19086326 | 0.787713751 | 0.300543264 |
| ENSG00000185624 | NONHSAT056570.2 | 0.05426525 | 0.02427464 | 0.601053723 | 0.076410008 |
| ENSG00000172270 | NONHSAT060142.2 | 0.39387455 | 0.07047724 | 0.436227028 | 0.110977148 |
| ENSG00000223573 | NONHSAT060692.2 | 0.40172657 | 0.14371891 | 1.43370905 | 0.339455358 |
| ENSG00000266983 | NONHSAT060732.2 | 0.45065247 | 0.02015918 | 0.415953362 | 0.03174369 |
| ENSG00000127445 | NONHSAT061010.2 | 0.23179415 | 0.09213531 | 0.118859212 | 0.054406662 |
| ENSG00000198551 | NONHSAT061179.2 | 0.46746365 | 0.1393417 | 0.862881675 | 0.10975953 |
| ENSG00000095066 | NONHSAT061300.2 | 1.37381067 | 0.67020677 | 1.152755172 | 0.351716418 |
| ENSG00000272635 | NONHSAT063995.2 | 0.09044678 | 0.03466887 | 0.071551665 | 0.018202906 |
| ENSG00000213965 | NONHSAT064215.2 | 0.14915819 | 0.0333617 | 0.757175594 | 0.367626414 |
| ENSG00000186017 | NONHSAT066006.2 | 0.04918236 | 0.01466726 | 0.151318126 | 0.069264418 |
| ENSG00000142546 | NONHSAT067214.2 | 0.57134446 | 0.17038751 | 1.054633159 | 0.268301074 |
| ENSG00000189292 | NONHSAT068503.2 | 0.3794425 | 0.18852515 | 0.700405228 | 0.059394894 |
| ENSG00000255767 | NONHSAT068729.2 | 0.47004225 | 0.2101655 | 0.867788672 | 0.165547471 |
| ENSG00000115758 | NONHSAT069104.2 | 0.53846411 | 0.18059736 | 0.496991072 | 0.142186672 |
| ENSG00000266738 | NONHSAT069391.2 | 1.72476315 | 0.41134399 | 1.63745763 | 0.323867149 |
| ENSG00000213639 | NONHSAT069847.2 | 0.13080781 | 0.05541737 | 0.050841358 | 0.009698972 |
| ENSG00000230979 | NONHSAT070592.2 | 0.80172529 | 0.35846777 | 2.219832697 | 0.564448122 |
| ENSG00000162869 | NONHSAT070622.2 | 1.69011457 | 0.06873437 | 1.418227383 | 0.649179952 |
| ENSG00000237651 | NONHSAT070938.2 | 0.17022934 | 0.05076614 | 0.20951747 | 0.079939019 |
| ENSG00000144043 | NONHSAT071529.2 | 0.28520391 | 0.10932061 | 0.150440413 | 0.057398836 |
| ENSG00000187605 | NONHSAT071649.2 | 0.73098482 | 0.14526145 | 1.649207626 | 0.57188374 |
| ENSG00000179528 | NONHSAT071727.2 | 0.10148421 | 0.0181589 | 0.074943775 | 0.028593949 |
| ENSG00000222041 | NONHSAT072146.2 | 0.18501353 | 0.05517511 | 1.479965721 | 0.521115917 |
| ENSG00000125611 | NONHSAT073700.2 | 2.9887523 | 0.35004542 | 1.510698274 | 0.150329073 |
| ENSG00000233654 | NONHSAT076089.2 | 0.45047226 | 0.19652622 | 0.547618632 | 0.185684982 |
| ENSG00000173559 | NONHSAT076128.2 | 0.07366724 | 0.01646906 | 0.152978467 | 0.064837504 |
| ENSG00000101365 | NONHSAT078198.2 | 0.01653592 | 0.00739706 | 0.152626932 | 0.034931756 |
| ENSG00000101294 | NONHSAT079300.2 | 8.03627922 | 2.12959463 | 3.296741738 | 1.257543201 |
| ENSG00000132823 | NONHSAT079851.2 | 2.33742059 | 0.66127547 | 2.99401205 | 0.100772416 |
| ENSG00000159267 | NONHSAT081997.2 | 0.33929671 | 0.03794466 | 0.156601795 | 0.059749642 |
| ENSG00000160194 | NONHSAT082357.2 | 1.0466662 | 0.0382037 | 0.512613825 | 0.240654303 |
| ENSG00000160305 | NONHSAT083070.2 | 0.28262668 | 0.03160706 | 0.847798416 | 0.248751136 |
| ENSG00000261188 | NONHSAT084496.2 | 0.3978395 | 0.00547607 | 0.135590496 | 0.008622901 |
| ENSG00000180957 | NONHSAT084621.2 | 0.28557569 | 0.04507305 | 0.930307317 | 0.023665624 |
| ENSG00000225733 | NONHSAT088373.2 | 19.2396976 | 0.82604281 | 10.90243508 | 0.512966235 |
| ENSG00000164048 | NONHSAT089548.2 | 0.41217413 | 0.09556854 | 0.197276985 | 0.064499298 |
| ENSG00000272305 | NONHSAT089980.2 | 0.06038995 | 0.02700415 | 0.267545552 | 0.110557091 |
| ENSG00000157445 | NONHSAT090017.2 | 1.288448 | 0.60646973 | 2.12814049 | 0.668548444 |
| ENSG00000163681 | NONHSAT090097.2 | 0.22731845 | 0.09034554 | 0.233128333 | 0.071129641 |
| ENSG00000244026 | NONHSAT090526.2 | 0.2972016 | 0.13288491 | 0.685794557 | 0.156957933 |
| ENSG00000239213 | NONHSAT092288.2 | 0.76596301 | 0.108168 | 1.041828323 | 0.510972542 |
| ENSG00000163864 | NONHSAT092379.2 | 0.40761001 | 0.01919341 | 0.118799851 | 0.030222952 |
| ENSG00000155903 | NONHSAT092438.2 | 1.0606127 | 0.01824831 | 0.640101346 | 0.14361627 |
| ENSG00000196428 | NONHSAT092691.2 | 1.24610525 | 0.01376389 | 1.675562782 | 0.021673346 |
| ENSG00000215375 | NONHSAT094599.2 | 1.79007575 | 0.73222362 | 1.178361523 | 0.586272228 |
| ENSG00000163945 | NONHSAT094698.2 | 0.16486556 | 0.07371477 | 0.380428324 | 0.116072251 |
| ENSG00000109689 | NONHSAT095887.2 | 1.15095729 | 0.38602358 | 1.460658564 | 0.101340927 |
| ENSG00000084093 | NONHSAT096532.2 | 0.35504977 | 0.04537871 | 0.187282963 | 0.071455694 |
| ENSG00000248049 | NONHSAT096697.2 | 0.12123651 | 0.0542331 | 0.223825981 | 0.085398269 |
| ENSG00000174796 | NONHSAT096929.2 | 0.63561802 | 0.28423421 | 0.351993932 | 0.059668855 |
| ENSG00000151247 | NONHSAT097499.2 | 0.04887607 | 0.01457592 | 0.060156442 | 0.022952009 |
| ENSG00000196782 | NONHSAT098487.2 | 0.11538293 | 0.04127206 | 0.127789812 | 0.032510019 |
| ENSG00000245213 | NONHSAT099223.2 | 0.29379206 | 0.12043183 | 0.757024894 | 0.301698066 |
| ENSG00000145495 | NONHSAT100442.2 | 0.05444356 | 0.01623627 | 0.067008873 | 0.025566477 |
| ENSG00000112977 | NONHSAT100484.2 | 0.40086264 | 0.17931911 | 0.740069774 | 0.282365244 |
| ENSG00000183666 | NONHSAT100712.2 | 0.24129596 | 0.10788836 | 0.55679194 | 0.169924936 |
| ENSG00000213949 | NONHSAT101419.2 | 0.15489058 | 0.03463286 | 0.047659648 | 0.018183999 |
| ENSG00000253366 | NONHSAT101985.2 | 3.42236636 | 1.60332279 | 2.256376517 | 0.344278221 |
| ENSG00000164300 | NONHSAT102392.2 | 0.33510315 | 0.09993515 | 0.41244338 | 0.157363102 |
| ENSG00000175745 | NONHSAT102816.2 | 0.50124673 | 0.14941178 | 0.308466057 | 0.117691732 |
| ENSG00000250682 | NONHSAT103034.2 | 2.24303505 | 0.16049476 | 1.048959615 | 0.084225713 |
| ENSG00000248663 | NONHSAT103388.2 | 0.03198647 | 0.0143086 | 0.177129507 | 0.067570693 |
| ENSG00000274553 | NONHSAT105953.2 | 0.04549837 | 0.01017646 | 0.041999383 | 0.016024389 |
| ENSG00000229931 | NONHSAT107910.2 | 0.04705317 | 0.01683075 | 0.138973081 | 0.039759507 |
| ENSG00000219891 | NONHSAT108425.2 | 0.17161469 | 0.02192355 | 0.181024861 | 0.017269194 |
| ENSG00000204315 | NONHSAT108879.2 | 0.71349714 | 0.31906973 | 0.878168919 | 0.334887881 |
| ENSG00000268745 | NONHSAT112767.2 | 0.69227804 | 0.10614496 | 1.131914565 | 0.13927292 |
| ENSG00000118407 | NONHSAT113605.2 | 0.16999647 | 0.06080712 | 0.125538512 | 0.047897798 |
| ENSG00000276064 | NONHSAT113650.2 | 0.06887006 | 0.03079321 | 0.127147422 | 0.048487404 |
| ENSG00000135549 | NONHSAT114757.2 | 0.42412089 | 0.1517065 | 1.879062152 | 0.836255989 |
| ENSG00000225177 | NONHSAT115213.2 | 1.27596747 | 0.51871915 | 0.428325097 | 0.081711296 |
| ENSG00000205903 | NONHSAT119053.2 | 0.41184868 | 0.02834362 | 1.695961757 | 0.803096423 |
| ENSG00000196295 | NONHSAT119914.2 | 0.19803573 | 0.05905862 | 0.24374144 | 0.092996787 |
| ENSG00000277184 | NONHSAT120412.2 | 0.65561111 | 0.26230943 | 0.509584042 | 0.194377842 |
| ENSG00000273024 | NONHSAT121096.2 | 0.24978376 | 0.0638191 | 0.395203524 | 0.100490405 |
| ENSG00000275833 | NONHSAT121114.2 | 0.22594076 | 0.10107077 | 0.417130236 | 0.159151319 |
| ENSG00000196313 | NONHSAT121375.2 | 0.88271023 | 0.36023839 | 0.221478331 | 0.036208571 |
| ENSG00000066923 | NONHSAT122348.2 | 0.32871812 | 0.08574571 | 0.177000238 | 0.038573473 |
| ENSG00000157741 | NONHSAT123630.2 | 0.29764651 | 0.13314713 | 0.824129504 | 0.20966042 |
| ENSG00000013374 | NONHSAT124207.2 | 4.09552107 | 0.16655858 | 0.687405633 | 0.262271837 |
| ENSG00000253279 | NONHSAT125838.2 | 0.05250736 | 0.01565885 | 0.193844545 | 0.073947083 |
| ENSG00000175073 | NONHSAT127038.2 | 0.98538042 | 0.19581499 | 0.40426719 | 0.154243569 |
| ENSG00000249395 | NONHSAT127311.2 | 0.70113763 | 0.13199704 | 9.536835064 | 1.195286664 |
| ENSG00000251136 | NONHSAT127624.2 | 0.067445 | 0.03017038 | 0.249032977 | 0.094968205 |
| ENSG00000156162 | NONHSAT127798.2 | 2.96547251 | 0.12633847 | 2.606798628 | 0.198939149 |
| ENSG00000155096 | NONHSAT128126.2 | 0.1325048 | 0.023702 | 0.130452428 | 0.024877045 |
| ENSG00000253210 | NONHSAT129402.2 | 0.9120294 | 0.29455324 | 0.654726973 | 0.285424344 |
| ENSG00000181404 | NONHSAT129879.2 | 0.80316465 | 0.07984047 | 1.153202626 | 0.50275816 |
| ENSG00000080608 | NONHSAT130008.2 | 0.53583922 | 0.14094556 | 0.232767665 | 0.088765462 |
| ENSG00000080298 | NONHSAT130021.2 | 0.33137979 | 0.14819016 | 1.325391524 | 0.077807312 |
| ENSG00000165121 | NONHSAT132814.2 | 0.22075633 | 0.06580311 | 0.339597749 | 0.103614459 |
| ENSG00000165152 | NONHSAT133739.2 | 1.0287964 | 0.02629872 | 0.16277898 | 0.041411343 |
| ENSG00000157657 | NONHSAT134364.2 | 0.86626251 | 0.18445739 | 0.152313115 | 0.058113345 |
| ENSG00000157693 | NONHSAT134388.2 | 0.08607267 | 0.03848485 | 0.238319701 | 0.060598759 |
| ENSG00000235106 | NONHSAT135354.2 | 1.6262579 | 0.00989787 | 1.225342822 | 0.046741535 |
| ENSG00000148362 | NONHSAT135642.2 | 0.32701952 | 0.11697369 | 0.241496453 | 0.092140237 |
| ENSG00000204272 | NONHSAT137273.2 | 0.30401815 | 0.13593273 | 0.42088571 | 0.107074282 |
| ENSG00000235437 | NONHSAT137306.2 | 0.22013715 | 0.07874226 | 0.568940534 | 0.062025315 |
| ENSG00000102385 | NONHSAT137913.2 | 0.12978321 | 0.03315927 | 0.102670367 | 0.026119575 |
| ENSG00000203950 | NONHSAT138672.2 | 0.73314112 | 0.1311833 | 2.435922606 | 0.619497683 |
| ENSG00000169057 | NONHSAT139137.2 | 0.17091221 | 0.05094556 | 0.105178971 | 0.040129845 |
| ENSG00000102125 | NONHSAT139163.2 | 1.27394244 | 0.51970635 | 0.660122134 | 0.220344429 |
| ENSG00000071553 | NONHSAT139171.2 | 0.41353551 | 0.03699762 | 0.458002087 | 0.174716901 |
| ENSG00000182484 | NONHSAT139258.2 | 0.12101514 | 0.05410834 | 0.167534543 | 0.042621169 |
| ENSG00000237296 | NONHSAT141068.2 | 0.43794685 | 0.19584603 | 0.539022919 | 0.102829046 |
| ENSG00000166851 | NONHSAT141144.2 | 0.63053236 | 0.2819686 | 0.969971582 | 0.29594743 |
| ENSG00000260083 | NONHSAT141823.2 | 0.88364821 | 0.02823564 | 0.640834963 | 0.133339501 |
| ENSG00000245694 | NONHSAT142619.2 | 0.12959735 | 0.05381446 | 0.07689522 | 0.013035025 |
| ENSG00000166446 | NONHSAT143903.2 | 0.15754982 | 0.05284118 | 0.218113514 | 0.05546083 |
| ENSG00000221819 | NONHSAT144638.2 | 0.71783196 | 0.09986238 | 0.47115264 | 0.157267654 |
| ENSG00000011295 | NONHSAT145886.2 | 0.58709017 | 0.08754161 | 0.722587811 | 0.275557351 |
| ENSG00000108474 | NONHSAT145903.2 | 1.17690592 | 0.55726106 | 0.76673887 | 0.292492639 |
| ENSG00000175061 | NONHSAT145955.2 | 0.4602497 | 0.13725661 | 0.566473161 | 0.216131421 |
| ENSG00000072310 | NONHSAT146109.2 | 2.88659563 | 1.13340597 | 2.729331706 | 0.396651976 |
| ENSG00000171962 | NONHSAT146130.2 | 0.43664643 | 0.17899467 | 0.839671593 | 0.256217312 |
| ENSG00000176974 | NONHSAT146178.2 | 3.02643366 | 0.10755738 | 5.623890021 | 0.169362673 |
| ENSG00000234546 | NONHSAT148341.1 | 0.27279169 | 0.11182562 | 0.188835528 | 0.016013396 |
| ENSG00000048707 | NONHSAT148397.1 | 0.79440359 | 0.20296805 | 0.628445786 | 0.159878037 |
| ENSG00000117676 | NONHSAT148570.1 | 2.24918406 | 0.80452496 | 1.245516988 | 0.316862355 |
| ENSG00000127603 | NONHSAT148776.1 | 0.43794685 | 0.19590811 | 0.808534379 | 0.308487138 |
| ENSG00000171793 | NONHSAT148805.1 | 1.08395257 | 0.13853938 | 0.571767302 | 0.218151341 |
| ENSG00000197147 | NONHSAT149322.1 | 0.40632399 | 0.04844687 | 0.550035459 | 0.228893422 |
| ENSG00000117569 | NONHSAT149428.1 | 0.63649507 | 0.06619153 | 0.109313463 | 0.04168646 |
| ENSG00000231551 | NONHSAT149749.1 | 0.0635639 | 0.02843423 | 0.704047584 | 0.134277311 |
| ENSG00000227232 | NONHSAT150910.1 | 1.11650615 | 0.44240556 | 2.843791734 | 1.114611308 |
| ENSG00000142627 | NONHSAT151176.1 | 0.42352955 | 0.20661681 | 0.142166773 | 0.054242123 |
| ENSG00000162542 | NONHSAT151219.1 | 0.11440039 | 0.02558754 | 0.263979608 | 0.080542655 |
| ENSG00000184007 | NONHSAT151376.1 | 2.66554305 | 0.59030997 | 11.20032311 | 0.929550996 |
| ENSG00000081870 | NONHSAT151617.1 | 11.1525353 | 1.63376355 | 10.64886905 | 2.437250073 |
| ENSG00000031698 | NONHSAT152058.1 | 0.40086264 | 0.11954608 | 0.49337985 | 0.188243496 |
| ENSG00000223945 | NONHSAT152283.1 | 0.41858499 | 0.14039068 | 0.965887492 | 0.368413525 |
| ENSG00000231966 | NONHSAT152599.1 | 0.36549241 | 0.10899789 | 0.899692667 | 0.171633775 |
| ENSG00000054392 | NONHSAT152846.1 | 0.23698197 | 0.05300493 | 0.437514432 | 0.083464339 |
| ENSG00000272645 | NONHSAT153028.1 | 0.65837043 | 0.2453324 | 0.202579806 | 0.077292031 |
| ENSG00000143643 | NONHSAT153106.1 | 0.3471157 | 0.06211053 | 0.256337017 | 0.097802487 |
| ENSG00000095787 | NONHSAT155266.1 | 0.21533704 | 0.03210913 | 0.132517909 | 0.050560708 |
| ENSG00000165874 | NONHSAT155461.1 | 0.19416784 | 0.0868577 | 0.358471297 | 0.136770665 |
| ENSG00000148634 | NONHSAT155594.1 | 0.42303802 | 0.09457448 | 0.292828994 | 0.074496362 |
| ENSG00000156113 | NONHSAT155717.1 | 0.032995 | 0.01475975 | 0.060915136 | 0.02324148 |
| ENSG00000119969 | NONHSAT155898.1 | 1.43510931 | 0.58338886 | 1.68591565 | 0.183796799 |
| ENSG00000249456 | NONHSAT156305.1 | 0.23984062 | 0.10723765 | 0.553433726 | 0.168857822 |
| ENSG00000197321 | NONHSAT156818.1 | 0.46746365 | 0.20904569 | 1.294322513 | 0.438928362 |
| ENSG00000119977 | NONHSAT157359.1 | 10.4864255 | 0.43765283 | 4.774992701 | 0.787626671 |
| ENSG00000165752 | NONHSAT157762.1 | 0.21151901 | 0.06307963 | 0.260336601 | 0.099328483 |
| ENSG00000166833 | NONHSAT158908.1 | 0.57631267 | 0.25771181 | 0.265996093 | 0.101437054 |
| ENSG00000276639 | NONHSAT159095.1 | 0.02067248 | 0.00924309 | 0.066784556 | 0.014554287 |
| ENSG00000174684 | NONHSAT159267.1 | 0.78959631 | 0.16813249 | 0.277666118 | 0.105887394 |
| ENSG00000023445 | NONHSAT159591.1 | 0.25754906 | 0.11515545 | 1.901698997 | 0.090708006 |
| ENSG00000118058 | NONHSAT159744.1 | 0.10001402 | 0.03577465 | 0.369252763 | 0.084510952 |
| ENSG00000110002 | NONHSAT159803.1 | 2.14074536 | 0.53851069 | 2.59341613 | 0.565302058 |
| ENSG00000166801 | NONHSAT160347.1 | 0.53448352 | 0.13655915 | 0.49334398 | 0.21508164 |
| ENSG00000149016 | NONHSAT160383.1 | 0.57134446 | 0.17038751 | 0.703208061 | 0.268301074 |
| ENSG00000174996 | NONHSAT160442.1 | 0.26161562 | 0.06684211 | 0.275995946 | 0.105250477 |
| ENSG00000175482 | NONHSAT160452.1 | 0.59087034 | 0.13209511 | 4.090311071 | 0.728151574 |
| ENSG00000137501 | NONHSAT160586.1 | 0.15957293 | 0.07138225 | 0.589204828 | 0.112402216 |
| ENSG00000254750 | NONHSAT160690.1 | 0.65669836 | 0.30378746 | 0.6270568 | 0.255126453 |
| ENSG00000065361 | NONHSAT162253.1 | 0.12334235 | 0.05517511 | 0.341512723 | 0.086881613 |
| ENSG00000229117 | NONHSAT162255.1 | 0.32347268 | 0.09646668 | 2.189403617 | 0.30365105 |
| ENSG00000135439 | NONHSAT162285.1 | 1.10384568 | 0.02904712 | 0.958923335 | 0.457208163 |
| ENSG00000127311 | NONHSAT162353.1 | 0.19266267 | 0.05745625 | 0.2371283 | 0.090473618 |
| ENSG00000110911 | NONHSAT163536.1 | 2.55164191 | 1.18668062 | 3.203039585 | 1.437414191 |
| ENSG00000197111 | NONHSAT163571.1 | 0.39765574 | 0.17788456 | 0.734149216 | 0.280106322 |
| ENSG00000135473 | NONHSAT163609.1 | 3.54607007 | 1.36698128 | 1.692976231 | 0.344448087 |
| ENSG00000256022 | NONHSAT164213.1 | 0.11492941 | 0.02570586 | 0.106090927 | 0.040477792 |
| ENSG00000250208 | NONHSAT164245.1 | 0.23480256 | 0.06517567 | 0.029896938 | 0.011406838 |
| ENSG00000136104 | NONHSAT165715.1 | 0.54206072 | 0.04849634 | 0.200149732 | 0.076364864 |
| ENSG00000134871 | NONHSAT166321.1 | 0.15389154 | 0.03442039 | 2.343683131 | 0.162546219 |
| ENSG00000122033 | NONHSAT166505.1 | 0.29794386 | 0.0888112 | 0.45833822 | 0.069956624 |
| ENSG00000197324 | NONHSAT168169.1 | 0.71474071 | 0.17434137 | 2.518834228 | 1.006551997 |
| ENSG00000100897 | NONHSAT168192.1 | 0.09941394 | 0.04447114 | 0.275259254 | 0.07002658 |
| ENSG00000151327 | NONHSAT168288.1 | 0.91741048 | 0.37293818 | 1.38553316 | 0.117494262 |
| ENSG00000131981 | NONHSAT168465.1 | 4.99117758 | 0.87438484 | 5.414291971 | 1.159425597 |
| ENSG00000100644 | NONHSAT168541.1 | 120.181634 | 5.57276978 | 49.58197296 | 16.26837313 |
| ENSG00000151445 | NONHSAT169524.1 | 2.39377355 | 0.04199352 | 0.259923753 | 0.066125194 |
| ENSG00000128918 | NONHSAT170855.1 | 0.33206094 | 0.06853605 | 0.141448743 | 0.035984882 |
| ENSG00000283273 | NONHSAT171363.1 | 0.02395517 | 0.01071594 | 0.044225856 | 0.016873875 |
| ENSG00000227161 | NONHSAT171633.1 | 14.9983507 | 0.04844351 | 21.69009707 | 0.610024579 |
| ENSG00000176463 | NONHSAT172414.1 | 0.41819289 | 0.10686367 | 0.193008973 | 0.021041641 |
| ENSG00000059122 | NONHSAT172568.1 | 0.51571475 | 0.17739119 | 0.439360342 | 0.167605518 |
| ENSG00000166783 | NONHSAT172705.1 | 0.23669985 | 0.10588367 | 0.873987162 | 0.333293177 |
| ENSG00000140941 | NONHSAT173430.1 | 0.74189504 | 0.22124945 | 0.913120916 | 0.348390947 |
| ENSG00000140632 | NONHSAT173563.1 | 1.79825842 | 0.20618846 | 2.042826938 | 0.194814207 |
| ENSG00000140743 | NONHSAT173692.1 | 0.53269356 | 0.17866195 | 0.307298432 | 0.093759634 |
| ENSG00000103121 | NONHSAT174097.1 | 1.54723484 | 0.7495635 | 0.952082766 | 0.453946626 |
| ENSG00000103264 | NONHSAT174189.1 | 1.49801821 | 0.60896207 | 0.502841929 | 0.191853645 |
| ENSG00000146872 | NONHSAT175457.1 | 0.28242595 | 0.08418561 | 0.173804265 | 0.06631305 |
| ENSG00000266173 | NONHSAT175466.1 | 0.47988563 | 0.21459839 | 0.511099025 | 0.181970297 |
| ENSG00000108984 | NONHSAT175547.1 | 2.20914394 | 0.14819016 | 0.101965169 | 0.038903656 |
| ENSG00000185298 | NONHSAT175752.1 | 0.51465368 | 0.23014866 | 1.108347128 | 0.483177363 |
| ENSG00000108599 | NONHSAT176036.1 | 0.1684982 | 0.07537481 | 0.311080176 | 0.118689119 |
| ENSG00000070540 | NONHSAT176486.1 | 1.44893675 | 0.39438677 | 3.721448988 | 0.621097604 |
| ENSG00000125458 | NONHSAT176567.1 | 1.07539113 | 0.32060372 | 0.882278621 | 0.420664482 |
| ENSG00000075336 | NONHSAT178001.1 | 6.19921862 | 0.16699561 | 3.033323571 | 0.105171401 |
| ENSG00000130816 | NONHSAT179438.1 | 0.17395264 | 0.05185186 | 0.267597876 | 0.061245178 |
| ENSG00000178386 | NONHSAT179785.1 | 0.23783238 | 0.10633972 | 0.548799711 | 0.251207793 |
| ENSG00000104852 | NONHSAT179872.1 | 1.0286631 | 0.47840518 | 1.443187815 | 0.289729179 |
| ENSG00000160439 | NONHSAT179953.1 | 0.10128776 | 0.02265468 | 0.186996744 | 0.035673245 |
| ENSG00000129990 | NONHSAT179957.1 | 0.2475095 | 0.07115658 | 0.24477853 | 0.024894742 |
| ENSG00000134330 | NONHSAT181236.1 | 1.23444456 | 0.58881176 | 5.317178658 | 1.738491015 |
| ENSG00000138085 | NONHSAT181468.1 | 1.77046264 | 0.43545276 | 0.898766756 | 0.436372226 |
| ENSG00000173209 | NONHSAT181876.1 | 1.54232335 | 0.38719902 | 1.048942942 | 0.076202033 |
| ENSG00000170417 | NONHSAT182284.1 | 0.17174174 | 0.06582979 | 0.181181939 | 0.069093354 |
| ENSG00000153250 | NONHSAT182981.1 | 1.58808204 | 0.4261059 | 0.586381163 | 0.223727094 |
| ENSG00000170035 | NONHSAT183229.1 | 0.14081294 | 0.02519611 | 0.259941393 | 0.079310558 |
| ENSG00000220804 | NONHSAT183953.1 | 0.68936671 | 0.06489052 | 0.200919164 | 0.051114292 |
| ENSG00000115875 | NONHSAT184507.1 | 1.94459591 | 0.72110064 | 2.188914878 | 0.801563796 |
| ENSG00000198648 | NONHSAT185790.1 | 0.4611036 | 0.13745897 | 0.227009652 | 0.086569656 |
| ENSG00000128641 | NONHSAT186103.1 | 0.4475004 | 0.2154925 | 0.444829545 | 0.193930952 |
| ENSG00000074582 | NONHSAT186319.1 | 0.72388788 | 0.21579724 | 1.425351093 | 0.203892927 |
| ENSG00000135919 | NONHSAT186356.1 | 1.6093383 | 0.20561624 | 4.583669556 | 2.266503233 |
| ENSG00000198900 | NONHSAT188755.1 | 0.06518947 | 0.0291614 | 0.601700405 | 0.045919069 |
| ENSG00000125835 | NONHSAT189219.1 | 1.55643115 | 0.49395298 | 2.13176312 | 0.494995969 |
| ENSG00000101311 | NONHSAT189278.1 | 0.15460954 | 0.06916196 | 0.285439042 | 0.108906035 |
| ENSG00000089006 | NONHSAT189393.1 | 6.21959238 | 3.05483826 | 6.870069405 | 0.143572445 |
| ENSG00000125844 | NONHSAT190140.1 | 0.44099971 | 0.05636393 | 0.465240314 | 0.177418421 |
| ENSG00000154642 | NONHSAT191261.1 | 0.31002267 | 0.09245559 | 0.572264562 | 0.145585406 |
| ENSG00000214889 | NONHSAT191442.1 | 0.42862601 | 0.08372046 | 2.091818959 | 0.832614583 |
| ENSG00000273199 | NONHSAT191444.1 | 0.3948915 | 0.13244404 | 0.182261474 | 0.069539802 |
| ENSG00000160208 | NONHSAT191558.1 | 1.87991683 | 0.20177178 | 0.416428524 | 0.105940364 |
| ENSG00000100426 | NONHSAT192565.1 | 1.32669843 | 0.23731521 | 1.632727966 | 0.747365532 |
| ENSG00000100330 | NONHSAT192798.1 | 0.18026099 | 0.05373225 | 0.138651121 | 0.063466218 |
| ENSG00000133466 | NONHSAT192897.1 | 2.74917042 | 0.59013782 | 0.608980651 | 0.154926063 |
| ENSG00000151090 | NONHSAT193640.1 | 0.69574294 | 0.34567831 | 0.856171865 | 0.326609198 |
| ENSG00000170248 | NONHSAT193719.1 | 0.50721396 | 0.22678573 | 0.468207408 | 0.178639236 |
| ENSG00000114353 | NONHSAT193871.1 | 2.21900619 | 1.09154788 | 4.096397844 | 0.468771684 |
| ENSG00000114738 | NONHSAT193884.1 | 9.55879589 | 3.93864248 | 9.264045806 | 3.028938173 |
| ENSG00000163635 | NONHSAT193972.1 | 0.78782718 | 0.19217745 | 1.189948797 | 0.252156231 |
| ENSG00000036054 | NONHSAT194224.1 | 10.3465468 | 0.9485456 | 15.31480454 | 6.804365608 |
| ENSG00000197980 | NONHSAT194729.1 | 4.45137025 | 0.22124945 | 2.282569943 | 0.696433504 |
| ENSG00000178567 | NONHSAT195577.1 | 0.758885 | 0.29088582 | 2.10131754 | 0.763343182 |
| ENSG00000163808 | NONHSAT195697.1 | 0.13115295 | 0.05866905 | 0.363138858 | 0.092383351 |
| ENSG00000181555 | NONHSAT195721.1 | 1.65002383 | 0.7377594 | 1.90372016 | 0.580842881 |
| ENSG00000068745 | NONHSAT195730.1 | 0.20603924 | 0.04608408 | 0.285242751 | 0.072566405 |
| ENSG00000168291 | NONHSAT195819.1 | 0.86515292 | 0.2110303 | 0.290407127 | 0.110801551 |
| ENSG00000114541 | NONHSAT195866.1 | 0.65837043 | 0.2453324 | 0.506397967 | 0.077292031 |
| ENSG00000181722 | NONHSAT196117.1 | 0.51571475 | 0.17739119 | 0.439360342 | 0.055887135 |
| ENSG00000197763 | NONHSAT196201.1 | 5.96468087 | 2.19935019 | 2.028324405 | 0.957889908 |
| ENSG00000174891 | NONHSAT196488.1 | 0.35280971 | 0.15777344 | 0.072372754 | 0.027613005 |
| ENSG00000114770 | NONHSAT196662.1 | 0.32120819 | 0.03592176 | 0.222341886 | 0.056564281 |
| ENSG00000136527 | NONHSAT196677.1 | 3.55447197 | 1.11013269 | 1.874743394 | 0.874020747 |
| ENSG00000133657 | NONHSAT196824.1 | 27.0195675 | 3.64755918 | 23.29248491 | 2.692342461 |
| ENSG00000013288 | NONHSAT198237.1 | 1.05833546 | 0.22846635 | 1.95371806 | 0.71954625 |
| ENSG00000163110 | NONHSAT198836.1 | 0.25543149 | 0.04570518 | 0.660158132 | 0.071969764 |
| ENSG00000071205 | NONHSAT199202.1 | 0.09486063 | 0.04243429 | 0.525303921 | 0.066819256 |
| ENSG00000109686 | NONHSAT200578.1 | 0.37233684 | 0.08327929 | 0.515466769 | 0.131135918 |
| ENSG00000145439 | NONHSAT200667.1 | 1.9959415 | 0.86374801 | 2.020630802 | 0.725391922 |
| ENSG00000174473 | NONHSAT200687.1 | 0.22215405 | 0.04968842 | 0.410139227 | 0.078241989 |
| ENSG00000131844 | NONHSAT202728.1 | 0.19922632 | 0.08907816 | 4.826971105 | 0.245514033 |
| ENSG00000120733 | NONHSAT203274.1 | 1.16740715 | 0.21977752 | 0.567115647 | 0.259591736 |
| ENSG00000131504 | NONHSAT203329.1 | 0.59059672 | 0.05111964 | 0.492362714 | 0.187829057 |
| ENSG00000113645 | NONHSAT203532.1 | 0.67475522 | 0.20122688 | 1.660971077 | 0.633407847 |
| ENSG00000145757 | NONHSAT204478.1 | 1.43504478 | 0.52508326 | 0.602095221 | 0.183704901 |
| ENSG00000174136 | NONHSAT204498.1 | 0.09799304 | 0.04381469 | 0.18091405 | 0.034512854 |
| ENSG00000153922 | NONHSAT204503.1 | 1.01250382 | 0.32929843 | 0.934550988 | 0.324067453 |
| ENSG00000161021 | NONHSAT205113.1 | 0.57332143 | 0.20509937 | 0.58207328 | 0.282610155 |
| ENSG00000248109 | NONHSAT206419.1 | 0.12688441 | 0.0283798 | 1.054019738 | 0.044688309 |
| ENSG00000137210 | NONHSAT206797.1 | 0.80645523 | 0.06559165 | 0.947395077 | 0.413032883 |
| ENSG00000111801 | NONHSAT206989.1 | 0.4791033 | 0.16068813 | 0.221129282 | 0.084369374 |
| ENSG00000196586 | NONHSAT207507.1 | 7.42135572 | 0.03088274 | 0.127456461 | 0.04862957 |
| ENSG00000233967 | NONHSAT207533.1 | 0.13243419 | 0.0394948 | 0.162999382 | 0.062190569 |
| ENSG00000152818 | NONHSAT208189.1 | 1.11037302 | 0.55496436 | 3.376139352 | 1.379781287 |
| ENSG00000055211 | NONHSAT208243.1 | 2.22261932 | 0.40661935 | 1.585291646 | 0.569108296 |
| ENSG00000184465 | NONHSAT208537.1 | 0.69735061 | 0.31184124 | 0.178798347 | 0.081843225 |
| ENSG00000111843 | NONHSAT208742.1 | 0.35353462 | 0.06323905 | 0.261032958 | 0.066374175 |
| ENSG00000137364 | NONHSAT208823.1 | 0.14525707 | 0.02599132 | 0.536290542 | 0.040927283 |
| ENSG00000137337 | NONHSAT208960.1 | 1.01789012 | 0.35400587 | 1.565858561 | 0.318585091 |
| ENSG00000180316 | NONHSAT209047.1 | 0.15108501 | 0.05067293 | 0.069733018 | 0.026605844 |
| ENSG00000137216 | NONHSAT209147.1 | 0.87644528 | 0.33594748 | 0.924503543 | 0.176301177 |
| ENSG00000146247 | NONHSAT209344.1 | 1.37756031 | 0.30801651 | 0.423873681 | 0.161724204 |
| ENSG00000132424 | NONHSAT209476.1 | 0.72213512 | 0.21525426 | 0.999730948 | 0.169471107 |
| ENSG00000111897 | NONHSAT209637.1 | 0.28983655 | 0.08639457 | 0.267501705 | 0.068053042 |
| ENSG00000026652 | NONHSAT209970.1 | 0.16764576 | 0.07499349 | 0.309506415 | 0.118088668 |
| ENSG00000131023 | NONHSAT211283.1 | 0.39216543 | 0.04385714 | 0.181003258 | 0.069059744 |
| ENSG00000006652 | NONHSAT212541.1 | 7.49127756 | 3.34998039 | 33.73187429 | 16.46687161 |
| ENSG00000133612 | NONHSAT212882.1 | 1.06514931 | 0.05294183 | 0.546186379 | 0.166646588 |
| ENSG00000222355 | NONHSAT213501.1 | 0.12306751 | 0.03302083 | 0.431634286 | 0.121328549 |
| ENSG00000205482 | NONHSAT213634.1 | 0.18009771 | 0.08056366 | 0.332495116 | 0.126859747 |
| ENSG00000257923 | NONHSAT213777.1 | 0.36729287 | 0.10953483 | 0.452062325 | 0.172479262 |
| ENSG00000231721 | NONHSAT213987.1 | 0.39725864 | 0.01421712 | 0.087998483 | 0.022387014 |
| ENSG00000137547 | NONHSAT215798.1 | 1.04646248 | 0.29246205 | 0.965862912 | 0.184188334 |
| ENSG00000132561 | NONHSAT216135.1 | 0.77854287 | 0.32137626 | 0.221129282 | 0.084369374 |
| ENSG00000283959 | NONHSAT216183.1 | 0.30388539 | 0.07764193 | 2.083584928 | 0.733658392 |
| ENSG00000185122 | NONHSAT216666.1 | 0.36929397 | 0.06607896 | 1.22701005 | 0.207998714 |
| ENSG00000164733 | NONHSAT216786.1 | 2.92671416 | 1.20147387 | 2.214269105 | 0.473017384 |
| ENSG00000253616 | NONHSAT216849.1 | 1.41047109 | 0.55506635 | 2.603839719 | 0.397267706 |
| ENSG00000277586 | NONHSAT216864.1 | 1.18590362 | 0.09221993 | 0.47593004 | 0.072641681 |
| ENSG00000186918 | NONHSAT216884.1 | 1.00029502 | 0.27523222 | 2.698716533 | 1.191969471 |
| ENSG00000205268 | NONHSAT217154.1 | 0.4291249 | 0.06398725 | 0.528164904 | 0.201414582 |
| ENSG00000254166 | NONHSAT217608.1 | 0.11662827 | 0.02086867 | 1.50707457 | 0.032860901 |
| ENSG00000276685 | NONHSAT218147.1 | 0.04569355 | 0.00681341 | 0.056239407 | 0.02144678 |
| ENSG00000095209 | NONHSAT219954.1 | 26.1540081 | 0.05891319 | 0.121628432 | 0.046405951 |
| ENSG00000165219 | NONHSAT220162.1 | 0.57464703 | 0.0856862 | 0.884001076 | 0.269717022 |
| ENSG00000167118 | NONHSAT220218.1 | 0.05610267 | 0.02509658 | 0.103576357 | 0.039518386 |
| ENSG00000137076 | NONHSAT220672.1 | 0.11844393 | 0.03532259 | 0.145780226 | 0.055620795 |
| ENSG00000070061 | NONHSAT221160.1 | 9.41456613 | 0.07138225 | 4.123984024 | 0.224692029 |
| ENSG00000148296 | NONHSAT221417.1 | 1.8562768 | 0.92228695 | 0.761565577 | 0.290566724 |
| ENSG00000231771 | NONHSAT222559.1 | 0.05156324 | 0.01729398 | 0.035692331 | 0.00908021 |
| ENSG00000102226 | NONHSAT222687.1 | 0.71594398 | 0.0915044 | 0.755297547 | 0.144087614 |
| ENSG00000184205 | NONHSAT222719.1 | 1.13768974 | 0.17543106 | 2.389927865 | 0.27623745 |
| ENSG00000142748 | NONHSAT224838.1 | 0.66750452 | 0.32792517 | 0.086778241 | 0.039721906 |
| ENSG00000134183 | NONHSAT225350.1 | 0.08496918 | 0.02533968 | 0.47052864 | 0.07976247 |
| ENSG00000228453 | NONHSAT225433.1 | 0.12033157 | 0.02690562 | 0.055529404 | 0.014126807 |
| ENSG00000143570 | NONHSAT225595.1 | 0.85403782 | 0.18332804 | 0.819829809 | 0.384881487 |
| ENSG00000143669 | NONHSAT226146.1 | 0.4657382 | 0.22720809 | 0.312670024 | 0.11923606 |
| ENSG00000187634 | NONHSAT226249.1 | 0.758885 | 0.16973718 | 0.700524061 | 0.267277024 |
| ENSG00000169962 | NONHSAT226266.1 | 0.37187258 | 0.0554503 | 0.572065534 | 0.087314938 |
| ENSG00000169598 | NONHSAT226313.1 | 0.4322345 | 0.09663044 | 0.498691911 | 0.228271429 |
| ENSG00000080947 | NONHSAT226404.1 | 0.01432581 | 0.0032042 | 0.026448203 | 0.005045506 |
| ENSG00000215908 | NONHSAT226410.1 | 0.04015102 | 0.01796088 | 0.185297479 | 0.056535999 |
| ENSG00000122483 | NONHSAT226900.1 | 0.87987919 | 0.06668802 | 0.234012448 | 0.010505038 |
| ENSG00000233983 | NONHSAT226943.1 | 0.05037443 | 0.0112635 | 0.092993026 | 0.02955771 |
| ENSG00000135829 | NONHSAT227394.1 | 0.37741639 | 0.10126088 | 0.243844784 | 0.03986517 |
| ENSG00000092969 | NONHSAT227615.1 | 0.16830802 | 0.05019316 | 0.621352719 | 0.157994508 |
| ENSG00000078403 | NONHSAT228071.1 | 0.37476124 | 0.15234486 | 0.251593289 | 0.047996285 |
| ENSG00000230445 | NONHSAT228093.1 | 0.36221002 | 0.12495295 | 0.105071317 | 0.029152227 |
| ENSG00000213341 | NONHSAT228481.1 | 0.40568402 | 0.05729001 | 0.275916608 | 0.030080146 |
| ENSG00000271335 | NONHSAT228954.1 | 0.12667327 | 0.05754497 | 0.244977032 | 0.039643299 |
| ENSG00000279088 | NONHSAT229120.1 | 0.08467967 | 0.02525334 | 0.156308492 | 0.039765236 |
| ENSG00000278616 | NONHSAT229161.1 | 0.03212197 | 0.00638328 | 0.026357048 | 0.005028117 |
| ENSG00000225484 | NONHSAT229174.1 | 0.51066932 | 0.01692205 | 0.069839157 | 0.02664634 |
| ENSG00000138185 | NONHSAT229295.1 | 0.04048073 | 0.01810263 | 0.230405145 | 0.066509806 |
| ENSG00000165669 | NONHSAT229424.1 | 0.03460283 | 0.01547899 | 0.606812002 | 0.243642777 |
| ENSG00000107938 | NONHSAT229481.1 | 0.37585609 | 0.14705632 | 0.43364514 | 0.165403048 |
| ENSG00000255236 | NONHSAT229903.1 | 0.50912797 | 0.04792453 | 0.098941943 | 0.037750178 |
| ENSG00000054967 | NONHSAT229954.1 | 0.85998214 | 0.15387938 | 0.635077177 | 0.242306507 |
| ENSG00000109861 | NONHSAT229999.1 | 1.34989906 | 0.08622697 | 0.623020235 | 0.067921029 |
| ENSG00000245498 | NONHSAT230149.1 | 0.1141377 | 0.0340222 | 0.245822374 | 0.080371112 |
| ENSG00000121236 | NONHSAT230260.1 | 0.1190111 | 0.02661036 | 0.109840086 | 0.013971784 |
| ENSG00000052841 | NONHSAT230415.1 | 0.27108411 | 0.06612352 | 0.227464828 | 0.034718186 |
| ENSG00000172613 | NONHSAT230567.1 | 2.76139704 | 1.00620052 | 1.6991312 | 0.720149895 |
| ENSG00000095139 | NONHSAT230844.1 | 0.28102879 | 0.06282686 | 0.129708342 | 0.049488749 |
| ENSG00000171840 | NONHSAT230975.1 | 0.21635335 | 0.09254607 | 0.034734539 | 0.01325257 |
| ENSG00000256849 | NONHSAT231109.1 | 0.01222353 | 0.00546799 | 0.078978712 | 0.008610178 |
| ENSG00000139323 | NONHSAT231435.1 | 0.0371502 | 0.01107901 | 0.045724291 | 0.017445586 |
| ENSG00000111144 | NONHSAT231499.1 | 0.0398293 | 0.01781696 | 0.073532574 | 0.028055521 |
| ENSG00000151131 | NONHSAT231540.1 | 0.23322726 | 0.06811114 | 0.05271651 | 0.0067056 |
| ENSG00000186815 | NONHSAT231617.1 | 0.88088297 | 0.28135589 | 0.464651403 | 0.177193841 |
| ENSG00000111652 | NONHSAT231825.1 | 0.93346418 | 0.20868546 | 1.076987226 | 0.164381644 |
| ENSG00000273079 | NONHSAT231884.1 | 0.01389045 | 0.00621366 | 0.038460145 | 0.009784348 |
| ENSG00000063046 | NONHSAT232105.1 | 0.21471692 | 0.09604998 | 0.396408864 | 0.151245314 |
| ENSG00000136158 | NONHSAT232873.1 | 0.08036696 | 0.0359508 | 0.296745843 | 0.113163407 |
| ENSG00000102606 | NONHSAT233483.1 | 0.0427955 | 0.01913474 | 0.07900874 | 0.030129818 |
| ENSG00000237356 | NONHSAT233700.1 | 0.19105097 | 0.030154 | 0.22819694 | 0.094962566 |
| ENSG00000259158 | NONHSAT233784.1 | 0.25444615 | 0.11378489 | 0.289055252 | 0.124045903 |
| ENSG00000119608 | NONHSAT233807.1 | 0.11195263 | 0.05008011 | 0.206686153 | 0.078858762 |
| ENSG00000185100 | NONHSAT234030.1 | 0.6080363 | 0.27186547 | 0.84177142 | 0.214148564 |
| ENSG00000264657 | NONHSAT234112.1 | 1.23649174 | 0.11062473 | 0.913120916 | 0.174195474 |
| ENSG00000274012 | NONHSAT234214.1 | 37.6247463 | 2.38510143 | 50.83175266 | 23.93736659 |
| ENSG00000100567 | NONHSAT234288.1 | 0.04529296 | 0.00368383 | 0.038005016 | 0.005800744 |
| ENSG00000100578 | NONHSAT234291.1 | 0.27251627 | 0.04876222 | 0.704313446 | 0.23027381 |
| ENSG00000027075 | NONHSAT234306.1 | 0.35312096 | 0.07430005 | 0.766897716 | 0.234016951 |
| ENSG00000154001 | NONHSAT234323.1 | 1.68314688 | 0.75268212 | 0.485499004 | 0.222232502 |
| ENSG00000066427 | NONHSAT234495.1 | 0.20247237 | 0.09052954 | 0.186901532 | 0.071310163 |
| ENSG00000182218 | NONHSAT234538.1 | 0.51451718 | 0.09760701 | 0.2014849 | 0.04390939 |
| ENSG00000258982 | NONHSAT234545.1 | 0.13302458 | 0.02321425 | 0.083849499 | 0.00913728 |
| ENSG00000089902 | NONHSAT234583.1 | 1.1512513 | 0.12111718 | 0.625063431 | 0.09540406 |
| ENSG00000114062 | NONHSAT234679.1 | 0.05629328 | 0.02518185 | 0.155865942 | 0.03965265 |
| ENSG00000137876 | NONHSAT234862.1 | 0.16756869 | 0.04087378 | 0.112496049 | 0.042900133 |
| ENSG00000117899 | NONHSAT235022.1 | 0.92989225 | 0.30238098 | 0.312138272 | 0.119092824 |
| ENSG00000272887 | NONHSAT235041.1 | 0.21151901 | 0.09461945 | 0.390504902 | 0.148992724 |
| ENSG00000176700 | NONHSAT235042.1 | 0.36751917 | 0.08220174 | 1.017594286 | 0.12943915 |
| ENSG00000103599 | NONHSAT235413.1 | 0.03798049 | 0.00849496 | 0.666062113 | 0.013376615 |
| ENSG00000103148 | NONHSAT235747.1 | 0.91565467 | 0.35097671 | 0.482992905 | 0.184280475 |
| ENSG00000181019 | NONHSAT236105.1 | 0.02306231 | 0.00687769 | 0.198665995 | 0.043309047 |
| ENSG00000263072 | NONHSAT236334.1 | 0.18409988 | 0.07840252 | 0.032369895 | 0.012350367 |
| ENSG00000197912 | NONHSAT236917.1 | 2.18832271 | 0.21745015 | 1.282450198 | 0.24457921 |
| ENSG00000166582 | NONHSAT237050.1 | 1.00811362 | 0.30053088 | 1.447460308 | 0.670440398 |
| ENSG00000263874 | NONHSAT237173.1 | 0.10396229 | 0.03486827 | 0.167930422 | 0.054904501 |
| ENSG00000207127 | NONHSAT237285.1 | 0.3062179 | 0.06224339 | 0.179875067 | 0.058809777 |
| ENSG00000201524 | NONHSAT237360.1 | 0.15631122 | 0.06992318 | 0.721378237 | 0.110104686 |
| ENSG00000070495 | NONHSAT237513.1 | 0.20938066 | 0.0748947 | 0.386517741 | 0.117930189 |
| ENSG00000173894 | NONHSAT237557.1 | 2.25258162 | 0.67151407 | 0.554493366 | 0.211560666 |
| ENSG00000278876 | NONHSAT237594.1 | 0.01557968 | 0.00696931 | 0.618340655 | 0.054849216 |
| ENSG00000264569 | NONHSAT237614.1 | 0.31262244 | 0.0932309 | 0.577063427 | 0.146806248 |
| ENSG00000108839 | NONHSAT237697.1 | 0.0940148 | 0.03603652 | 0.904933487 | 0.018911529 |
| ENSG00000072818 | NONHSAT237706.1 | 0.01522535 | 0.00680757 | 0.231873697 | 0.016081608 |
| ENSG00000173757 | NONHSAT237892.1 | 1.27895219 | 0.51993253 | 0.429327027 | 0.081902433 |
| ENSG00000108785 | NONHSAT237898.1 | 0.19513799 | 0.06345468 | 1.571887324 | 0.149899868 |
| ENSG00000252628 | NONHSAT237955.1 | 0.42974324 | 0.12815891 | 0.793254336 | 0.201805707 |
| ENSG00000159217 | NONHSAT238028.1 | 0.36667196 | 0.06148969 | 0.338430527 | 0.129108481 |
| ENSG00000153944 | NONHSAT238066.1 | 0.11695757 | 0.0522991 | 0.107952131 | 0.032937208 |
| ENSG00000232457 | NONHSAT238135.1 | 0.86648504 | 0.42269597 | 0.727094387 | 0.221898807 |
| ENSG00000237854 | NONHSAT238158.1 | 0.7260609 | 0.16235076 | 0.167534543 | 0.042621169 |
| ENSG00000173821 | NONHSAT238233.1 | 0.11297038 | 0.04040909 | 0.166852095 | 0.063628697 |
| ENSG00000141562 | NONHSAT238294.1 | 0.43880599 | 0.02181027 | 0.900042292 | 0.205992854 |
| ENSG00000198081 | NONHSAT238335.1 | 0.3037859 | 0.06791444 | 0.560776022 | 0.053496242 |
| ENSG00000198796 | NONHSAT238539.1 | 0.10178033 | 0.02275403 | 0.093953061 | 0.035828803 |
| ENSG00000141452 | NONHSAT238783.1 | 0.23254722 | 0.02600651 | 0.214663513 | 0.081861482 |
| ENSG00000075643 | NONHSAT238849.1 | 1.1832213 | 0.0092861 | 0.057477397 | 0.014622381 |
| ENSG00000134775 | NONHSAT238853.1 | 0.07499165 | 0.01676781 | 0.034606394 | 0.008803945 |
| ENSG00000283615 | NONHSAT238859.1 | 1.74536045 | 0.47507048 | 0.980662386 | 0.320625518 |
| ENSG00000066044 | NONHSAT239106.1 | 0.28748177 | 0.01224763 | 0.505421711 | 0.173513948 |
| ENSG00000126456 | NONHSAT239473.1 | 0.36819976 | 0.16462964 | 0.339883896 | 0.129678853 |
| ENSG00000213753 | NONHSAT239603.1 | 0.08010793 | 0.03583492 | 0.73939816 | 0.338452401 |
| ENSG00000127616 | NONHSAT239761.1 | 0.38413422 | 0.1717706 | 0.354556846 | 0.135236804 |
| ENSG00000064607 | NONHSAT239826.1 | 0.61493156 | 0.09169307 | 0.945972286 | 0.144384702 |
| ENSG00000167232 | NONHSAT239861.1 | 0.03135427 | 0.00935053 | 0.501620005 | 0.07358975 |
| ENSG00000073008 | NONHSAT240043.1 | 0.42728053 | 0.08490927 | 0.438201106 | 0.133699269 |
| ENSG00000176920 | NONHSAT240109.1 | 0.21242294 | 0.08635243 | 0.712970565 | 0.108794209 |
| ENSG00000105053 | NONHSAT240136.1 | 1.2452685 | 0.18559501 | 1.723961928 | 0.584626921 |
| ENSG00000179820 | NONHSAT240178.1 | 0.14512983 | 0.01623034 | 0.301378746 | 0.051088735 |
| ENSG00000131037 | NONHSAT240192.1 | 0.22730021 | 0.08559382 | 0.309163495 | 0.117941278 |
| ENSG00000256060 | NONHSAT240221.1 | 1.36183473 | 0.60896207 | 1.38262338 | 0.287684541 |
| ENSG00000132305 | NONHSAT240797.1 | 0.87549252 | 0.27784855 | 0.208565118 | 0.03978783 |
| ENSG00000169432 | NONHSAT241216.1 | 1.23478139 | 0.12992805 | 0.312875725 | 0.102294065 |
| ENSG00000196141 | NONHSAT241349.1 | 0.16610516 | 0.03715216 | 0.229957606 | 0.058501738 |
| ENSG00000118263 | NONHSAT241370.1 | 0.01129447 | 0.00505239 | 0.166792959 | 0.023859318 |
| ENSG00000214595 | NONHSAT242002.1 | 1.22962131 | 0.54987009 | 0.52380448 | 0.066628526 |
| ENSG00000075292 | NONHSAT242181.1 | 0.06316006 | 0.00565072 | 0.034975763 | 0.008897914 |
| ENSG00000144228 | NONHSAT242608.1 | 0.1230986 | 0.04183534 | 0.131799401 | 0.064144063 |
| ENSG00000115221 | NONHSAT242679.1 | 1.08058626 | 0.16112732 | 1.662306371 | 0.760904763 |
| ENSG00000170144 | NONHSAT242783.1 | 0.28770729 | 0.01980015 | 0.081717411 | 0.031178353 |
| ENSG00000116044 | NONHSAT242785.1 | 0.52427803 | 0.13221735 | 0.067522151 | 0.019317751 |
| ENSG00000138448 | NONHSAT242842.1 | 1.48188579 | 0.11043479 | 0.797930264 | 0.086989541 |
| ENSG00000144445 | NONHSAT242935.1 | 0.64276251 | 0.06389532 | 1.582083938 | 0.201125193 |
| ENSG00000144468 | NONHSAT243004.1 | 2.48856891 | 1.22998124 | 0.34253996 | 0.138346321 |
| ENSG00000283491 | NONHSAT243033.1 | 3.25794268 | 1.06315151 | 2.275679404 | 1.0540582 |
| ENSG00000149639 | NONHSAT243327.1 | 0.22512214 | 0.04028183 | 0.249329035 | 0.063429874 |
| ENSG00000101391 | NONHSAT243787.1 | 1.72484746 | 0.56088333 | 1.447307755 | 0.662491213 |
| ENSG00000254806 | NONHSAT243867.1 | 0.31380661 | 0.11224748 | 0.46347804 | 0.088417399 |
| ENSG00000201742 | NONHSAT243892.1 | 0.40086264 | 0.11954608 | 0.49337985 | 0.188243496 |
| ENSG00000225806 | NONHSAT243972.1 | 0.18441977 | 0.03711283 | 0.195756005 | 0.097398068 |
| ENSG00000157551 | NONHSAT244263.1 | 0.0807915 | 0.01807035 | 0.074578344 | 0.028454523 |
| ENSG00000232969 | NONHSAT244347.1 | 0.73398312 | 0.36467786 | 0.301127652 | 0.114834401 |
| ENSG00000160218 | NONHSAT244623.1 | 0.52973678 | 0.1579041 | 0.814913958 | 0.124381137 |
| ENSG00000160298 | NONHSAT244668.1 | 0.32103531 | 0.0956943 | 0.197564375 | 0.07537845 |
| ENSG00000185837 | NONHSAT244695.1 | 0.40867285 | 0.12053687 | 0.040129149 | 0.006124952 |
| ENSG00000215012 | NONHSAT244718.1 | 0.17725358 | 0.04528783 | 0.116861069 | 0.053492031 |
| ENSG00000100100 | NONHSAT244833.1 | 0.13623758 | 0.01522912 | 0.086450969 | 0.01198999 |
| ENSG00000237037 | NONHSAT244910.1 | 0.08125887 | 0.02220669 | 0.041669003 | 0.003179991 |
| ENSG00000100285 | NONHSAT245136.1 | 0.15998649 | 0.04923407 | 0.16831262 | 0.053299112 |
| ENSG00000100344 | NONHSAT245267.1 | 0.15131497 | 0.03382801 | 0.244419278 | 0.02664634 |
| ENSG00000196639 | NONHSAT245364.1 | 0.03668411 | 0.01641001 | 0.067725942 | 0.025840067 |
| ENSG00000268279 | NONHSAT245382.1 | 1.04231573 | 0.34958287 | 0.661442837 | 0.068810522 |
| ENSG00000242531 | NONHSAT245768.1 | 3.76968273 | 0.69351098 | 2.342873355 | 0.388560576 |
| ENSG00000163848 | NONHSAT245805.1 | 0.79531148 | 0.05082416 | 0.314582005 | 0.080030378 |
| ENSG00000070476 | NONHSAT245822.1 | 0.68917806 | 0.1540728 | 0.238526217 | 0.060681612 |
| ENSG00000179348 | NONHSAT245839.1 | 0.17172533 | 0.06440582 | 0.3885867 | 0.03510694 |
| ENSG00000163584 | NONHSAT246032.1 | 1.23072431 | 0.5681187 | 0.732901851 | 0.111807615 |
| ENSG00000075651 | NONHSAT246043.1 | 0.27945582 | 0.01666306 | 0.458567275 | 0.139930676 |
| ENSG00000145012 | NONHSAT246124.1 | 0.83307767 | 0.24832404 | 0.640777316 | 0.293309657 |
| ENSG00000282206 | NONHSAT246150.1 | 1.85222997 | 0.48513186 | 1.660772188 | 0.801195783 |
| ENSG00000163702 | NONHSAT246264.1 | 1.79182499 | 0.04161985 | 0.730302893 | 0.294957464 |
| ENSG00000154781 | NONHSAT246326.1 | 0.08353143 | 0.03248154 | 0.020112469 | 0.005116658 |
| ENSG00000251474 | NONHSAT246807.1 | 1.42695152 | 0.07733798 | 1.47673763 | 0.608978435 |
| ENSG00000152601 | NONHSAT246930.1 | 0.11873009 | 0.00482857 | 0.129517087 | 0.038001395 |
| ENSG00000075420 | NONHSAT247017.1 | 0.29336462 | 0.11478095 | 0.304602642 | 0.129101014 |
| ENSG00000260261 | NONHSAT247147.1 | 0.19531225 | 0.08736963 | 0.360584094 | 0.137576779 |
| ENSG00000174227 | NONHSAT247198.1 | 0.13712267 | 0.0613395 | 0.253154902 | 0.096588387 |
| ENSG00000087269 | NONHSAT247243.1 | 0.06303991 | 0.01409992 | 0.407284186 | 0.066585198 |
| ENSG00000263327 | NONHSAT247388.1 | 0.47245084 | 0.18543266 | 0.112940983 | 0.047865543 |
| ENSG00000109814 | NONHSAT247470.1 | 1.04376276 | 0.31681023 | 0.354415938 | 0.140902684 |
| ENSG00000138660 | NONHSAT247710.1 | 0.64554504 | 0.17320928 | 0.238360135 | 0.090943611 |
| ENSG00000164040 | NONHSAT247786.1 | 0.11245964 | 0.00815551 | 0.016831825 | 0.002141027 |
| ENSG00000173542 | NONHSAT248339.1 | 0.39024116 | 0.04364194 | 0.720368806 | 0.343466976 |
| ENSG00000251022 | NONHSAT248396.1 | 0.34246921 | 0.12673849 | 0.26161331 | 0.016637344 |
| ENSG00000163624 | NONHSAT248418.1 | 0.42363893 | 0.10102274 | 0.886322147 | 0.357971104 |
| ENSG00000249001 | NONHSAT248434.1 | 0.36981114 | 0.15564215 | 1.686571715 | 0.091907971 |
| ENSG00000202000 | NONHSAT248438.1 | 0.13108378 | 0.02345524 | 0.145178932 | 0.03693385 |
| ENSG00000109381 | NONHSAT248635.1 | 0.23974422 | 0.07149701 | 0.442538994 | 0.112582927 |
| ENSG00000164134 | NONHSAT248639.1 | 0.17374015 | 0.08475634 | 0.097192365 | 0.037071587 |
| ENSG00000250326 | NONHSAT248665.1 | 0.25577027 | 0.03489564 | 0.016007091 | 0.006107324 |
| ENSG00000172239 | NONHSAT249085.1 | 0.10117731 | 0.01939098 | 0.186765676 | 0.030533544 |
| ENSG00000183474 | NONHSAT249203.1 | 14.5591404 | 0.10176462 | 6.089162465 | 0.160243891 |
| ENSG00000152359 | NONHSAT249239.1 | 0.37060181 | 0.14500054 | 0.17105061 | 0.065229792 |
| ENSG00000164176 | NONHSAT249291.1 | 0.40608001 | 0.01545281 | 0.191401081 | 0.012172185 |
| ENSG00000250362 | NONHSAT249356.1 | 0.22457967 | 0.0334873 | 0.207273535 | 0.052730859 |
| ENSG00000206997 | NONHSAT249373.1 | 0.20907673 | 0.09695645 | 0.057178362 | 0.021813078 |
| ENSG00000146067 | NONHSAT249741.1 | 1.1541485 | 0.41288105 | 0.781203613 | 0.270892298 |
| ENSG00000134057 | NONHSAT250114.1 | 0.08720521 | 0.01560391 | 0.064399054 | 0.02457073 |
| ENSG00000238160 | NONHSAT250412.1 | 0.47704657 | 0.16592498 | 0.538182761 | 0.261269526 |
| ENSG00000050393 | NONHSAT250836.1 | 0.22250209 | 0.07958819 | 0.328625432 | 0.125320621 |
| ENSG00000112033 | NONHSAT251080.1 | 0.48494603 | 0.21693239 | 0.895303922 | 0.341593075 |
| ENSG00000155115 | NONHSAT251401.1 | 0.43781543 | 0.18946256 | 0.143388985 | 0.039783534 |
| ENSG00000056972 | NONHSAT251421.1 | 0.13826874 | 0.0558493 | 0.197610143 | 0.056535279 |
| ENSG00000197442 | NONHSAT251543.1 | 1.04523998 | 0.2077103 | 0.428825477 | 0.163613506 |
| ENSG00000273132 | NONHSAT251647.1 | 0.37064019 | 0.14730739 | 0.228052406 | 0.058017051 |
| ENSG00000204623 | NONHSAT252146.1 | 0.39784237 | 0.07783171 | 1.560660608 | 0.140070676 |
| ENSG00000118420 | NONHSAT252469.1 | 6.6747952 | 0.1270604 | 3.408149633 | 0.200075944 |
| ENSG00000175048 | NONHSAT252870.1 | 1.46673995 | 0.49973157 | 1.28935801 | 0.622957771 |
| ENSG00000155034 | NONHSAT253080.1 | 0.15464963 | 0.04609801 | 0.095171016 | 0.018155712 |
| ENSG00000221740 | NONHSAT253167.1 | 0.25168085 | 0.05629258 | 0.232325701 | 0.088641241 |
| ENSG00000252480 | NONHSAT253306.1 | 0.04439443 | 0.0132394 | 0.081946786 | 0.020847449 |
| ENSG00000234456 | NONHSAT253510.1 | 0.02289589 | 0.00767913 | 0.0528324 | 0.012091762 |
| ENSG00000091732 | NONHSAT253707.1 | 0.12737211 | 0.05695068 | 0.176335205 | 0.044860077 |
| ENSG00000272831 | NONHSAT254238.1 | 1.4001578 | 0.69568437 | 1.005144227 | 0.219120732 |
| ENSG00000273623 | NONHSAT254291.1 | 0.82569714 | 0.03693616 | 1.219361816 | 0.290691735 |
| ENSG00000127995 | NONHSAT254318.1 | 0.88872665 | 0.42580681 | 0.761719267 | 0.268211641 |
| ENSG00000105875 | NONHSAT254582.1 | 1.13399926 | 0.33802284 | 0.348930236 | 0.133130381 |
| ENSG00000170356 | NONHSAT254615.1 | 0.2082902 | 0.07983906 | 0.054934841 | 0.020959767 |
| ENSG00000038945 | NONHSAT254867.1 | 0.52719511 | 0.2020775 | 0.556173649 | 0.106100879 |
| ENSG00000207027 | NONHSAT254930.1 | 0.00578728 | 0.00258884 | 0.021368879 | 0.004076527 |
| ENSG00000228801 | NONHSAT255066.1 | 0.35380407 | 0.01861426 | 0.153681845 | 0.029310498 |
| ENSG00000164944 | NONHSAT255241.1 | 0.78612806 | 0.15979235 | 0.791610655 | 0.050342617 |
| ENSG00000254024 | NONHSAT256143.1 | 0.89174032 | 0.20806731 | 0.501040454 | 0.054622918 |
| ENSG00000237984 | NONHSAT256588.1 | 0.02920503 | 0.01451046 | 0.041933272 | 0.013709996 |
| ENSG00000235387 | NONHSAT256613.1 | 0.05718835 | 0.02462664 | 0.007821081 | 0.002984045 |
| ENSG00000147905 | NONHSAT256623.1 | 0.08924052 | 0.03990126 | 0.082377605 | 0.031430243 |
| ENSG00000204860 | NONHSAT256647.1 | 0.23117975 | 0.03446027 | 0.029948016 | 0.014279011 |
| ENSG00000083223 | NONHSAT256827.1 | 0.12170296 | 0.04664958 | 0.192556317 | 0.048962271 |
| ENSG00000165030 | NONHSAT256874.1 | 0.10069979 | 0.02770765 | 0.243092875 | 0.109083014 |
| ENSG00000243738 | NONHSAT257049.1 | 0.34787147 | 0.17283916 | 0.570805453 | 0.054453017 |
| ENSG00000171448 | NONHSAT257066.1 | 0.77591333 | 0.01051825 | 0.086819917 | 0.016562578 |
| ENSG00000204054 | NONHSAT257139.1 | 0.0083804 | 0.00249922 | 0.056722214 | 0.003935404 |
| ENSG00000221676 | NONHSAT257191.1 | 0.64008999 | 0.12014523 | 0.17506048 | 0.033387872 |
| ENSG00000204778 | NONHSAT257488.1 | 2.19699305 | 0.61400872 | 1.267393808 | 0.580137333 |
| ENSG00000165091 | NONHSAT257507.1 | 0.33193301 | 0.14841404 | 0.45953131 | 0.11690581 |
| ENSG00000252847 | NONHSAT257638.1 | 0.34649978 | 0.14085625 | 0.290745601 | 0.088709211 |
| ENSG00000254483 | NONHSAT257670.1 | 0.11640976 | 0.02313297 | 0.549166143 | 0.109294673 |
| ENSG00000226609 | NONHSAT257766.1 | 0.73397816 | 0.05251034 | 0.812988586 | 0.37213771 |
| ENSG00000272896 | NONHSAT257971.1 | 1.03748166 | 0.20850953 | 0.23670616 | 0.098503618 |
| ENSG00000279073 | NONHSAT257978.1 | 0.24915773 | 0.11140352 | 0.804871129 | 0.219293765 |
| ENSG00000044446 | NONHSAT258078.1 | 0.10571112 | 0.04942026 | 0.03104712 | 0.013535516 |
| ENSG00000124313 | NONHSAT258169.1 | 0.11045993 | 0.03951111 | 0.489391298 | 0.031122925 |
| ENSG00000165175 | NONHSAT258596.1 | 0.11715447 | 0.04490611 | 0.061797072 | 0.023577973 |

**Supplemental table S3. Downregulated lncRNAs in CoCl_2_ induction and overexpression of HIF-1α plasmid models.**

| Gene | lncRNA_id | 18h_FPKM | 0h_FPKM | HIF_FPKM | NC_FPKM |
| --- | --- | --- | --- | --- | --- |
| ENSG00000283824 | ENST00000334146 | 0.107824225 | 0.289308347 | 0.066321005 | 0.303701684 |
| ENSG00000196143 | ENST00000359695 | 0.050773205 | 0.181613908 | 0.093689428 | 0.286043202 |
| ENSG00000151208 | ENST00000372387 | 0.051724212 | 0.254429475 | 0.047722139 | 0.182098256 |
| ENSG00000127957 | ENST00000394921 | 0.528797529 | 1.351224559 | 0.348629604 | 1.489395126 |
| ENSG00000214049 | ENST00000397381 | 2.529607538 | 5.588285019 | 0.119729993 | 12.14910912 |
| ENSG00000272968 | ENST00000405396 | 0.055291399 | 1.780165996 | 0.204157179 | 4.905599533 |
| ENSG00000009413 | ENST00000406170 | 0.163294901 | 0.350487726 | 0.060264094 | 0.22995588 |
| ENSG00000172965 | ENST00000409569 | 0.676943141 | 1.383822194 | 2.142277084 | 12.93816004 |
| ENSG00000147854 | ENST00000411561 | 0.389553039 | 0.836116488 | 0.431440838 | 0.987506495 |
| ENSG00000122140 | ENST00000415062 | 0.133501256 | 0.676556379 | 0.164312716 | 0.752049112 |
| ENSG00000223546 | ENST00000416381 | 0.024705252 | 0.176760588 | 0.045587451 | 0.208757172 |
| ENSG00000223546 | ENST00000420471 | 0.059316191 | 0.371325971 | 0.109453401 | 0.501216503 |
| ENSG00000023318 | ENST00000420996 | 0.129276899 | 0.289039214 | 0.119274318 | 0.273094557 |
| ENSG00000275625 | ENST00000423992 | 0.4999264 | 1.021960643 | 0.131784412 | 0.301738096 |
| ENSG00000265479 | ENST00000425797 | 0.182634297 | 0.408336477 | 0.112392715 | 0.557253774 |
| ENSG00000024526 | ENST00000425820 | 1.08733992 | 3.565635018 | 0.191184692 | 0.437520239 |
| ENSG00000233444 | ENST00000426710 | 0.197249872 | 0.882028396 | 0.363975994 | 0.833371883 |
| ENSG00000064655 | ENST00000429337 | 0.133620881 | 0.477957031 | 0.246564383 | 0.564542244 |
| ENSG00000159840 | ENST00000429630 | 1.304040821 | 3.018536319 | 0.991255961 | 2.16055775 |
| ENSG00000122884 | ENST00000431293 | 0.341628644 | 1.222138501 | 0.315195706 | 0.721682868 |
| ENSG00000273623 | ENST00000432193 | 0.194929285 | 0.435825796 | 0.179846962 | 0.411783754 |
| ENSG00000116957 | ENST00000434479 | 0.201242784 | 0.539964162 | 0.371343929 | 1.983991942 |
| ENSG00000234741 | ENST00000434796 | 0.864469005 | 2.164742482 | 1.436135241 | 3.165323219 |
| ENSG00000275266 | ENST00000447298 | 0.094860625 | 0.254525092 | 0.875565951 | 1.870270978 |
| ENSG00000215837 | ENST00000453324 | 1.554388036 | 3.985172659 | 0.828945359 | 2.189072754 |
| ENSG00000235084 | ENST00000454346 | 0.33360381 | 0.994501816 | 0.410597996 | 1.565963529 |
| ENSG00000049167 | ENST00000457499 | 0.121830803 | 0.544782245 | 0.224808702 | 0.85782561 |
| ENSG00000064419 | ENST00000459650 | 0.151638096 | 0.406841652 | 0.167943413 | 0.341673315 |
| ENSG00000166821 | ENST00000460165 | 0.485735841 | 1.448020559 | 0.298768568 | 0.684070732 |
| ENSG00000243753 | ENST00000463348 | 0.032509462 | 0.106608557 | 0.019996065 | 0.076301035 |
| ENSG00000205664 | ENST00000471090 | 0.107824225 | 0.964300025 | 0.199064321 | 0.607378051 |
| ENSG00000151276 | ENST00000472514 | 0.084106544 | 0.225670301 | 0.155197886 | 0.473834679 |
| ENSG00000099326 | ENST00000479047 | 0.436792335 | 0.93750847 | 0.806320841 | 5.78230027 |
| ENSG00000110958 | ENST00000482040 | 1.673635278 | 5.089121963 | 1.235941441 | 2.592633933 |
| ENSG00000104413 | ENST00000484521 | 0.648916028 | 1.392799789 | 0.479209671 | 2.558804158 |
| ENSG00000100150 | ENST00000488827 | 0.502090583 | 1.646509939 | 0.308828116 | 1.649986564 |
| ENSG00000272752 | ENST00000488972 | 0.090048855 | 0.483228797 | 0.166162954 | 0.760904763 |
| ENSG00000113163 | ENST00000494006 | 0.429743237 | 0.896746869 | 0.528925948 | 1.61384024 |
| ENSG00000269609 | ENST00000494270 | 0.279645388 | 0.625235319 | 0.12900415 | 0.886116939 |
| ENSG00000051382 | ENST00000495704 | 0.039232019 | 0.10526531 | 0.072393015 | 0.165753524 |
| ENSG00000064651 | ENST00000501702 | 0.016697 | 0.05972456 | 0.154113725 | 0.658395695 |
| ENSG00000249142 | ENST00000503901 | 0.044861884 | 0.320976809 | 0.082781544 | 0.379078907 |
| ENSG00000176783 | ENST00000515779 | 0.254472531 | 0.986200443 | 0.1565221 | 1.314014126 |
| ENSG00000104763 | ENST00000517747 | 0.060840842 | 0.163244979 | 0.112266769 | 0.342761683 |
| ENSG00000206573 | ENST00000522221 | 0.152319615 | 0.499503015 | 0.468637956 | 4.504733381 |
| ENSG00000247081 | ENST00000522856 | 0.083681764 | 0.224530552 | 0.154414058 | 0.353551708 |
| ENSG00000249395 | ENST00000523313 | 0.26772155 | 0.55865559 | 0.65902084 | 8.67172964 |
| ENSG00000198382 | ENST00000531215 | 0.105534963 | 0.283165919 | 0.194738748 | 0.445880498 |
| ENSG00000255248 | ENST00000531381 | 0.029499684 | 0.567222669 | 0.081679304 | 2.970415332 |
| ENSG00000166016 | ENST00000531489 | 0.112205345 | 0.250870379 | 0.103523646 | 0.632057069 |
| ENSG00000196935 | ENST00000541870 | 0.289555152 | 0.604215851 | 0.178100875 | 0.407785854 |
| ENSG00000067715 | ENST00000548384 | 0.138846279 | 0.434596597 | 0.128103283 | 0.293309657 |
| ENSG00000140006 | ENST00000554777 | 0.812205356 | 1.888606077 | 0.299744936 | 0.686306257 |
| ENSG00000231431 | ENST00000560285 | 0.0571673 | 0.127815501 | 0.158285943 | 0.644051252 |
| ENSG00000128891 | ENST00000561460 | 0.087320101 | 0.331889976 | 0.080604877 | 0.307415765 |
| ENSG00000133740 | ENST00000566000 | 0.235950796 | 0.49239288 | 0.217768398 | 0.609186929 |
| ENSG00000260647 | ENST00000566532 | 0.132728886 | 0.474822836 | 0.122459213 | 0.280386802 |
| ENSG00000231305 | ENST00000567253 | 0.061290959 | 0.16445271 | 0.113097349 | 0.258951559 |
| ENSG00000102893 | ENST00000568688 | 0.169359345 | 0.378656143 | 0.078127726 | 0.954007774 |
| ENSG00000256087 | ENST00000569091 | 0.220919857 | 0.724464373 | 0.135884371 | 0.725994088 |
| ENSG00000260317 | ENST00000569134 | 0.027538486 | 0.246283829 | 0.152498202 | 0.465395489 |
| ENSG00000125148 | ENST00000569778 | 0.11195263 | 0.500610711 | 0.413372306 | 1.261267035 |
| ENSG00000199130 | ENST00000570945 | 0.015441742 | 0.165716781 | 0.028493927 | 0.326203347 |
| ENSG00000261970 | ENST00000571144 | 0.135441329 | 0.484468707 | 0.249923571 | 1.14446711 |
| ENSG00000233098 | ENST00000577537 | 0.013336992 | 0.059638088 | 0.541648387 | 1.746756399 |
| ENSG00000146872 | ENST00000583426 | 0.058238978 | 0.130211573 | 0.053732836 | 0.123028538 |
| ENSG00000189159 | ENST00000584339 | 0.124267419 | 0.777927909 | 0.45884326 | 1.225115024 |
| ENSG00000175322 | ENST00000585516 | 0.059887913 | 0.214216886 | 0.221129282 | 0.506047505 |
| ENSG00000226803 | ENST00000586234 | 0.116002258 | 0.36309354 | 0.10702678 | 0.245052176 |
| ENSG00000267073 | ENST00000586506 | 0.36549241 | 2.778358364 | 0.337213053 | 1.286738415 |
| ENSG00000272635 | ENST00000586784 | 0.075772817 | 0.474346286 | 0.139820046 | 0.533525684 |
| ENSG00000186185 | ENST00000591013 | 0.193412326 | 1.124345252 | 0.178538233 | 0.544749575 |
| ENSG00000131849 | ENST00000595059 | 0.009201586 | 0.057602955 | 0.033975806 | 0.077752579 |
| ENSG00000063169 | ENST00000599924 | 0.144496999 | 0.387706826 | 0.266633577 | 0.610493356 |
| ENSG00000100503 | ENST00000602615 | 0.601293965 | 1.434041548 | 0.184923287 | 1.83466817 |
| ENSG00000269950 | ENST00000602654 | 0.272740564 | 0.60979741 | 0.125818862 | 0.28807917 |
| ENSG00000154710 | ENST00000603786 | 0.207112366 | 0.740833398 | 0.382174793 | 1.750080955 |
| ENSG00000283792 | ENST00000604157 | 0.016568989 | 0.044457049 | 0.061179101 | 0.16334867 |
| ENSG00000163636 | ENST00000605919 | 0.475664763 | 1.914317262 | 0.219430503 | 0.669943289 |
| ENSG00000168291 | ENST00000607214 | 0.354416882 | 1.109419964 | 0.163497238 | 0.374348867 |
| ENSG00000272079 | ENST00000607794 | 0.039170187 | 0.140110335 | 0.072278921 | 0.386167068 |
| ENSG00000272644 | ENST00000607956 | 0.218013016 | 1.267354078 | 0.402494088 | 1.074662302 |
| ENSG00000163399 | ENST00000608511 | 0.336996392 | 0.715809962 | 0.466541109 | 0.949156887 |
| ENSG00000229358 | ENST00000609151 | 0.42124549 | 0.941826931 | 0.38885022 | 1.186446109 |
| ENSG00000151690 | ENST00000609166 | 0.346792798 | 1.033819329 | 0.640137801 | 2.604663086 |
| ENSG00000183735 | ENST00000610945 | 0.530814301 | 1.729353698 | 0.279995887 | 0.694123121 |
| ENSG00000213995 | ENST00000611744 | 0.258257473 | 0.597803036 | 0.056093308 | 0.385103389 |
| ENSG00000166260 | ENST00000616755 | 0.96518384 | 1.942298273 | 0.445252186 | 1.35939949 |
| ENSG00000130818 | ENST00000619671 | 0.115732172 | 0.258755711 | 0.213663916 | 0.733444871 |
| ENSG00000140943 | ENST00000622369 | 0.324882142 | 0.653780138 | 0.299897556 | 0.686306257 |
| ENSG00000139370 | ENST00000623017 | 0.285854767 | 0.596521255 | 0.117276233 | 0.715658228 |
| ENSG00000278616 | ENST00000623504 | 0.210821258 | 0.439941453 | 0.129716897 | 0.296903461 |
| ENSG00000100425 | ENST00000624882 | 0.056581637 | 0.151816902 | 0.052203728 | 0.278910649 |
| ENSG00000132530 | ENST00000624952 | 0.045380677 | 0.094696033 | 0.027912949 | 0.234331186 |
| ENSG00000068028 | ENST00000629828 | 0.188760637 | 0.393887549 | 0.116103735 | 0.443028923 |
| ENSG00000107938 | ENST00000630227 | 0.258890457 | 0.926151833 | 0.238859246 | 0.546900298 |
| ENSG00000215908 | ENST00000633763 | 0.121681684 | 0.272057718 | 0.112266769 | 0.42838782 |
| ENSG00000150907 | MSTRG.15015.47 | 0.276764854 | 0.5940573 | 0.255454654 | 0.779533086 |
| ENSG00000198538 | MSTRG.29725.2 | 0.527195113 | 2.222724368 | 0.973233125 | 3.181859272 |
| ENSG00000277739 | MSTRG.36148.2 | 1.098092071 | 46.22263492 | 1.486472853 | 24.12441478 |
| ENSG00000204525 | MSTRG.48883.1 | 0.155334274 | 0.347298681 | 1.86383183 | 7.000250876 |
| ENSG00000276700 | MSTRG.60138.6 | 1.272299302 | 9.118556896 | 5.522614108 | 14.31034712 |
| ENSG00000228794 | NONHSAT000161.2 | 0.036678695 | 0.085288291 | 0.027086379 | 0.061986338 |
| ENSG00000117000 | NONHSAT002625.2 | 0.296758016 | 0.862557402 | 0.273936275 | 1.358202108 |
| ENSG00000162664 | NONHSAT004388.2 | 0.167928945 | 0.450578203 | 0.309871454 | 0.709492279 |
| ENSG00000155380 | NONHSAT005297.2 | 0.03296218 | 0.147394666 | 0.486774412 | 1.856818862 |
| ENSG00000201558 | NONHSAT005889.2 | 0.102912977 | 0.674966807 | 0.379929958 | 0.869605444 |
| ENSG00000269501 | NONHSAT006207.2 | 0.09343415 | 0.250697647 | 0.172409681 | 0.394755103 |
| ENSG00000143337 | NONHSAT008230.2 | 0.061140182 | 0.164048153 | 0.075251047 | 0.258314532 |
| ENSG00000158711 | NONHSAT009019.2 | 0.503361699 | 1.294247812 | 0.348429436 | 0.797505245 |
| ENSG00000187134 | NONHSAT011122.2 | 1.477774717 | 7.329100764 | 0.743943931 | 1.70278147 |
| ENSG00000151632 | NONHSAT011124.2 | 2.821206278 | 6.608175639 | 0.743943931 | 1.70278147 |
| ENSG00000165983 | NONHSAT011597.2 | 0.509899478 | 1.477575669 | 0.753051389 | 2.642677286 |
| ENSG00000095787 | NONHSAT011933.2 | 0.047641374 | 0.18869009 | 0.012565024 | 0.03354871 |
| ENSG00000278217 | NONHSAT022144.2 | 2.595333925 | 8.254596313 | 2.621248897 | 8.954400302 |
| ENSG00000110723 | NONHSAT024033.2 | 0.147062035 | 3.156588496 | 0.814376492 | 4.556285669 |
| ENSG00000160588 | NONHSAT024449.2 | 1.020656765 | 2.921051963 | 9.872968434 | 22.16492251 |
| ENSG00000120451 | NONHSAT025148.2 | 0.008220104 | 0.051458767 | 0.030351795 | 0.092608329 |
| ENSG00000214772 | NONHSAT027079.2 | 0.106896705 | 0.239001243 | 0.19735194 | 0.602153294 |
| ENSG00000123349 | NONHSAT028445.2 | 1.164438483 | 3.77510415 | 0.403015014 | 1.844887683 |
| ENSG00000135502 | NONHSAT028947.2 | 0.476642157 | 1.979155684 | 0.062823254 | 0.191805682 |
| ENSG00000256268 | NONHSAT029197.2 | 0.068195535 | 0.243958724 | 0.097916747 | 0.650937629 |
| ENSG00000073060 | NONHSAT031684.2 | 0.08321479 | 0.223277592 | 0.051184124 | 0.195308509 |
| ENSG00000102780 | NONHSAT033428.2 | 0.170229342 | 6.61168333 | 0.179563555 | 5.822090062 |
| ENSG00000231607 | NONHSAT033788.2 | 0.09204994 | 0.411613251 | 0.169855464 | 0.388906879 |
| ENSG00000102606 | NONHSAT035213.2 | 0.148823257 | 0.382655603 | 0.034327077 | 0.419163595 |
| ENSG00000225210 | NONHSAT035473.2 | 0.207978945 | 1.302059833 | 0.191886926 | 0.439350867 |
| ENSG00000174373 | NONHSAT036395.2 | 0.049361438 | 0.13244404 | 0.04554218 | 0.139044834 |
| ENSG00000151338 | NONHSAT036457.2 | 0.123957526 | 0.351062243 | 0.114405343 | 0.23275252 |
| ENSG00000100644 | NONHSAT037281.2 | 2.414535146 | 21.63174679 | 1.998077674 | 4.69025602 |
| ENSG00000119640 | NONHSAT037837.2 | 0.090475005 | 0.210379854 | 0.016694931 | 0.08919658 |
| ENSG00000259216 | NONHSAT042350.2 | 0.034590792 | 0.433113848 | 0.12772255 | 0.292289095 |
| ENSG00000225151 | NONHSAT047922.2 | 0.019531225 | 0.122267648 | 0.072116819 | 0.19255246 |
| ENSG00000141551 | NONHSAT056661.2 | 0.021974787 | 0.746803377 | 0.649032549 | 9.872076114 |
| ENSG00000257267 | NONHSAT058922.2 | 0.248147109 | 0.873843148 | 0.085884323 | 0.32762826 |
| ENSG00000160953 | NONHSAT060283.2 | 0.023715156 | 0.254504923 | 0.043760473 | 0.534353591 |
| ENSG00000167772 | NONHSAT060897.2 | 0.055566732 | 0.262032127 | 0.662080668 | 1.472607747 |
| ENSG00000196235 | NONHSAT066299.2 | 0.569163753 | 1.69672638 | 0.23350802 | 1.33593966 |
| ENSG00000206674 | NONHSAT066369.2 | 0.064428993 | 0.2881026 | 0.059443908 | 0.136104805 |
| ENSG00000142541 | NONHSAT067206.2 | 0.535924181 | 1.078472895 | 0.494709714 | 1.320878732 |
| ENSG00000105497 | NONHSAT067453.2 | 0.062172568 | 0.166818196 | 0.05736207 | 0.131338158 |
| ENSG00000115128 | NONHSAT069557.2 | 0.12050174 | 0.484985993 | 0.333647581 | 0.678790986 |
| ENSG00000119777 | NONHSAT069714.2 | 0.143385484 | 0.470205242 | 0.088194183 | 0.874995589 |
| ENSG00000279254 | NONHSAT070518.2 | 0.073332138 | 0.185815952 | 0.022552729 | 0.051637501 |
| ENSG00000223973 | NONHSAT073640.2 | 3.948915262 | 9.137620024 | 9.603922808 | 23.56946342 |
| ENSG00000279227 | NONHSAT073924.2 | 0.312295085 | 0.930978663 | 0.384371317 | 0.952874777 |
| ENSG00000169967 | NONHSAT074153.2 | 0.404487079 | 1.848925445 | 0.248878169 | 1.012662863 |
| ENSG00000152117 | NONHSAT074478.2 | 0.049956752 | 0.178693483 | 0.276642467 | 1.758828413 |
| ENSG00000224043 | NONHSAT074578.2 | 0.013398105 | 0.071898193 | 0.024722898 | 0.056606392 |
| ENSG00000266705 | NONHSAT075862.2 | 0.012857467 | 0.080489178 | 0.047474729 | 0.144853224 |
| ENSG00000204217 | NONHSAT076456.2 | 0.155091943 | 0.416101653 | 0.286329647 | 1.31051304 |
| ENSG00000226539 | NONHSAT077151.2 | 0.282747257 | 1.16320338 | 0.730755247 | 1.911351214 |
| ENSG00000229539 | NONHSAT078253.2 | 0.113227717 | 1.046391856 | 0.069644609 | 1.222514151 |
| ENSG00000198900 | NONHSAT079756.2 | 0.385657508 | 0.996402517 | 0.316443628 | 1.387975118 |
| ENSG00000276859 | NONHSAT080588.2 | 0.005542704 | 0.01982605 | 0.010227693 | 0.046835351 |
| ENSG00000157540 | NONHSAT082032.2 | 0.460249701 | 1.029033129 | 0.424638659 | 1.620337263 |
| ENSG00000169662 | NONHSAT083648.2 | 0.006445406 | 0.017293977 | 0.011893406 | 0.03631176 |
| ENSG00000100345 | NONHSAT085104.2 | 0.246597289 | 0.913676873 | 0.195080974 | 1.28990847 |
| ENSG00000128165 | NONHSAT087491.2 | 0.020305134 | 0.054481678 | 0.074974389 | 0.371730152 |
| ENSG00000144674 | NONHSAT089010.2 | 0.163509762 | 0.438720882 | 0.150858471 | 0.460586012 |
| ENSG00000235257 | NONHSAT089040.2 | 0.016855533 | 0.045225889 | 0.03110273 | 0.071213874 |
| ENSG00000185219 | NONHSAT089322.2 | 0.09665915 | 2.550156121 | 0.133815875 | 1.361245913 |
| ENSG00000082781 | NONHSAT091571.2 | 0.041607395 | 0.086822311 | 0.076802247 | 0.214847176 |
| ENSG00000243926 | NONHSAT092857.2 | 0.124143276 | 0.33307366 | 0.04581516 | 0.384621518 |
| ENSG00000230457 | NONHSAT092863.2 | 0.020047174 | 0.340647154 | 0.074021901 | 0.211746032 |
| ENSG00000053524 | NONHSAT093645.2 | 0.05967223 | 0.160109421 | 0.110110385 | 0.252112503 |
| ENSG00000240005 | NONHSAT095890.2 | 0.273616336 | 0.652554686 | 0.168297157 | 0.642188713 |
| ENSG00000170006 | NONHSAT098812.2 | 0.178802042 | 0.439760298 | 0.165051532 | 0.818341198 |
| ENSG00000205129 | NONHSAT099610.2 | 0.125167058 | 0.909527006 | 0.057770634 | 0.396618913 |
| ENSG00000164236 | NONHSAT100461.2 | 0.067905694 | 0.30364912 | 0.125303211 | 0.478132307 |
| ENSG00000251493 | NONHSAT102122.2 | 0.156804315 | 4.487600048 | 1.157964063 | 11.04122568 |
| ENSG00000280047 | NONHSAT104293.2 | 0.076590089 | 0.273959962 | 0.141328121 | 0.431488898 |
| ENSG00000250999 | NONHSAT105610.2 | 0.253606978 | 0.60483385 | 0.155989712 | 0.476252202 |
| ENSG00000054598 | NONHSAT106538.2 | 0.192290011 | 3.640079593 | 6.507692381 | 19.63308732 |
| ENSG00000112699 | NONHSAT106632.2 | 0.081620637 | 0.291954048 | 0.150610756 | 0.459829713 |
| ENSG00000111859 | NONHSAT107742.2 | 0.099813188 | 0.357028143 | 0.184180623 | 0.421706254 |
| ENSG00000112079 | NONHSAT112221.2 | 0.107127086 | 1.341346121 | 2.373125907 | 6.638187799 |
| ENSG00000199094 | NONHSAT113497.2 | 0.032536061 | 0.127306526 | 0.435438389 | 1.300155592 |
| ENSG00000156508 | NONHSAT113572.2 | 0.078799885 | 0.338277397 | 0.116383833 | 0.244138325 |
| ENSG00000203875 | NONHSAT113853.2 | 0.204977187 | 0.595787072 | 0.094558712 | 0.288697211 |
| ENSG00000026297 | NONHSAT116065.2 | 0.021621126 | 0.07733798 | 0.039896455 | 0.091348288 |
| ENSG00000196204 | NONHSAT118886.2 | 0.022023468 | 0.413626834 | 0.691150633 | 1.426717888 |
| ENSG00000233834 | NONHSAT119416.2 | 0.019508229 | 0.087233578 | 0.035997626 | 0.082421395 |
| ENSG00000146592 | NONHSAT119766.2 | 0.09149925 | 0.368239584 | 0.084462634 | 0.740934715 |
| ENSG00000122641 | NONHSAT120258.2 | 0.12870784 | 0.276251848 | 0.237595318 | 1.450068795 |
| ENSG00000158604 | NONHSAT120387.2 | 0.069423139 | 0.558788139 | 0.064051641 | 0.146654829 |
| ENSG00000205485 | NONHSAT121614.2 | 0.067536641 | 1.207995412 | 0.249371337 | 0.760873048 |
| ENSG00000006377 | NONHSAT122137.2 | 0.074859891 | 0.234315635 | 0.069067734 | 0.158139845 |
| ENSG00000106554 | NONHSAT123438.2 | 0.330278856 | 0.763066487 | 0.203252828 | 0.503873429 |
| ENSG00000105887 | NONHSAT123532.2 | 0.152475361 | 19.77284682 | 0.281355676 | 27.27084215 |
| ENSG00000133624 | NONHSAT124030.2 | 0.017057985 | 0.259338873 | 0.094460966 | 0.264245806 |
| ENSG00000283128 | NONHSAT124386.2 | 0.107668522 | 0.706156609 | 0.198743144 | 0.404333981 |
| ENSG00000003989 | NONHSAT125277.2 | 0.108294047 | 0.258273278 | 0.199897788 | 0.457537505 |
| ENSG00000198363 | NONHSAT126875.2 | 1.796637389 | 5.738570757 | 3.000638348 | 6.747139364 |
| ENSG00000197275 | NONHSAT127768.2 | 0.162441071 | 0.87170685 | 0.299744936 | 1.14376748 |
| ENSG00000253105 | NONHSAT127859.2 | 0.037486401 | 0.083812653 | 0.017292977 | 0.105581177 |
| ENSG00000170881 | NONHSAT128586.2 | 0.020206084 | 0.162647742 | 0.037285346 | 0.142273516 |
| ENSG00000276443 | NONHSAT129053.2 | 0.102277711 | 2.972805862 | 0.094364147 | 0.360074947 |
| ENSG00000022567 | NONHSAT129412.2 | 0.012423636 | 0.144442254 | 0.183468086 | 1.032266355 |
| ENSG00000137145 | NONHSAT130328.2 | 0.055944815 | 0.137595231 | 0.02580809 | 0.256048231 |
| ENSG00000255872 | NONHSAT131127.2 | 0.350542791 | 0.877804603 | 0.064684027 | 0.246821473 |
| ENSG00000184925 | NONHSAT135627.2 | 0.103126489 | 0.3688797 | 0.190294503 | 0.435704802 |
| ENSG00000102054 | NONHSAT136388.2 | 1.772156354 | 8.688561463 | 1.497781428 | 4.39016726 |
| ENSG00000184634 | NONHSAT137431.2 | 0.065749957 | 0.176416863 | 0.121325331 | 0.462953504 |
| ENSG00000273974 | NONHSAT137629.2 | 0.227222488 | 0.533460436 | 0.174779641 | 0.520010717 |
| ENSG00000101811 | NONHSAT137899.2 | 0.150953915 | 0.347151677 | 0.039792603 | 0.577012946 |
| ENSG00000186376 | NONHSAT138693.2 | 0.212241536 | 0.721311491 | 0.470126822 | 1.195594724 |
| ENSG00000224470 | NONHSAT143517.2 | 1.229783288 | 8.674128357 | 0.611832806 | 9.138720636 |
| ENSG00000140836 | NONHSAT143628.2 | 0.025661832 | 0.091791438 | 0.047352582 | 0.1806881 |
| ENSG00000103035 | NONHSAT143668.2 | 0.024036251 | 0.064492818 | 0.044352974 | 1.557109416 |
| ENSG00000103187 | NONHSAT144079.2 | 0.098429639 | 0.616180522 | 0.181627625 | 0.831721642 |
| ENSG00000072849 | NONHSAT145225.2 | 0.07523759 | 3.857843654 | 0.092602071 | 5.297818189 |
| ENSG00000141499 | NONHSAT145443.2 | 0.042999107 | 1.499775414 | 0.079344248 | 1.93778571 |
| ENSG00000263934 | NONHSAT146441.2 | 108.3505179 | 218.1560514 | 11.50106533 | 46.15934658 |
| ENSG00000060656 | NONHSAT148604.1 | 0.054743357 | 0.783353092 | 0.101015364 | 0.693864696 |
| ENSG00000162510 | NONHSAT148633.1 | 0.011348623 | 0.101493678 | 0.167592651 | 0.543393474 |
| ENSG00000126070 | NONHSAT148716.1 | 0.03963873 | 0.815355479 | 1.536609397 | 5.638144888 |
| ENSG00000225099 | NONHSAT148827.1 | 0.043679234 | 0.19531736 | 0.080599253 | 0.184542807 |
| ENSG00000227016 | NONHSAT149187.1 | 0.056646117 | 0.177305506 | 0.10457966 | 0.279228496 |
| ENSG00000162695 | NONHSAT149465.1 | 0.14904638 | 0.333240114 | 0.137584186 | 0.419792027 |
| ENSG00000121957 | NONHSAT149516.1 | 0.236699846 | 0.529217038 | 0.218385596 | 1.00004626 |
| ENSG00000081026 | NONHSAT149623.1 | 0.083400953 | 0.298322174 | 0.15389589 | 0.352365293 |
| ENSG00000169242 | NONHSAT149868.1 | 4.885780342 | 13.90343837 | 7.304874462 | 32.18617323 |
| ENSG00000260238 | NONHSAT149880.1 | 0.505005478 | 4.322884334 | 0.665887603 | 10.87119466 |
| ENSG00000116667 | NONHSAT150159.1 | 1.683146881 | 6.915183144 | 1.359337907 | 12.59292812 |
| ENSG00000228106 | NONHSAT150616.1 | 0.092997133 | 0.332686907 | 0.085801637 | 0.392908727 |
| ENSG00000116285 | NONHSAT151071.1 | 0.474529525 | 1.082187445 | 4.379874829 | 11.39414356 |
| ENSG00000076864 | NONHSAT151240.1 | 0.037458152 | 0.26800475 | 0.069119782 | 0.58026547 |
| ENSG00000199756 | NONHSAT151331.1 | 0.036362083 | 0.130065849 | 0.067097257 | 0.204854642 |
| ENSG00000184007 | NONHSAT151380.1 | 0.906170806 | 3.752102255 | 0.371720803 | 2.694202938 |
| ENSG00000159592 | NONHSAT151531.1 | 0.042961943 | 0.345819744 | 0.07927567 | 0.302500301 |
| ENSG00000086015 | NONHSAT151536.1 | 0.058513205 | 0.209324487 | 0.053985845 | 0.123607837 |
| ENSG00000116171 | NONHSAT151605.1 | 0.256221483 | 0.687480145 | 0.236396779 | 0.541262151 |
| ENSG00000023909 | NONHSAT151947.1 | 1.925821619 | 5.879808311 | 0.306477327 | 0.935231411 |
| ENSG00000160710 | NONHSAT152350.1 | 0.449159347 | 2.744940341 | 0.41454707 | 1.686754709 |
| ENSG00000075391 | NONHSAT152582.1 | 0.160345057 | 0.717003728 | 0.295877259 | 0.677450692 |
| ENSG00000162783 | NONHSAT152615.1 | 0.009686635 | 0.043315045 | 0.008937148 | 0.068204784 |
| ENSG00000143473 | NONHSAT152847.1 | 0.053371834 | 0.111371211 | 0.032828186 | 0.150329073 |
| ENSG00000266028 | NONHSAT154489.1 | 0.215314836 | 0.770251811 | 0.170333697 | 0.433159466 |
| ENSG00000078403 | NONHSAT155198.1 | 0.122733254 | 0.439064573 | 0.226589264 | 0.691361189 |
| ENSG00000156515 | NONHSAT155612.1 | 0.212353132 | 0.569747085 | 0.030141949 | 0.391071872 |
| ENSG00000120008 | NONHSAT156229.1 | 0.303708561 | 0.633792872 | 0.093403208 | 0.285169343 |
| ENSG00000197321 | NONHSAT156822.1 | 0.019874837 | 0.053327128 | 0.110059678 | 0.223911461 |
| ENSG00000266412 | NONHSAT156969.1 | 0.058136804 | 0.675922175 | 0.053638567 | 0.491250794 |
| ENSG00000225484 | NONHSAT157230.1 | 0.141534646 | 0.405059572 | 0.052233457 | 0.119595509 |
| ENSG00000075826 | NONHSAT157387.1 | 0.04156101 | 0.111514338 | 0.076690594 | 0.175593407 |
| ENSG00000107821 | NONHSAT157405.1 | 0.043185897 | 0.69520818 | 0.079688923 | 0.851452643 |
| ENSG00000175029 | NONHSAT157658.1 | 0.164592608 | 0.392541174 | 0.101238356 | 0.695396406 |
| ENSG00000148655 | NONHSAT158312.1 | 0.036282458 | 0.08112086 | 0.033475164 | 0.153291762 |
| ENSG00000255073 | NONHSAT159141.1 | 0.190448152 | 0.511000567 | 0.351425097 | 1.072935996 |
| ENSG00000126391 | NONHSAT159231.1 | 0.180097709 | 0.483228797 | 0.664990232 | 1.521809526 |
| ENSG00000110172 | NONHSAT159521.1 | 0.23400691 | 0.470889784 | 0.07200365 | 0.933724368 |
| ENSG00000204381 | NONHSAT159647.1 | 0.162087938 | 0.338229526 | 0.398994139 | 1.521784154 |
| ENSG00000166801 | NONHSAT160346.1 | 0.177050642 | 1.22715768 | 0.571938701 | 1.683038663 |
| ENSG00000109861 | NONHSAT160605.1 | 0.217060995 | 0.58240676 | 0.200266267 | 0.764176526 |
| ENSG00000247498 | NONHSAT161871.1 | 0.044460615 | 0.23856991 | 0.04102055 | 0.093922055 |
| ENSG00000123106 | NONHSAT161993.1 | 2.316808526 | 7.120304744 | 1.166431928 | 2.906874566 |
| ENSG00000076067 | NONHSAT162263.1 | 0.196989304 | 0.469804893 | 0.969690638 | 2.589225872 |
| ENSG00000022840 | NONHSAT162877.1 | 1.497627436 | 3.252772881 | 0.263324683 | 1.305588827 |
| ENSG00000245614 | NONHSAT163429.1 | 0.039591372 | 0.424901199 | 0.036528057 | 0.167271776 |
| ENSG00000205420 | NONHSAT163554.1 | 0.417267305 | 0.88631244 | 0.57766895 | 2.644330062 |
| ENSG00000169372 | NONHSAT163858.1 | 0.074051349 | 0.149018043 | 0.068356538 | 0.234647726 |
| ENSG00000276908 | NONHSAT164013.1 | 0.214716923 | 1.440201873 | 0.396408864 | 1.436301125 |
| ENSG00000111011 | NONHSAT164133.1 | 0.083400953 | 0.447554188 | 0.307948497 | 1.174472647 |
| ENSG00000083535 | NONHSAT165882.1 | 1.216931414 | 2.930207841 | 0.34564464 | 1.054618764 |
| ENSG00000133112 | NONHSAT166712.1 | 0.063645285 | 0.284598151 | 0.23500295 | 1.075593929 |
| ENSG00000136161 | NONHSAT166747.1 | 0.148526796 | 0.61990498 | 0.18280608 | 0.418346045 |
| ENSG00000165516 | NONHSAT168413.1 | 0.621337097 | 1.296590549 | 0.382369383 | 1.166672007 |
| ENSG00000198604 | NONHSAT169213.1 | 0.068846216 | 0.184724582 | 0.127038712 | 0.290872181 |
| ENSG00000151332 | NONHSAT169225.1 | 0.443812212 | 1.786220733 | 0.409472993 | 1.562468075 |
| ENSG00000183379 | NONHSAT169495.1 | 0.003683635 | 0.016471851 | 0.00679724 | 0.025936924 |
| ENSG00000171956 | NONHSAT170867.1 | 0.010715018 | 0.023956796 | 0.03956398 | 0.090540942 |
| ENSG00000273749 | NONHSAT171364.1 | 0.041216391 | 0.09215222 | 0.038027343 | 0.116101283 |
| ENSG00000137845 | NONHSAT171625.1 | 0.069133474 | 0.185495338 | 0.127568777 | 0.292085834 |
| ENSG00000103657 | NONHSAT171681.1 | 0.066011909 | 0.295180818 | 0.121808699 | 0.37189415 |
| ENSG00000118898 | NONHSAT172595.1 | 0.528797529 | 1.576391176 | 0.976096647 | 2.730539993 |
| ENSG00000103429 | NONHSAT172702.1 | 0.072884117 | 1.238498176 | 0.134489663 | 6.569150382 |
| ENSG00000122257 | NONHSAT172788.1 | 1.817625873 | 3.814018682 | 1.032427304 | 3.248996954 |
| ENSG00000077238 | NONHSAT172814.1 | 0.048026056 | 0.171787455 | 0.088620242 | 0.405815874 |
| ENSG00000052344 | NONHSAT172877.1 | 0.155821216 | 0.766478765 | 0.143764813 | 2.194422291 |
| ENSG00000177508 | NONHSAT173898.1 | 0.113227717 | 1.012624855 | 0.627014247 | 1.674337132 |
| ENSG00000087253 | NONHSAT173914.1 | 0.072671005 | 0.389974117 | 0.268329392 | 0.614063493 |
| ENSG00000272617 | NONHSAT174001.1 | 0.282425953 | 0.613403795 | 0.148975084 | 0.852312153 |
| ENSG00000183011 | NONHSAT174834.1 | 0.112205345 | 0.602127079 | 0.207047292 | 0.948125122 |
| ENSG00000034152 | NONHSAT175005.1 | 0.247298347 | 0.66353805 | 0.456328112 | 1.741257954 |
| ENSG00000160551 | NONHSAT175062.1 | 0.150399297 | 0.302657673 | 0.069381203 | 0.211827757 |
| ENSG00000141577 | NONHSAT175735.1 | 0.120574816 | 0.431343 | 0.166924957 | 0.424491023 |
| ENSG00000033627 | NONHSAT176218.1 | 0.096145005 | 0.229298713 | 0.059137299 | 0.225656253 |
| ENSG00000198265 | NONHSAT176482.1 | 0.105985006 | 0.28437345 | 0.195569191 | 0.895563815 |
| ENSG00000017797 | NONHSAT177414.1 | 0.066187707 | 0.295966918 | 0.12213309 | 0.74567585 |
| ENSG00000101558 | NONHSAT177423.1 | 1.493342379 | 4.19754036 | 3.150454133 | 6.309304731 |
| ENSG00000141446 | NONHSAT178256.1 | 0.304018151 | 1.178212966 | 0.467682526 | 1.070385905 |
| ENSG00000046604 | NONHSAT178328.1 | 0.184784267 | 0.6609666 | 0.340973793 | 1.041027119 |
| ENSG00000127663 | NONHSAT179381.1 | 0.123895732 | 0.797791242 | 0.365930116 | 1.954181441 |
| ENSG00000099783 | NONHSAT179416.1 | 0.236137614 | 1.478348218 | 0.217866866 | 1.164004773 |
| ENSG00000105401 | NONHSAT179441.1 | 0.127945863 | 0.400477688 | 0.118046268 | 0.450441247 |
| ENSG00000166398 | NONHSAT179667.1 | 0.085114671 | 11.18980834 | 0.157058134 | 2.51723973 |
| ENSG00000105220 | NONHSAT179668.1 | 0.328460139 | 0.815973406 | 1.010565107 | 2.364245428 |
| ENSG00000168661 | NONHSAT179677.1 | 0.116273609 | 0.285972779 | 0.107331757 | 0.245625397 |
| ENSG00000167671 | NONHSAT180103.1 | 0.692511935 | 2.202119528 | 0.852195834 | 2.058846504 |
| ENSG00000105669 | NONHSAT180235.1 | 0.070108558 | 0.188111633 | 0.517735695 | 1.38225951 |
| ENSG00000089639 | NONHSAT180247.1 | 0.172354257 | 0.36993226 | 0.190887139 | 0.388351292 |
| ENSG00000239382 | NONHSAT180374.1 | 0.028699173 | 0.56468066 | 0.052957246 | 0.848769054 |
| ENSG00000115760 | NONHSAT181515.1 | 0.467673084 | 1.493757767 | 0.123282191 | 0.846813365 |
| ENSG00000124380 | NONHSAT181979.1 | 3.633454435 | 7.36583442 | 0.715454009 | 4.50306403 |
| ENSG00000114956 | NONHSAT184970.1 | 0.057464703 | 0.308372597 | 0.106036937 | 0.242785797 |
| ENSG00000135972 | NONHSAT185260.1 | 0.163241273 | 0.364977267 | 0.301374883 | 0.689687076 |
| ENSG00000121988 | NONHSAT185568.1 | 0.246074098 | 0.733568171 | 0.151356354 | 0.346550684 |
| ENSG00000153234 | NONHSAT185717.1 | 0.160086853 | 0.357924566 | 0.443251617 | 1.12719114 |
| ENSG00000168385 | NONHSAT186543.1 | 0.305754635 | 0.820347771 | 0.080599253 | 0.184542807 |
| ENSG00000101363 | NONHSAT188698.1 | 2.040755275 | 4.17175723 | 0.80721189 | 2.052743981 |
| ENSG00000124214 | NONHSAT189766.1 | 0.084679673 | 0.302896108 | 0.156255452 | 0.357767827 |
| ENSG00000205726 | NONHSAT190899.1 | 0.075542504 | 0.152018786 | 0.069733018 | 0.3191637 |
| ENSG00000182670 | NONHSAT190953.1 | 0.199626377 | 0.535593146 | 0.221091771 | 0.899602512 |
| ENSG00000205581 | NONHSAT191486.1 | 0.61493156 | 2.230384606 | 0.126142477 | 0.288673147 |
| ENSG00000279185 | NONHSAT192200.1 | 0.011931104 | 0.033790285 | 0.011011692 | 0.039205584 |
| ENSG00000134107 | NONHSAT193408.1 | 0.345187276 | 1.029033129 | 1.486883941 | 9.722509874 |
| ENSG00000131375 | NONHSAT193516.1 | 0.407434162 | 0.874495605 | 0.150363853 | 0.688556441 |
| ENSG00000144566 | NONHSAT193614.1 | 0.059960154 | 0.536239218 | 0.332037701 | 0.759986904 |
| ENSG00000163513 | NONHSAT193697.1 | 0.593769239 | 1.820694388 | 0.1565221 | 0.358378353 |
| ENSG00000179152 | NONHSAT193819.1 | 0.849865361 | 2.136463806 | 0.466411769 | 0.970437671 |
| ENSG00000136068 | NONHSAT193937.1 | 0.166244039 | 0.594648881 | 0.306762376 | 1.170544645 |
| ENSG00000144802 | NONHSAT194237.1 | 0.091289197 | 0.449048165 | 0.168537469 | 0.642778418 |
| ENSG00000242104 | NONHSAT194561.1 | 0.024302624 | 0.094184071 | 0.029911555 | 0.136903334 |
| ENSG00000129810 | NONHSAT195475.1 | 0.024402046 | 0.087285231 | 0.045027958 | 0.206195105 |
| ENSG00000173473 | NONHSAT195723.1 | 0.068561335 | 0.183960204 | 0.063256518 | 0.337962765 |
| ENSG00000173706 | NONHSAT196168.1 | 0.038924799 | 0.313322979 | 0.071826116 | 0.219292322 |
| ENSG00000118007 | NONHSAT196308.1 | 0.27247359 | 0.661437553 | 0.071826116 | 0.328911064 |
| ENSG00000114331 | NONHSAT196837.1 | 0.114006807 | 0.305897129 | 0.210371446 | 0.963347315 |
| ENSG00000087128 | NONHSAT198684.1 | 0.044983681 | 0.120697872 | 0.08300629 | 0.19005404 |
| ENSG00000145425 | NONHSAT199221.1 | 0.218973426 | 1.04447079 | 0.539022919 | 1.336263454 |
| ENSG00000109686 | NONHSAT199225.1 | 0.073913707 | 0.165257365 | 0.068194759 | 0.156141052 |
| ENSG00000151718 | NONHSAT199467.1 | 0.073639952 | 0.922051259 | 0.135884371 | 0.414868585 |
| ENSG00000198301 | NONHSAT200088.1 | 0.085260665 | 0.533741275 | 0.157327531 | 0.600330787 |
| ENSG00000138674 | NONHSAT200144.1 | 1.437280293 | 3.642094513 | 0.331637656 | 4.385688798 |
| ENSG00000129116 | NONHSAT200663.1 | 0.098429639 | 0.396151925 | 0.181720103 | 0.485194069 |
| ENSG00000145439 | NONHSAT200665.1 | 0.489167737 | 1.581667436 | 0.138938156 | 2.119669072 |
| ENSG00000145555 | NONHSAT202310.1 | 0.287656063 | 0.771774846 | 0.106159665 | 0.243066799 |
| ENSG00000164172 | NONHSAT202586.1 | 0.015562607 | 0.083513555 | 0.028716954 | 0.06575132 |
| ENSG00000113716 | NONHSAT203413.1 | 0.096331333 | 0.947700488 | 0.177755718 | 1.085276286 |
| ENSG00000177034 | NONHSAT204362.1 | 0.33360381 | 0.976430281 | 0.335886827 | 3.972032398 |
| ENSG00000124787 | NONHSAT206711.1 | 0.003478444 | 0.136879744 | 0.012843758 | 0.039188423 |
| ENSG00000222800 | NONHSAT207007.1 | 0.794774694 | 1.82506233 | 1.308573295 | 3.176404476 |
| ENSG00000206341 | NONHSAT207057.1 | 0.182522525 | 0.435302697 | 0.224647863 | 0.514099669 |
| ENSG00000137312 | NONHSAT207081.1 | 0.298003404 | 0.692941798 | 0.109978358 | 0.419655694 |
| ENSG00000156639 | NONHSAT207227.1 | 0.041735489 | 0.149286327 | 0.077012553 | 0.528991724 |
| ENSG00000024048 | NONHSAT207281.1 | 0.046849168 | 0.544687521 | 0.043224293 | 0.09896782 |
| ENSG00000078269 | NONHSAT208311.1 | 0.130980152 | 0.409975182 | 0.120845784 | 0.276692641 |
| ENSG00000137177 | NONHSAT208822.1 | 0.360849131 | 1.048844754 | 0.16646452 | 1.397481448 |
| ENSG00000132424 | NONHSAT209478.1 | 0.057866086 | 3.622629166 | 0.106777591 | 2.526255762 |
| ENSG00000196591 | NONHSAT209597.1 | 0.466732092 | 1.878466874 | 0.430619486 | 0.985961101 |
| ENSG00000105877 | NONHSAT211760.1 | 0.0864469 | 0.695812709 | 0.159597656 | 0.791300358 |
| ENSG00000006634 | NONHSAT212328.1 | 1.333137066 | 4.087842573 | 0.351425097 | 2.145603691 |
| ENSG00000105856 | NONHSAT212507.1 | 0.192166112 | 0.74473384 | 0.236517144 | 0.721652788 |
| ENSG00000272899 | NONHSAT212687.1 | 0.077788682 | 0.208718542 | 0.071769914 | 0.16432685 |
| ENSG00000164659 | NONHSAT213685.1 | 0.224918406 | 0.603489358 | 0.41503145 | 1.267132557 |
| ENSG00000106344 | NONHSAT213961.1 | 0.073639952 | 4.214957271 | 0.135884371 | 0.414868585 |
| ENSG00000278819 | NONHSAT214831.1 | 0.065061476 | 0.232722533 | 0.240232073 | 0.549763651 |
| ENSG00000156471 | NONHSAT216121.1 | 0.466294257 | 1.000829867 | 0.172086211 | 0.656646996 |
| ENSG00000104549 | NONHSAT216366.1 | 1.709028937 | 3.642672891 | 0.648690083 | 1.619589693 |
| ENSG00000254101 | NONHSAT216529.1 | 0.016207032 | 0.057971965 | 0.20943393 | 0.433653356 |
| ENSG00000121039 | NONHSAT217210.1 | 0.444616611 | 0.947343306 | 0.168990756 | 0.349911824 |
| ENSG00000255364 | NONHSAT217543.1 | 0.286908905 | 0.577364277 | 0.132354907 | 0.404092778 |
| ENSG00000179526 | NONHSAT217801.1 | 0.33315662 | 0.893851833 | 0.246028558 | 1.407571814 |
| ENSG00000165280 | NONHSAT219442.1 | 0.640003877 | 2.480315277 | 0.39365644 | 1.502115231 |
| ENSG00000207206 | NONHSAT219688.1 | 0.026369744 | 0.070753925 | 0.04865886 | 0.148560369 |
| ENSG00000130957 | NONHSAT219854.1 | 0.117141322 | 0.279373373 | 0.072051807 | 0.219981379 |
| ENSG00000119397 | NONHSAT220119.1 | 0.072459137 | 0.259183696 | 0.133705467 | 0.30613661 |
| ENSG00000137055 | NONHSAT220592.1 | 0.132199382 | 0.709383524 | 0.081313786 | 0.310277348 |
| ENSG00000107372 | NONHSAT220833.1 | 0.513501733 | 1.928834222 | 0.568717468 | 1.301713107 |
| ENSG00000136932 | NONHSAT221074.1 | 0.061518524 | 0.385112826 | 0.113517265 | 0.433159466 |
| ENSG00000160325 | NONHSAT221421.1 | 0.133800721 | 0.299153642 | 0.247021944 | 0.565302058 |
| ENSG00000224430 | NONHSAT222794.1 | 0.15131497 | 0.338312261 | 0.046535743 | 0.31964949 |
| ENSG00000147255 | NONHSAT223023.1 | 0.041630626 | 0.260612365 | 0.153716335 | 0.410423794 |
| ENSG00000068308 | NONHSAT223328.1 | 2.255343585 | 11.43013101 | 0.979653399 | 4.048146899 |
| ENSG00000270050 | NONHSAT223473.1 | 0.177604172 | 0.416952105 | 0.081972891 | 0.18759242 |
| ENSG00000102024 | NONHSAT223516.1 | 0.111052207 | 0.37741544 | 0.205002931 | 0.907091406 |
| ENSG00000121774 | NONHSAT224881.1 | 1.04346688 | 3.393533434 | 0.875565951 | 2.003909491 |
| ENSG00000116863 | NONHSAT224902.1 | 1.728268467 | 3.833374339 | 1.531474855 | 3.407635598 |
| ENSG00000117543 | NONHSAT225277.1 | 0.033676807 | 0.542130498 | 0.062142243 | 0.379405531 |
| ENSG00000228288 | NONHSAT225839.1 | 0.055683683 | 0.232406497 | 0.034250168 | 0.078420356 |
| ENSG00000187634 | NONHSAT226240.1 | 0.014520049 | 0.034629241 | 0.026802264 | 0.088607148 |
| ENSG00000187583 | NONHSAT226251.1 | 0.236699846 | 1.058434075 | 1.310758355 | 3.00013878 |
| ENSG00000233929 | NONHSAT226400.1 | 0.114006807 | 0.254898114 | 0.105185723 | 0.32114254 |
| ENSG00000157191 | NONHSAT226402.1 | 0.782896899 | 1.643304767 | 0.973332885 | 2.655180134 |
| ENSG00000121775 | NONHSAT226528.1 | 0.125931514 | 0.337877584 | 0.13285364 | 0.506710612 |
| ENSG00000172456 | NONHSAT226705.1 | 0.009059043 | 0.129630874 | 0.016716229 | 0.10205988 |
| ENSG00000122483 | NONHSAT226899.1 | 0.025884552 | 0.185198214 | 0.04777977 | 0.291629529 |
| ENSG00000188641 | NONHSAT226931.1 | 0.100316787 | 0.224289764 | 0.092554945 | 0.353171391 |
| ENSG00000235527 | NONHSAT227037.1 | 0.014824625 | 0.039776662 | 0.02735519 | 0.104382001 |
| ENSG00000160752 | NONHSAT227196.1 | 0.527123076 | 1.280447457 | 0.06006763 | 0.256617437 |
| ENSG00000132716 | NONHSAT227248.1 | 3.380450789 | 7.054577558 | 2.542355333 | 5.642519598 |
| ENSG00000272645 | NONHSAT227671.1 | 0.234466829 | 0.629109189 | 0.43265071 | 1.650909664 |
| ENSG00000177788 | NONHSAT227726.1 | 0.190567543 | 0.946223251 | 0.145575148 | 0.758877422 |
| ENSG00000235816 | NONHSAT228269.1 | 0.177524885 | 0.476325529 | 0.163789197 | 0.375017348 |
| ENSG00000171988 | NONHSAT229080.1 | 0.215414812 | 0.915119995 | 0.099373727 | 0.530927421 |
| ENSG00000223482 | NONHSAT229237.1 | 0.414280052 | 1.987302554 | 1.042863309 | 2.333755969 |
| ENSG00000213185 | NONHSAT229465.1 | 0.134798557 | 0.602769236 | 0.062184373 | 0.56951787 |
| ENSG00000179988 | NONHSAT229467.1 | 0.120355854 | 0.753441074 | 0.222087047 | 0.847440312 |
| ENSG00000165752 | NONHSAT229537.1 | 0.655116544 | 1.31826131 | 0.453474884 | 1.499168719 |
| ENSG00000176485 | NONHSAT229837.1 | 0.075466044 | 0.254971191 | 0.123828578 | 0.330641698 |
| ENSG00000175505 | NONHSAT229898.1 | 0.062840667 | 0.131129862 | 0.038652318 | 0.118009395 |
| ENSG00000205864 | NONHSAT230230.1 | 0.01524286 | 0.163582435 | 0.02812694 | 0.064400403 |
| ENSG00000255717 | NONHSAT230485.1 | 1.116960291 | 3.587284881 | 0.562327384 | 2.359588186 |
| ENSG00000133884 | NONHSAT230521.1 | 0.015113094 | 0.040550668 | 0.083690865 | 0.170265297 |
| ENSG00000174516 | NONHSAT230554.1 | 0.746223549 | 1.850252789 | 0.403185385 | 0.837327472 |
| ENSG00000283691 | NONHSAT230847.1 | 0.003619791 | 0.158628218 | 0.006679431 | 0.040780845 |
| ENSG00000149547 | NONHSAT230918.1 | 0.006165588 | 0.044113409 | 0.0341428 | 0.104197328 |
| ENSG00000151491 | NONHSAT231105.1 | 0.226314837 | 0.515201543 | 0.189899238 | 0.492562793 |
| ENSG00000135404 | NONHSAT231280.1 | 0.025016088 | 0.178984552 | 0.092369051 | 0.352282686 |
| ENSG00000139318 | NONHSAT231414.1 | 0.04226783 | 0.26460133 | 0.077994856 | 0.178579689 |
| ENSG00000122965 | NONHSAT231625.1 | 0.064764779 | 0.36200514 | 0.029876857 | 0.114004185 |
| ENSG00000255857 | NONHSAT231665.1 | 0.141112754 | 0.410158594 | 0.065097197 | 0.198748258 |
| ENSG00000174718 | NONHSAT231998.1 | 0.081353466 | 0.218283385 | 0.450506145 | 1.260249228 |
| ENSG00000150456 | NONHSAT232584.1 | 0.177145288 | 0.380215481 | 0.130817751 | 0.698518883 |
| ENSG00000170919 | NONHSAT232763.1 | 0.654295896 | 1.491274691 | 0.389636143 | 0.921466539 |
| ENSG00000136143 | NONHSAT232775.1 | 0.096733048 | 0.284252369 | 0.12754978 | 0.350308114 |
| ENSG00000165300 | NONHSAT232881.1 | 0.154406351 | 0.318668324 | 0.241174449 | 0.51853135 |
| ENSG00000136152 | NONHSAT233192.1 | 0.072213512 | 0.193759537 | 0.044417409 | 0.169488062 |
| ENSG00000136144 | NONHSAT233217.1 | 0.124267419 | 0.777927909 | 0.229304876 | 1.225115024 |
| ENSG00000134871 | NONHSAT233479.1 | 0.032984053 | 0.117982757 | 0.060863935 | 0.418068443 |
| ENSG00000176165 | NONHSAT234122.1 | 0.017938278 | 0.080213337 | 0.049667855 | 0.126305611 |
| ENSG00000104067 | NONHSAT234694.1 | 0.225122137 | 0.563754167 | 0.166247558 | 0.380452382 |
| ENSG00000137824 | NONHSAT234775.1 | 0.187573463 | 0.503287351 | 0.173060284 | 0.396244745 |
| ENSG00000128881 | NONHSAT234798.1 | 0.678026463 | 1.636315899 | 0.009929842 | 2.440256131 |
| ENSG00000140481 | NONHSAT234975.1 | 0.040974786 | 0.142511482 | 0.008400985 | 0.057705545 |
| ENSG00000185088 | NONHSAT235381.1 | 0.067080928 | 0.359976108 | 0.12378131 | 0.377916727 |
| ENSG00000179335 | NONHSAT235479.1 | 0.063459561 | 0.178374413 | 0.016728424 | 0.127664727 |
| ENSG00000178741 | NONHSAT235484.1 | 0.148379008 | 1.19436849 | 0.273796867 | 1.253789341 |
| ENSG00000171490 | NONHSAT235853.1 | 0.056118507 | 0.338780147 | 0.077691012 | 0.296372727 |
| ENSG00000091262 | NONHSAT235876.1 | 0.088015879 | 0.354220724 | 0.081247147 | 0.185931576 |
| ENSG00000247735 | NONHSAT235951.1 | 0.150285617 | 0.32761534 | 0.260088752 | 0.952411777 |
| ENSG00000273724 | NONHSAT235964.1 | 0.060930336 | 0.196171762 | 0.067482043 | 0.171607183 |
| ENSG00000270006 | NONHSAT236199.1 | 0.259792514 | 0.619585895 | 0.05326478 | 0.121956861 |
| ENSG00000205913 | NONHSAT236313.1 | 0.34787147 | 3.145718676 | 0.214042966 | 0.544312362 |
| ENSG00000188215 | NONHSAT236432.1 | 0.132551914 | 0.355656395 | 0.244591868 | 0.746763453 |
| ENSG00000103121 | NONHSAT236810.1 | 0.015365369 | 0.041227557 | 0.028352999 | 0.086564543 |
| ENSG00000161929 | NONHSAT236982.1 | 0.020478015 | 0.042731552 | 0.012595709 | 0.134581433 |
| ENSG00000157637 | NONHSAT237578.1 | 0.035066644 | 0.094088993 | 0.06473979 | 0.148155001 |
| ENSG00000072210 | NONHSAT237785.1 | 0.06003257 | 0.214759845 | 0.055387651 | 0.760904763 |
| ENSG00000131475 | NONHSAT237907.1 | 0.024619598 | 0.198174068 | 0.045429396 | 0.173349603 |
| ENSG00000108506 | NONHSAT238102.1 | 0.226455434 | 0.506312428 | 0.418080419 | 0.956764076 |
| ENSG00000215769 | NONHSAT238130.1 | 0.054924826 | 0.245603487 | 0.304153872 | 0.850842296 |
| ENSG00000134755 | NONHSAT238435.1 | 0.045353073 | 0.283915295 | 0.167461044 | 0.766458812 |
| ENSG00000252139 | NONHSAT238522.1 | 0.096954094 | 0.215379774 | 0.011548311 | 0.244454143 |
| ENSG00000264745 | NONHSAT238791.1 | 0.217535964 | 0.680899701 | 0.200704487 | 1.072310743 |
| ENSG00000267140 | NONHSAT238829.1 | 0.053034908 | 0.106725379 | 0.048956336 | 0.112035057 |
| ENSG00000006638 | NONHSAT239057.1 | 0.020083623 | 0.125725723 | 0.037059374 | 0.25455723 |
| ENSG00000105771 | NONHSAT239418.1 | 0.737309781 | 1.593569922 | 0.4537387 | 2.07673389 |
| ENSG00000182310 | NONHSAT239517.1 | 0.003763114 | 0.016827251 | 0.006943898 | 0.026496544 |
| ENSG00000198131 | NONHSAT239585.1 | 0.172369199 | 0.443197127 | 0.119314805 | 0.273094557 |
| ENSG00000174586 | NONHSAT239590.1 | 0.109910376 | 0.35632893 | 0.05072894 | 0.116091606 |
| ENSG00000283269 | NONHSAT239900.1 | 0.00608334 | 0.013601221 | 0.011231018 | 0.064254594 |
| ENSG00000171103 | NONHSAT240464.1 | 0.442823766 | 1.188067526 | 0.204280513 | 0.779494095 |
| ENSG00000162961 | NONHSAT240479.1 | 0.054653071 | 1.148643586 | 0.252224606 | 0.846632521 |
| ENSG00000270820 | NONHSAT240629.1 | 0.064694535 | 1.330751152 | 0.09288993 | 0.425172231 |
| ENSG00000003137 | NONHSAT240705.1 | 0.110953053 | 0.893110366 | 0.409681482 | 1.0938527 |
| ENSG00000005436 | NONHSAT240758.1 | 0.516244472 | 1.319116652 | 0.68070706 | 1.454038565 |
| ENSG00000125629 | NONHSAT240971.1 | 0.849971072 | 2.544988563 | 2.524178298 | 6.765973404 |
| ENSG00000163166 | NONHSAT241015.1 | 0.035178321 | 0.09438864 | 0.064912916 | 0.148626833 |
| ENSG00000152127 | NONHSAT241065.1 | 0.030588903 | 0.082074553 | 0.112946033 | 0.258473495 |
| ENSG00000138398 | NONHSAT241219.1 | 0.07126447 | 3.138925413 | 0.098659231 | 0.32616323 |
| ENSG00000115415 | NONHSAT241310.1 | 0.165689893 | 0.370451926 | 0.305895507 | 0.816743349 |
| ENSG00000138413 | NONHSAT241385.1 | 0.052823558 | 0.110227121 | 0.032490949 | 0.074392389 |
| ENSG00000085978 | NONHSAT241550.1 | 0.236699846 | 1.058434075 | 0.436993581 | 1.333339437 |
| ENSG00000115295 | NONHSAT241835.1 | 5.123051484 | 11.50918589 | 0.562940563 | 1.374239419 |
| ENSG00000116016 | NONHSAT241952.1 | 0.023920581 | 0.100852336 | 0.056770092 | 0.115501012 |
| ENSG00000143429 | NONHSAT242306.1 | 0.17319501 | 0.464707833 | 0.159794339 | 0.975614222 |
| ENSG00000241074 | NONHSAT242694.1 | 0.007363995 | 0.03292906 | 0.013588437 | 0.062225101 |
| ENSG00000082153 | NONHSAT242883.1 | 0.629733544 | 1.464307752 | 0.464964957 | 1.005094347 |
| ENSG00000087460 | NONHSAT243538.1 | 0.407434162 | 1.457550426 | 0.376101033 | 1.434396921 |
| ENSG00000125534 | NONHSAT243579.1 | 0.564851906 | 1.414510454 | 0.417130236 | 0.954589612 |
| ENSG00000125508 | NONHSAT243584.1 | 0.053477104 | 0.239129807 | 0.049339403 | 0.301238603 |
| ENSG00000125846 | NONHSAT243692.1 | 0.170931801 | 0.550333298 | 0.189311729 | 0.4814207 |
| ENSG00000159267 | NONHSAT244252.1 | 0.025347765 | 0.158687024 | 0.046788926 | 0.095189965 |
| ENSG00000185837 | NONHSAT244693.1 | 0.343718394 | 0.827627853 | 0.048788271 | 0.148955476 |
| ENSG00000073169 | NONHSAT244981.1 | 0.90376305 | 2.065567214 | 0.18529687 | 0.989991939 |
| ENSG00000168137 | NONHSAT245358.1 | 0.42124549 | 1.036045449 | 0.194326166 | 0.593297235 |
| ENSG00000181322 | NONHSAT245889.1 | 0.007960757 | 0.021359889 | 0.014689614 | 0.089686509 |
| ENSG00000241313 | NONHSAT245935.1 | 0.097601339 | 0.261866974 | 0.077211572 | 0.589067916 |
| ENSG00000163645 | NONHSAT245945.1 | 0.010438254 | 0.084022143 | 0.019261224 | 0.044101158 |
| ENSG00000154781 | NONHSAT246330.1 | 0.023260163 | 0.062410458 | 0.042920894 | 0.163777655 |
| ENSG00000170248 | NONHSAT246390.1 | 0.030567405 | 0.316335931 | 0.032247616 | 0.116842207 |
| ENSG00000164080 | NONHSAT246505.1 | 0.013108378 | 0.679951213 | 0.012094139 | 0.036924617 |
| ENSG00000144843 | NONHSAT246748.1 | 0.037886408 | 0.118586571 | 0.034955012 | 0.106721149 |
| ENSG00000132406 | NONHSAT247253.1 | 0.025701638 | 0.689569253 | 0.047426034 | 0.21717654 |
| ENSG00000249348 | NONHSAT247473.1 | 0.015182336 | 0.081472907 | 0.028015257 | 0.085533383 |
| ENSG00000251445 | NONHSAT247909.1 | 0.122130142 | 0.819181164 | 0.112680529 | 0.257997192 |
| ENSG00000145246 | NONHSAT248274.1 | 0.554307819 | 1.213723691 | 0.035288849 | 0.619131373 |
| ENSG00000245213 | NONHSAT248759.1 | 0.078360958 | 0.163516113 | 0.144644905 | 0.294273549 |
| ENSG00000039123 | NONHSAT249133.1 | 0.376567938 | 0.841936196 | 0.34743163 | 0.795491343 |
| ENSG00000215196 | NONHSAT249916.1 | 0.065260789 | 0.36965241 | 0.040140897 | 0.214462149 |
| ENSG00000113407 | NONHSAT249954.1 | 0.09074723 | 0.43532716 | 0.083760587 | 0.214943828 |
| ENSG00000229666 | NONHSAT250089.1 | 0.154729861 | 0.328659827 | 0.142830587 | 0.490295054 |
| ENSG00000250343 | NONHSAT250512.1 | 0.0175457 | 0.052305249 | 0.010792087 | 0.065890406 |
| ENSG00000246334 | NONHSAT250632.1 | 0.225567183 | 1.557415481 | 0.116588702 | 0.67981967 |
| ENSG00000158406 | NONHSAT250932.1 | 0.431984077 | 1.725647338 | 0.85058611 | 2.230667101 |
| ENSG00000276180 | NONHSAT250955.1 | 0.443812212 | 1.190813822 | 0.819362964 | 1.875086738 |
| ENSG00000204110 | NONHSAT251114.1 | 0.075401468 | 0.191930032 | 0.032118933 | 0.065344523 |
| ENSG00000112697 | NONHSAT251225.1 | 0.110541811 | 0.428401379 | 0.06799255 | 0.207588062 |
| ENSG00000146247 | NONHSAT251233.1 | 0.084363489 | 0.663955036 | 0.031134403 | 0.190089125 |
| ENSG00000228439 | NONHSAT251347.1 | 0.146758098 | 0.433132335 | 0.04063467 | 0.299685646 |
| ENSG00000135540 | NONHSAT251574.1 | 0.101650241 | 1.363648335 | 0.093832978 | 1.252602247 |
| ENSG00000273132 | NONHSAT251648.1 | 0.056097396 | 0.380456524 | 0.172593458 | 0.49376214 |
| ENSG00000029639 | NONHSAT251690.1 | 0.160994228 | 1.007876054 | 0.074268786 | 0.226750036 |
| ENSG00000137210 | NONHSAT251949.1 | 0.032048335 | 0.300952857 | 0.088736059 | 0.180529518 |
| ENSG00000008083 | NONHSAT252011.1 | 0.072600245 | 0.238078604 | 0.357378682 | 0.749768528 |
| ENSG00000112242 | NONHSAT252030.1 | 0.463252262 | 0.952894475 | 0.17105061 | 0.391444016 |
| ENSG00000204623 | NONHSAT252144.1 | 0.003976876 | 0.103144097 | 0.036706661 | 0.151219092 |
| ENSG00000124596 | NONHSAT252278.1 | 0.062603234 | 0.447912221 | 0.115518829 | 0.793487587 |
| ENSG00000234707 | NONHSAT253385.1 | 0.021589981 | 0.066324562 | 0.064116255 | 0.220777273 |
| ENSG00000164654 | NONHSAT253916.1 | 0.023216706 | 0.093440786 | 0.021420353 | 0.049044772 |
| ENSG00000271133 | NONHSAT253970.1 | 0.161911947 | 0.651650561 | 1.344914924 | 4.788381072 |
| ENSG00000196295 | NONHSAT254047.1 | 0.024188305 | 0.186037995 | 0.017862511 | 0.08856403 |
| ENSG00000146674 | NONHSAT254152.1 | 0.784330852 | 3.594951453 | 0.180911145 | 0.552339832 |
| ENSG00000135250 | NONHSAT254388.1 | 0.058273116 | 0.138977063 | 0.035842888 | 0.109431925 |
| ENSG00000243797 | NONHSAT254406.1 | 0.106713112 | 0.286308917 | 0.039382546 | 0.120238856 |
| ENSG00000197157 | NONHSAT254482.1 | 0.108294047 | 0.329286917 | 0.039965991 | 0.091507501 |
| ENSG00000157110 | NONHSAT254968.1 | 0.141212977 | 0.378895307 | 0.260573723 | 0.596618507 |
| ENSG00000207816 | NONHSAT255123.1 | 0.002027036 | 0.012689469 | 0.003740394 | 0.008564135 |
| ENSG00000147459 | NONHSAT255778.1 | 0.058238978 | 0.240896364 | 0.067193403 | 0.225545483 |
| ENSG00000169946 | NONHSAT256174.1 | 0.246063034 | 1.010336753 | 0.516197748 | 1.118392562 |
| ENSG00000279518 | NONHSAT256325.1 | 0.440817265 | 1.02766508 | 0.026997893 | 0.5590265 |
| ENSG00000235453 | NONHSAT256571.1 | 0.034931109 | 0.249924318 | 0.064456747 | 0.836283702 |
| ENSG00000230074 | NONHSAT256603.1 | 0.01539392 | 0.096367657 | 0.056840292 | 0.216780953 |
| ENSG00000095383 | NONHSAT256949.1 | 0.242354792 | 0.606887824 | 0.17897348 | 0.580220238 |
| ENSG00000182150 | NONHSAT257642.1 | 0.022506389 | 1.081178473 | 0.017804609 | 1.263295817 |
| ENSG00000232593 | NONHSAT258160.1 | 0.080796186 | 0.206456696 | 0.170452993 | 0.373865352 |
| ENSG00000197021 | NONHSAT258452.1 | 0.008524604 | 0.022872774 | 0.015730055 | 0.036016072 |

**Supplemental table S4. The ectopically expressed lncRNAs in the cohort of ESCC from TCGA.**

| gene | logFC | logCPM | PValue | FDR |  |  |
| --- | --- | --- | --- | --- | --- | --- |
| ENSG00000255477.1 | 8.097515 | 1.579279 | 0.04103 | 0.084215 | AC021713.1 | lincRNA |
| ENSG00000225107.1 | 7.909834 | 0.886633 | 0.010707 | 0.027788 | AC092484.1 | lincRNA |
| ENSG00000228295.1 | 7.89855 | 1.877651 | 2.04E-05 | 0.000129 | LINC00392 | lincRNA |
| ENSG00000269993.1 | 7.638635 | -0.76103 | 9.05E-07 | 8.20E-06 | KC877982.1 | lincRNA |
| ENSG00000235385.1 | 7.140158 | 1.83848 | 6.20E-06 | 4.52E-05 | LINC02154 | lincRNA |
| ENSG00000254337.1 | 6.910592 | -0.38804 | 0.007601 | 0.020887 | AC083967.1 | lincRNA |
| ENSG00000266602.1 | 6.513876 | -1.9399 | 0.000835 | 0.00321 | AC008109.1 | lincRNA |
| ENSG00000249628.2 | 6.3864 | 3.79236 | 2.42E-05 | 0.00015 | LINC00942 | lincRNA |
| ENSG00000225548.4 | 6.151939 | 2.303698 | 7.02E-14 | 2.00E-12 | LINC01980 | lincRNA |
| ENSG00000242512.7 | 6.043417 | 5.328934 | 0.000725 | 0.002859 | LINC01206 | lincRNA |
| ENSG00000276399.1 | 5.610636 | -2.20629 | 0.007127 | 0.019828 | FLJ36000 | lincRNA |
| ENSG00000253339.1 | 5.60735 | -0.10354 | 0.010235 | 0.026783 | AC111149.2 | lincRNA |
| ENSG00000257253.2 | 5.393769 | -0.817 | 1.73E-07 | 1.83E-06 | AC131157.1 | lincRNA |
| ENSG00000275216.1 | 5.389128 | 5.991381 | 8.82E-08 | 9.97E-07 | AL161431.1 | lincRNA |
| ENSG00000258710.6 | 5.325902 | -2.56 | 0.022043 | 0.050494 | LINC01193 | lincRNA |
| ENSG00000224034.1 | 5.245362 | 0.367683 | 1.39E-07 | 1.51E-06 | LINC02561 | lincRNA |
| ENSG00000242147.1 | 5.218965 | 0.899666 | 2.59E-10 | 4.63E-09 | AL365356.5 | lincRNA |
| ENSG00000248359.1 | 5.097864 | -1.17393 | 0.000201 | 0.000949 | AC010280.1 | lincRNA |
| ENSG00000231131.5 | 5.080275 | 1.205456 | 3.33E-06 | 2.62E-05 | LNCAROD | lincRNA |
| ENSG00000205628.2 | 5.069496 | -0.87891 | 0.007002 | 0.01955 | LINC01446 | lincRNA |
| ENSG00000231107.1 | 5.066067 | -0.23428 | 7.87E-05 | 0.000421 | LINC01508 | lincRNA |
| ENSG00000255267.3 | 5.063087 | -0.95637 | 6.31E-07 | 5.91E-06 | AC018716.2 | lincRNA |
| ENSG00000261327.4 | 5.02314 | -0.26081 | 3.70E-13 | 9.81E-12 | AC134312.5 | lincRNA |
| ENSG00000198054.10 | 5.007825 | -1.40711 | 0.037847 | 0.078938 | DSCR8 | lincRNA |
| ENSG00000270816.4 | 4.997601 | -0.973 | 0.0441 | 0.089346 | LINC00221 | lincRNA |
| ENSG00000231971.4 | 4.973188 | 2.029986 | 9.22E-07 | 8.35E-06 | AL078590.2 | lincRNA |
| ENSG00000255426.1 | 4.904043 | -2.87413 | 0.007355 | 0.020337 | AC044839.3 | lincRNA |
| ENSG00000229205.2 | 4.901745 | -2.51954 | 0.022083 | 0.050557 | LINC00200 | lincRNA |
| ENSG00000230838.1 | 4.853157 | 0.361152 | 4.18E-08 | 5.06E-07 | LINC01614 | lincRNA |
| ENSG00000267284.1 | 4.827796 | 0.459494 | 1.13E-14 | 3.55E-13 | AC022031.2 | lincRNA |
| ENSG00000231532.4 | 4.798153 | -0.62439 | 0.000793 | 0.003074 | LINC01249 | lincRNA |
| ENSG00000232243.1 | 4.790696 | -1.48807 | 1.54E-05 | 0.0001 | AL139327.2 | lincRNA |
| ENSG00000250564.1 | 4.786121 | 0.490462 | 4.28E-06 | 3.25E-05 | AC109454.3 | lincRNA |
| ENSG00000232316.1 | 4.770402 | -1.60602 | 7.63E-05 | 0.00041 | LINC02518 | lincRNA |
| ENSG00000240095.1 | 4.760623 | -1.33123 | 0.012443 | 0.031511 | AC055758.1 | lincRNA |
| ENSG00000278698.1 | 4.759021 | -2.3188 | 0.000123 | 0.000623 | AL162574.2 | lincRNA |
| ENSG00000261082.1 | 4.723926 | -2.91242 | 0.00832 | 0.02251 | LINC01228 | lincRNA |
| ENSG00000244358.1 | 4.71936 | -0.80991 | 0.006775 | 0.019023 | AC055758.2 | lincRNA |
| ENSG00000260289.1 | 4.706282 | -3.02597 | 0.004607 | 0.01378 | AC093515.1 | lincRNA |
| ENSG00000250284.1 | 4.689344 | -3.04964 | 0.006283 | 0.017844 | AC109439.2 | lincRNA |
| ENSG00000278626.1 | 4.644833 | -0.63813 | 0.000506 | 0.002105 | AC023310.4 | lincRNA |
| ENSG00000234862.1 | 4.640108 | -1.50493 | 0.00047 | 0.001969 | AL035258.1 | lincRNA |
| ENSG00000251434.1 | 4.635112 | -3.51725 | 0.010073 | 0.02643 | AC104071.1 | lincRNA |
| ENSG00000253659.1 | 4.614052 | -2.193 | 0.026711 | 0.059316 | AC138646.1 | lincRNA |
| ENSG00000250122.1 | 4.517194 | -2.13314 | 0.000524 | 0.002171 | AC122694.1 | lincRNA |
| ENSG00000233085.4 | 4.511776 | -2.21238 | 2.84E-05 | 0.000173 | BX322234.2 | lincRNA |
| ENSG00000233532.4 | 4.491247 | 0.373503 | 1.96E-06 | 1.63E-05 | LINC00460 | lincRNA |
| ENSG00000250920.1 | 4.486382 | 3.492815 | 0.001825 | 0.00626 | AC105460.1 | lincRNA |
| ENSG00000224853.1 | 4.475454 | -0.82611 | 0.000193 | 0.000921 | LINC00393 | lincRNA |
| ENSG00000253784.1 | 4.467246 | -2.7442 | 0.000805 | 0.003113 | AC090735.1 | lincRNA |
| ENSG00000232023.2 | 4.461158 | 0.096972 | 4.20E-05 | 0.000244 | LINC01807 | lincRNA |
| ENSG00000230387.2 | 4.453207 | 1.271447 | 1.57E-06 | 1.34E-05 | AL118508.1 | lincRNA |
| ENSG00000254885.1 | 4.447713 | -1.81884 | 0.002443 | 0.008031 | AP001547.1 | lincRNA |
| ENSG00000272666.1 | 4.44575 | 1.735174 | 1.97E-11 | 4.18E-10 | U62317.1 | lincRNA |
| ENSG00000248370.1 | 4.414248 | -1.85266 | 0.003489 | 0.010868 | LINC02434 | lincRNA |
| ENSG00000224141.4 | 4.413284 | -0.81116 | 0.011151 | 0.028731 | MIR548XHG | lincRNA |
| ENSG00000249550.5 | 4.388751 | 1.488864 | 0.000109 | 0.00056 | LINC01234 | lincRNA |
| ENSG00000280081.2 | 4.381983 | -0.10874 | 0.009218 | 0.024531 | LINC01667 | lincRNA |
| ENSG00000249500.1 | 4.356963 | -1.91031 | 0.000768 | 0.002998 | LINC01179 | lincRNA |
| ENSG00000235180.1 | 4.297194 | -2.84185 | 0.001807 | 0.006214 | LINC00601 | lincRNA |
| ENSG00000229647.1 | 4.279713 | 1.891505 | 1.08E-09 | 1.74E-08 | MYOSLID | lincRNA |
| ENSG00000248588.1 | 4.272574 | -2.88554 | 0.020063 | 0.04671 | AC008517.1 | lincRNA |
| ENSG00000231291.3 | 4.250947 | -1.15004 | 2.13E-06 | 1.76E-05 | AC106799.1 | lincRNA |
| ENSG00000250509.1 | 4.248252 | -1.43536 | 3.28E-05 | 0.000197 | AC034213.1 | lincRNA |
| ENSG00000237445.2 | 4.235437 | -0.3662 | 3.31E-05 | 0.000198 | AL359771.1 | lincRNA |
| ENSG00000233515.1 | 4.231157 | -1.11355 | 0.001288 | 0.004645 | LINC01518 | lincRNA |
| ENSG00000204460.3 | 4.20694 | -1.72767 | 0.026643 | 0.059186 | LINC01854 | lincRNA |
| ENSG00000223485.1 | 4.182295 | 1.670409 | 5.48E-06 | 4.06E-05 | LINC01615 | lincRNA |
| ENSG00000228742.8 | 4.175128 | 1.873442 | 5.75E-06 | 4.24E-05 | LINC02577 | lincRNA |
| ENSG00000248973.1 | 4.146102 | -0.62449 | 1.93E-06 | 1.61E-05 | AC106799.2 | lincRNA |
| ENSG00000267151.3 | 4.128033 | -0.31008 | 2.63E-06 | 2.12E-05 | MIR2117HG | lincRNA |
| ENSG00000272046.1 | 4.124631 | -1.97168 | 0.01698 | 0.040726 | AL445647.1 | lincRNA |
| ENSG00000260763.1 | 4.124369 | -0.07451 | 5.19E-06 | 3.87E-05 | AC106799.3 | lincRNA |
| ENSG00000267013.4 | 4.112251 | 0.019779 | 7.58E-06 | 5.41E-05 | LINC01929 | lincRNA |
| ENSG00000271252.1 | 4.105294 | -1.38272 | 2.57E-08 | 3.25E-07 | AL683887.1 | lincRNA |
| ENSG00000272071.1 | 4.097128 | -2.43887 | 2.51E-06 | 2.03E-05 | AC122710.3 | lincRNA |
| ENSG00000234722.3 | 4.091471 | 0.530243 | 0.003815 | 0.011747 | LINC01287 | lincRNA |
| ENSG00000272218.1 | 4.079675 | -1.95607 | 0.00912 | 0.024313 | AC108865.2 | lincRNA |
| ENSG00000248740.4 | 4.063008 | 0.520732 | 5.16E-09 | 7.33E-08 | LINC02428 | lincRNA |
| ENSG00000258028.1 | 4.047325 | -3.27466 | 0.0325 | 0.069698 | AL138974.1 | lincRNA |
| ENSG00000250102.4 | 4.03869 | -1.46744 | 0.014844 | 0.03649 | LINC02377 | lincRNA |
| ENSG00000234692.1 | 4.031084 | -2.65874 | 1.84E-05 | 0.000118 | AL355601.1 | lincRNA |
| ENSG00000269927.1 | 4.026074 | -1.40758 | 4.94E-06 | 3.70E-05 | AC004817.3 | lincRNA |
| ENSG00000225826.1 | 4.021785 | -1.49071 | 0.000248 | 0.001138 | LINC00626 | lincRNA |
| ENSG00000256268.1 | 4.012371 | -0.86434 | 1.12E-05 | 7.64E-05 | LINC02454 | lincRNA |
| ENSG00000250829.2 | 4.010914 | -1.20895 | 0.0024 | 0.007908 | AC108865.1 | lincRNA |
| ENSG00000253658.4 | 3.992105 | -2.11316 | 5.72E-06 | 4.21E-05 | LINC01592 | lincRNA |
| ENSG00000258815.1 | 3.987506 | -0.70048 | 2.37E-07 | 2.45E-06 | AC131532.1 | lincRNA |
| ENSG00000238266.1 | 3.973753 | 2.825922 | 0.000139 | 0.000693 | LINC00707 | lincRNA |
| ENSG00000231560.1 | 3.945847 | -0.11843 | 5.48E-06 | 4.06E-05 | CLEC12A-AS1 | lincRNA |
| ENSG00000254863.1 | 3.941668 | -2.91156 | 0.030794 | 0.066669 | AP001360.1 | lincRNA |
| ENSG00000260454.1 | 3.934109 | -1.7204 | 0.00026 | 0.001187 | AL355607.2 | lincRNA |
| ENSG00000253768.1 | 3.928232 | -1.65616 | 0.002888 | 0.009258 | AC008663.1 | lincRNA |
| ENSG00000260958.2 | 3.921871 | -2.2525 | 0.017841 | 0.042394 | AC023824.3 | lincRNA |
| ENSG00000251281.1 | 3.920519 | -1.05273 | 0.002044 | 0.006903 | AC034223.2 | lincRNA |
| ENSG00000267413.1 | 3.905689 | -1.54852 | 0.00101 | 0.003772 | LINC01901 | lincRNA |
| ENSG00000258955.1 | 3.899181 | 1.771367 | 3.33E-07 | 3.32E-06 | LINC00519 | lincRNA |
| ENSG00000264464.1 | 3.85958 | -0.27052 | 0.00075 | 0.002946 | AC091170.1 | lincRNA |
| ENSG00000250682.4 | 3.841611 | 1.073951 | 5.72E-08 | 6.72E-07 | LINC00491 | lincRNA |
| ENSG00000225087.1 | 3.83605 | -0.41802 | 4.79E-06 | 3.60E-05 | AL583808.1 | lincRNA |
| ENSG00000260976.1 | 3.826336 | -1.77464 | 4.21E-10 | 7.23E-09 | LINC01633 | lincRNA |
| ENSG00000241475.1 | 3.809605 | -2.39324 | 4.05E-05 | 0.000236 | AL160408.4 | lincRNA |
| ENSG00000254349.4 | 3.806266 | -0.53912 | 6.06E-12 | 1.38E-10 | MIR2052HG | lincRNA |
| ENSG00000230533.1 | 3.797145 | -1.11131 | 4.58E-06 | 3.46E-05 | AL356234.2 | lincRNA |
| ENSG00000265980.1 | 3.787904 | -3.60713 | 0.047569 | 0.095023 | AC074237.1 | lincRNA |
| ENSG00000231648.1 | 3.758084 | -1.94899 | 1.46E-07 | 1.58E-06 | LINC01698 | lincRNA |
| ENSG00000223784.1 | 3.755478 | 1.064548 | 0.000128 | 0.000646 | LINP1 | lincRNA |
| ENSG00000253898.1 | 3.754642 | -2.95447 | 0.001374 | 0.004907 | LINC01419 | lincRNA |
| ENSG00000228951.1 | 3.724003 | 0.138465 | 0.000482 | 0.002014 | AL365356.3 | lincRNA |
| ENSG00000248870.1 | 3.72062 | -2.01673 | 9.81E-08 | 1.10E-06 | AC026782.2 | lincRNA |
| ENSG00000248131.4 | 3.675692 | -0.92921 | 0.03057 | 0.066272 | LINC01194 | lincRNA |
| ENSG00000267123.4 | 3.675628 | 0.052125 | 4.87E-06 | 3.66E-05 | LINC02081 | lincRNA |
| ENSG00000248112.1 | 3.674365 | -1.79013 | 0.001341 | 0.00481 | AC108174.1 | lincRNA |
| ENSG00000254651.1 | 3.623538 | -1.99141 | 0.000376 | 0.001631 | AC018716.1 | lincRNA |
| ENSG00000205293.3 | 3.616804 | -2.77882 | 0.021245 | 0.048954 | LINC01602 | lincRNA |
| ENSG00000231574.4 | 3.611922 | -0.03784 | 0.000192 | 0.000914 | LINC02015 | lincRNA |
| ENSG00000264265.1 | 3.604433 | -2.78989 | 0.016795 | 0.040339 | LINC01925 | lincRNA |
| ENSG00000235126.1 | 3.579775 | -1.09656 | 0.000136 | 0.000679 | AC128709.2 | lincRNA |
| ENSG00000268621.4 | 3.573947 | 3.268374 | 0.000326 | 0.001441 | IGFL2-AS1 | lincRNA |
| ENSG00000251629.5 | 3.567985 | -0.73041 | 0.012818 | 0.032297 | LINC02241 | lincRNA |
| ENSG00000256151.1 | 3.529698 | 0.054714 | 0.006147 | 0.01752 | ADGRD1-AS1 | lincRNA |
| ENSG00000227674.1 | 3.518689 | 0.044957 | 0.002077 | 0.006993 | LINC00355 | lincRNA |
| ENSG00000205634.5 | 3.516234 | -0.48594 | 8.50E-06 | 6.00E-05 | LINC00898 | lincRNA |
| ENSG00000267057.4 | 3.501005 | -1.20297 | 9.53E-08 | 1.07E-06 | LINC01905 | lincRNA |
| ENSG00000248238.1 | 3.497068 | -2.57571 | 0.000201 | 0.000951 | LINC02438 | lincRNA |
| ENSG00000280356.1 | 3.49008 | 0.354483 | 0.00088 | 0.003356 | AL021877.2 | lincRNA |
| ENSG00000230990.1 | 3.477459 | -2.13384 | 0.000398 | 0.001712 | AL049649.1 | lincRNA |
| ENSG00000225882.1 | 3.435854 | -2.29243 | 0.000902 | 0.00343 | LINC01456 | lincRNA |
| ENSG00000206129.3 | 3.434373 | 0.933098 | 1.10E-09 | 1.75E-08 | AC006305.1 | lincRNA |
| ENSG00000267313.5 | 3.42894 | 1.550163 | 1.10E-05 | 7.52E-05 | KC6 | lincRNA |
| ENSG00000255980.1 | 3.420763 | -2.80722 | 0.008436 | 0.022768 | AP000439.3 | lincRNA |
| ENSG00000257114.2 | 3.420711 | -1.44293 | 4.60E-05 | 0.000264 | LINC02450 | lincRNA |
| ENSG00000249199.1 | 3.415686 | -1.92692 | 0.002086 | 0.007016 | AC026785.3 | lincRNA |
| ENSG00000236700.4 | 3.390525 | 0.759924 | 3.98E-06 | 3.06E-05 | LINC01010 | lincRNA |
| ENSG00000261706.1 | 3.358083 | -0.28551 | 0.046756 | 0.093679 | LINC00165 | lincRNA |
| ENSG00000281327.1 | 3.342661 | -2.52871 | 1.41E-05 | 9.33E-05 | LINC01338 | lincRNA |
| ENSG00000275874.1 | 3.336489 | 0.47546 | 0.006141 | 0.017509 | PICSAR | lincRNA |
| ENSG00000228189.4 | 3.321822 | -2.25781 | 0.034233 | 0.07274 | AL592486.1 | lincRNA |
| ENSG00000250874.1 | 3.309573 | -0.33179 | 3.20E-05 | 0.000192 | AC010595.1 | lincRNA |
| ENSG00000263745.4 | 3.309222 | 0.209181 | 3.77E-08 | 4.60E-07 | AP005230.1 | lincRNA |
| ENSG00000249069.6 | 3.303497 | -1.51412 | 0.018133 | 0.04298 | LINC01033 | lincRNA |
| ENSG00000267409.1 | 3.299842 | -2.50673 | 0.000113 | 0.000577 | AC068473.4 | lincRNA |
| ENSG00000250519.5 | 3.236434 | -1.46975 | 7.60E-06 | 5.42E-05 | AP002784.1 | lincRNA |
| ENSG00000258548.4 | 3.21861 | -2.35316 | 2.18E-05 | 0.000137 | LINC00645 | lincRNA |
| ENSG00000226363.3 | 3.200531 | 0.531176 | 1.23E-08 | 1.64E-07 | HAGLROS | lincRNA |
| ENSG00000237167.1 | 3.197034 | -1.35287 | 0.001011 | 0.003776 | AC128709.3 | lincRNA |
| ENSG00000258711.2 | 3.195793 | 1.220111 | 4.01E-07 | 3.93E-06 | AL358334.2 | lincRNA |
| ENSG00000250735.4 | 3.161025 | -2.3724 | 0.000659 | 0.002639 | AC096759.2 | lincRNA |
| ENSG00000275830.1 | 3.130824 | -1.17068 | 0.00165 | 0.005758 | AL355974.2 | lincRNA |
| ENSG00000269994.1 | 3.118723 | 2.601518 | 2.95E-07 | 2.98E-06 | AL513318.2 | lincRNA |
| ENSG00000259341.1 | 3.106321 | -0.84704 | 5.45E-05 | 0.000306 | AC015660.1 | lincRNA |
| ENSG00000247402.2 | 3.103799 | -1.81672 | 2.41E-06 | 1.96E-05 | AC099487.1 | lincRNA |
| ENSG00000229243.1 | 3.092164 | -2.10987 | 3.87E-06 | 2.98E-05 | LINC01981 | lincRNA |
| ENSG00000243961.2 | 3.069348 | -2.9588 | 0.002298 | 0.007622 | PARAL1 | lincRNA |
| ENSG00000258018.1 | 3.067775 | -1.03201 | 0.000209 | 0.000984 | LINC02457 | lincRNA |
| ENSG00000281406.1 | 3.061812 | 2.471592 | 3.27E-07 | 3.27E-06 | BLACAT1 | lincRNA |
| ENSG00000228793.1 | 3.061487 | -1.58198 | 0.004987 | 0.014735 | AL138881.1 | lincRNA |
| ENSG00000267327.1 | 3.055847 | 0.099874 | 7.53E-07 | 6.94E-06 | AC009271.1 | lincRNA |
| ENSG00000254605.1 | 3.052294 | 1.582665 | 0.004047 | 0.01235 | AP003555.2 | lincRNA |
| ENSG00000276850.3 | 3.052101 | 3.605276 | 2.89E-07 | 2.93E-06 | AC245041.2 | lincRNA |
| ENSG00000258743.4 | 3.049835 | -2.45922 | 0.033369 | 0.071268 | LINC02301 | lincRNA |
| ENSG00000260186.4 | 3.033075 | -0.36461 | 9.45E-05 | 0.000492 | LINC02137 | lincRNA |
| ENSG00000231683.5 | 3.025112 | 4.701103 | 0.029801 | 0.064935 | AL033397.1 | lincRNA |
| ENSG00000233081.1 | 3.022963 | -2.15337 | 1.17E-07 | 1.29E-06 | AL353764.1 | lincRNA |
| ENSG00000259692.4 | 3.018133 | 0.668508 | 7.24E-11 | 1.40E-09 | AC104041.1 | lincRNA |
| ENSG00000250421.4 | 2.997442 | -0.52791 | 4.16E-07 | 4.06E-06 | AC106798.1 | lincRNA |
| ENSG00000249803.4 | 2.995048 | -2.86529 | 0.020455 | 0.047455 | AC112178.1 | lincRNA |
| ENSG00000225535.5 | 2.986758 | -1.51172 | 4.12E-07 | 4.03E-06 | LINC01393 | lincRNA |
| ENSG00000251185.1 | 2.973977 | -2.20369 | 0.000205 | 0.000967 | AC025244.1 | lincRNA |
| ENSG00000236028.1 | 2.971002 | -0.31641 | 0.035234 | 0.07444 | AL354766.2 | lincRNA |
| ENSG00000263551.4 | 2.968971 | -1.65862 | 0.000219 | 0.001024 | AP005328.1 | lincRNA |
| ENSG00000229964.1 | 2.96281 | -2.77712 | 0.006077 | 0.01735 | AC080129.1 | lincRNA |
| ENSG00000229876.1 | 2.96109 | -0.41745 | 0.00352 | 0.010954 | CASC20 | lincRNA |
| ENSG00000248515.1 | 2.96086 | -2.30092 | 0.004525 | 0.013574 | AC024230.1 | lincRNA |
| ENSG00000224271.4 | 2.949634 | 0.541044 | 1.76E-07 | 1.86E-06 | AL117329.1 | lincRNA |
| ENSG00000262681.2 | 2.949069 | 0.621214 | 0.00016 | 0.000781 | AC005722.2 | lincRNA |
| ENSG00000225096.1 | 2.948256 | -2.06193 | 0.000532 | 0.0022 | AL445250.1 | lincRNA |
| ENSG00000255345.1 | 2.934413 | -2.52854 | 0.000103 | 0.000532 | AP002957.1 | lincRNA |
| ENSG00000276476.2 | 2.928388 | 0.203107 | 0.003519 | 0.010951 | LINC00540 | lincRNA |
| ENSG00000215808.2 | 2.916556 | 1.82596 | 0.002189 | 0.007314 | LINC01139 | lincRNA |
| ENSG00000248103.1 | 2.912128 | -1.52606 | 0.001766 | 0.006089 | AC008443.2 | lincRNA |
| ENSG00000251129.1 | 2.911213 | -2.00656 | 0.023608 | 0.053481 | LINC02506 | lincRNA |
| ENSG00000249102.1 | 2.910558 | -1.81219 | 0.011041 | 0.028502 | AC034223.1 | lincRNA |
| ENSG00000229896.2 | 2.903947 | -0.83876 | 0.005228 | 0.015315 | AL157373.2 | lincRNA |
| ENSG00000224939.1 | 2.897814 | -1.00658 | 2.06E-05 | 0.00013 | LINC00184 | lincRNA |
| ENSG00000260810.1 | 2.884454 | -0.47931 | 7.74E-06 | 5.51E-05 | AL135818.2 | lincRNA |
| ENSG00000224652.1 | 2.875818 | 2.126126 | 4.33E-05 | 0.000251 | LINC00885 | lincRNA |
| ENSG00000268287.1 | 2.868266 | -1.37616 | 7.67E-10 | 1.25E-08 | AC008687.3 | lincRNA |
| ENSG00000226476.2 | 2.856058 | 1.706407 | 2.21E-07 | 2.30E-06 | LINC01748 | lincRNA |
| ENSG00000238837.3 | 2.854088 | -0.52035 | 0.001747 | 0.006035 | LINC02031 | lincRNA |
| ENSG00000258240.4 | 2.853188 | -3.31597 | 0.014362 | 0.035526 | AC002351.1 | lincRNA |
| ENSG00000176659.7 | 2.851254 | 0.721922 | 0.002692 | 0.008716 | C20orf197 | lincRNA |
| ENSG00000273415.1 | 2.826706 | -2.64224 | 0.041557 | 0.085138 | AP000904.1 | lincRNA |
| ENSG00000249731.1 | 2.825762 | -1.84477 | 0.000185 | 0.000886 | AC126768.3 | lincRNA |
| ENSG00000228541.1 | 2.79852 | -1.01092 | 0.002786 | 0.008971 | AC093159.1 | lincRNA |
| ENSG00000258479.4 | 2.792528 | 0.595756 | 2.66E-07 | 2.72E-06 | LINC00640 | lincRNA |
| ENSG00000259672.1 | 2.761692 | 0.143752 | 0.01804 | 0.042794 | AC087612.1 | lincRNA |
| ENSG00000253554.4 | 2.739671 | -2.04203 | 0.013022 | 0.032697 | AC022639.1 | lincRNA |
| ENSG00000204362.5 | 2.739197 | -1.42383 | 0.001463 | 0.00519 | AL590644.1 | lincRNA |
| ENSG00000237548.1 | 2.738367 | -1.97219 | 0.000761 | 0.00298 | TTLL11-IT1 | lincRNA |
| ENSG00000276851.1 | 2.727123 | -1.25713 | 0.002633 | 0.00855 | AC002401.4 | lincRNA |
| ENSG00000233928.4 | 2.717945 | -2.24099 | 0.00994 | 0.026147 | AL591501.1 | lincRNA |
| ENSG00000228459.3 | 2.716921 | -2.84453 | 0.000534 | 0.002207 | LINC01546 | lincRNA |
| ENSG00000275179.1 | 2.700499 | -2.5305 | 0.000838 | 0.003221 | AL118508.2 | lincRNA |
| ENSG00000132204.12 | 2.694264 | 0.536371 | 0.00738 | 0.020395 | LINC00470 | lincRNA |
| ENSG00000267325.1 | 2.690272 | 0.209445 | 6.54E-09 | 9.08E-08 | LINC01415 | lincRNA |
| ENSG00000253315.1 | 2.683012 | -0.73139 | 2.83E-05 | 0.000173 | LINC01932 | lincRNA |
| ENSG00000236039.1 | 2.678555 | 0.020828 | 0.008059 | 0.021933 | AC019117.1 | lincRNA |
| ENSG00000226674.7 | 2.677515 | 1.358851 | 0.000674 | 0.002691 | TEX41 | lincRNA |
| ENSG00000267922.1 | 2.667204 | -0.35853 | 0.001094 | 0.004034 | AC007785.1 | lincRNA |
| ENSG00000248810.1 | 2.661474 | 0.419515 | 0.042453 | 0.086683 | LINC02432 | lincRNA |
| ENSG00000228561.2 | 2.654443 | -1.05395 | 0.000473 | 0.001981 | AC026355.1 | lincRNA |
| ENSG00000258910.2 | 2.640862 | -1.78546 | 0.004423 | 0.013325 | LINC01956 | lincRNA |
| ENSG00000224899.1 | 2.630072 | -1.38653 | 0.010118 | 0.026525 | AC019155.3 | lincRNA |
| ENSG00000234210.1 | 2.623964 | -2.67218 | 0.010179 | 0.026652 | AC006372.3 | lincRNA |
| ENSG00000228971.2 | 2.606509 | -2.30393 | 0.00135 | 0.004837 | AL356479.1 | lincRNA |
| ENSG00000250697.1 | 2.600551 | -0.00727 | 0.015726 | 0.038215 | AC010343.3 | lincRNA |
| ENSG00000235770.4 | 2.577965 | 0.123678 | 0.015109 | 0.037003 | LINC00607 | lincRNA |
| ENSG00000205334.2 | 2.577878 | -1.45489 | 0.000555 | 0.002283 | LINC01460 | lincRNA |
| ENSG00000177234.7 | 2.577016 | -1.75052 | 0.000182 | 0.000872 | LINC01561 | lincRNA |
| ENSG00000259786.5 | 2.568588 | -1.69383 | 0.009115 | 0.024309 | LINC02109 | lincRNA |
| ENSG00000231453.1 | 2.560313 | -0.7968 | 0.000507 | 0.002109 | LINC01305 | lincRNA |
| ENSG00000248994.1 | 2.549978 | 0.615233 | 1.34E-06 | 1.16E-05 | AC126768.2 | lincRNA |
| ENSG00000249413.2 | 2.546169 | -1.37047 | 0.000305 | 0.001361 | AC116049.2 | lincRNA |
| ENSG00000258942.1 | 2.536578 | -1.74472 | 7.10E-06 | 5.11E-05 | AL358332.1 | lincRNA |
| ENSG00000235493.1 | 2.528837 | -0.18147 | 0.000327 | 0.001443 | LINC01967 | lincRNA |
| ENSG00000232053.5 | 2.528632 | -1.75609 | 0.002115 | 0.007099 | AC078845.1 | lincRNA |
| ENSG00000253227.1 | 2.528333 | -2.46954 | 0.003858 | 0.011848 | AC090192.2 | lincRNA |
| ENSG00000271830.1 | 2.521159 | -1.71274 | 0.000871 | 0.003325 | AC012213.4 | lincRNA |
| ENSG00000253669.3 | 2.51711 | 1.152886 | 5.62E-07 | 5.35E-06 | GASAL1 | lincRNA |
| ENSG00000257219.4 | 2.513544 | -2.18195 | 4.14E-05 | 0.000241 | LINC02407 | lincRNA |
| ENSG00000244578.1 | 2.484673 | -1.22187 | 0.007043 | 0.019637 | LINC01391 | lincRNA |
| ENSG00000228933.6 | 2.484626 | -2.30441 | 0.013982 | 0.034686 | AC107419.1 | lincRNA |
| ENSG00000229912.1 | 2.482841 | -1.37331 | 0.001718 | 0.005949 | AC128709.1 | lincRNA |
| ENSG00000233901.4 | 2.478372 | 3.22828 | 1.24E-09 | 1.98E-08 | LINC01503 | lincRNA |
| ENSG00000243491.1 | 2.46917 | 0.420535 | 3.92E-05 | 0.000229 | AC082651.3 | lincRNA |
| ENSG00000244128.4 | 2.462088 | 0.849113 | 0.006892 | 0.019287 | LINC01322 | lincRNA |
| ENSG00000237594.2 | 2.461791 | -0.53673 | 2.15E-06 | 1.77E-05 | AP000251.1 | lincRNA |
| ENSG00000280650.1 | 2.456843 | -3.02505 | 0.011683 | 0.029851 | KCNIP4-IT1 | lincRNA |
| ENSG00000246876.5 | 2.456744 | -1.16854 | 0.008038 | 0.021888 | LINC02466 | lincRNA |
| ENSG00000232164.1 | 2.446786 | -1.93082 | 1.09E-05 | 7.45E-05 | LINC01873 | lincRNA |
| ENSG00000227676.3 | 2.443841 | -1.86236 | 0.004471 | 0.013442 | LINC01068 | lincRNA |
| ENSG00000280890.1 | 2.43299 | -2.27548 | 0.000957 | 0.003611 | ELDR | lincRNA |
| ENSG00000280721.1 | 2.419754 | -0.69395 | 6.32E-11 | 1.24E-09 | LINC01943 | lincRNA |
| ENSG00000249842.1 | 2.408718 | -2.42857 | 0.041405 | 0.084905 | AC010486.3 | lincRNA |
| ENSG00000279141.2 | 2.399836 | 1.318043 | 0.000343 | 0.00151 | LINC01451 | lincRNA |
| ENSG00000163364.8 | 2.396121 | 1.62339 | 2.35E-05 | 0.000147 | LINC01116 | lincRNA |
| ENSG00000257869.1 | 2.389725 | -2.04446 | 0.037591 | 0.078544 | AL139023.1 | lincRNA |
| ENSG00000244791.2 | 2.388019 | -2.78967 | 0.008111 | 0.022054 | AC087667.1 | lincRNA |
| ENSG00000246095.2 | 2.38418 | -1.39277 | 0.001033 | 0.003844 | LINC01096 | lincRNA |
| ENSG00000266088.4 | 2.377519 | -0.94239 | 4.53E-05 | 0.00026 | AC004585.1 | lincRNA |
| ENSG00000248597.1 | 2.371149 | -1.54032 | 0.000197 | 0.000933 | AC126768.1 | lincRNA |
| ENSG00000277587.1 | 2.365867 | -0.27601 | 0.025406 | 0.056867 | AC008759.3 | lincRNA |
| ENSG00000251381.5 | 2.364902 | 4.719872 | 0.000198 | 0.000939 | LINC00958 | lincRNA |
| ENSG00000234948.1 | 2.34649 | -2.5384 | 0.001347 | 0.00483 | LINC01524 | lincRNA |
| ENSG00000249395.2 | 2.334503 | 3.36721 | 0.000298 | 0.001332 | CASC9 | lincRNA |
| ENSG00000250041.2 | 2.333421 | -1.26947 | 0.00015 | 0.000739 | AC069360.1 | lincRNA |
| ENSG00000172965.13 | 2.321336 | 4.241231 | 9.03E-15 | 2.87E-13 | MIR4435-2HG | lincRNA |
| ENSG00000273341.1 | 2.32077 | -1.4036 | 0.01007 | 0.026429 | AC004921.1 | lincRNA |
| ENSG00000230943.1 | 2.319383 | 1.307636 | 5.22E-08 | 6.17E-07 | LINC02541 | lincRNA |
| ENSG00000266869.1 | 2.308091 | -0.96471 | 0.000688 | 0.002737 | AC005993.1 | lincRNA |
| ENSG00000258053.1 | 2.304435 | 0.642216 | 0.002845 | 0.009139 | AC025575.2 | lincRNA |
| ENSG00000256417.1 | 2.303683 | 1.223134 | 0.00014 | 0.000698 | AC006206.2 | lincRNA |
| ENSG00000253507.4 | 2.301863 | -3.22956 | 0.027355 | 0.060531 | AC104257.1 | lincRNA |
| ENSG00000261848.4 | 2.293448 | -2.43018 | 0.003376 | 0.010557 | AC090282.1 | lincRNA |
| ENSG00000163009.7 | 2.283699 | 0.395043 | 3.88E-06 | 2.99E-05 | C2orf48 | lincRNA |
| ENSG00000205325.1 | 2.259715 | -1.54299 | 0.022197 | 0.05078 | AC005863.1 | lincRNA |
| ENSG00000247317.3 | 2.255175 | 0.713966 | 3.42E-07 | 3.41E-06 | LY6E-DT | lincRNA |
| ENSG00000225383.5 | 2.230836 | -2.383 | 0.003747 | 0.011575 | SFTA1P | lincRNA |
| ENSG00000258279.2 | 2.2278 | -1.16036 | 9.03E-05 | 0.000472 | LINC00592 | lincRNA |
| ENSG00000261807.2 | 2.225821 | -1.6014 | 0.02964 | 0.064645 | LINC02141 | lincRNA |
| ENSG00000222041.9 | 2.225578 | 3.276609 | 2.80E-12 | 6.66E-11 | CYTOR | lincRNA |
| ENSG00000253746.1 | 2.225219 | 0.729614 | 0.001081 | 0.003995 | AC091182.2 | lincRNA |
| ENSG00000236347.1 | 2.215691 | -0.79517 | 0.000375 | 0.001626 | AL513123.1 | lincRNA |
| ENSG00000274840.3 | 2.214415 | -1.83049 | 0.045697 | 0.091943 | AC132807.2 | lincRNA |
| ENSG00000255983.1 | 2.211464 | -0.87512 | 0.000831 | 0.003197 | AC007848.1 | lincRNA |
| ENSG00000267374.1 | 2.207333 | 0.891312 | 5.02E-05 | 0.000284 | AC016205.1 | lincRNA |
| ENSG00000234362.4 | 2.185776 | -2.19971 | 0.000616 | 0.002489 | LINC01914 | lincRNA |
| ENSG00000249859.6 | 2.182962 | 4.318563 | 4.83E-11 | 9.65E-10 | PVT1 | lincRNA |
| ENSG00000236924.1 | 2.17948 | -2.27374 | 0.00644 | 0.018223 | AL162411.1 | lincRNA |
| ENSG00000227403.1 | 2.178273 | 2.5656 | 1.07E-05 | 7.32E-05 | LINC01806 | lincRNA |
| ENSG00000267665.1 | 2.17613 | -1.86623 | 0.01067 | 0.027708 | AC021683.3 | lincRNA |
| ENSG00000228044.2 | 2.170618 | -1.05895 | 0.000308 | 0.00137 | AL160408.1 | lincRNA |
| ENSG00000228697.1 | 2.170001 | -1.2141 | 7.31E-05 | 0.000395 | AL023755.1 | lincRNA |
| ENSG00000231346.4 | 2.164715 | 0.836465 | 0.004311 | 0.013028 | LINC01160 | lincRNA |
| ENSG00000185904.10 | 2.1637 | 2.046198 | 0.000757 | 0.002966 | LINC00839 | lincRNA |
| ENSG00000278266.1 | 2.155154 | 1.475171 | 0.00102 | 0.003803 | AC079949.2 | lincRNA |
| ENSG00000249816.5 | 2.1509 | 0.822713 | 0.001826 | 0.006263 | LINC00964 | lincRNA |
| ENSG00000232233.1 | 2.139736 | -1.17937 | 4.75E-05 | 0.000271 | LINC02043 | lincRNA |
| ENSG00000229422.1 | 2.138592 | -1.02468 | 8.83E-05 | 0.000464 | AL512625.2 | lincRNA |
| ENSG00000249022.1 | 2.13565 | -2.45622 | 0.013683 | 0.034063 | AC093599.1 | lincRNA |
| ENSG00000278238.1 | 2.131683 | -0.43618 | 1.14E-06 | 1.00E-05 | AL359513.1 | lincRNA |
| ENSG00000229278.1 | 2.12467 | -1.05995 | 1.27E-05 | 8.49E-05 | AL133353.1 | bidirectional_promoter_lncRNA |
| ENSG00000235884.3 | 2.123631 | 1.236583 | 0.003362 | 0.010519 | LINC00941 | lincRNA |
| ENSG00000272328.1 | 2.120187 | -1.47489 | 0.002564 | 0.008365 | AC007128.2 | lincRNA |
| ENSG00000224577.1 | 2.104571 | -1.12141 | 0.000144 | 0.000713 | LINC01117 | lincRNA |
| ENSG00000176912.3 | 2.095135 | 0.873017 | 2.42E-07 | 2.49E-06 | TYMSOS | bidirectional_promoter_lncRNA |
| ENSG00000187013.4 | 2.084891 | -0.75161 | 4.70E-05 | 0.000269 | C17orf82 | lincRNA |
| ENSG00000236740.5 | 2.084423 | 2.480287 | 0.000401 | 0.001721 | AL033384.1 | lincRNA |
| ENSG00000272512.1 | 2.070866 | 1.602316 | 7.33E-05 | 0.000396 | AL645608.7 | lincRNA |
| ENSG00000248599.1 | 2.058735 | -1.11906 | 0.004838 | 0.014363 | FLJ42969 | lincRNA |
| ENSG00000232679.1 | 2.057254 | -0.74271 | 0.008917 | 0.023842 | LINC01705 | lincRNA |
| ENSG00000231298.5 | 2.051698 | 0.224989 | 0.002155 | 0.007216 | MANCR | lincRNA |
| ENSG00000267372.2 | 2.047994 | -1.30021 | 0.001501 | 0.005307 | AC005330.1 | lincRNA |
| ENSG00000259225.5 | 2.045955 | -0.40881 | 0.003524 | 0.010964 | LINC02345 | lincRNA |
| ENSG00000232324.1 | 2.039866 | -1.31828 | 0.025064 | 0.056239 | AC008440.3 | lincRNA |
| ENSG00000227012.2 | 2.031356 | -2.87596 | 0.032908 | 0.070418 | LINC02527 | lincRNA |
| ENSG00000232677.5 | 2.029855 | 4.339754 | 0.001894 | 0.006468 | LINC00665 | lincRNA |
| ENSG00000246465.1 | 2.015514 | -1.12019 | 0.006018 | 0.017206 | AC138904.1 | lincRNA |
| ENSG00000238113.5 | 2.015365 | 1.752415 | 2.17E-07 | 2.26E-06 | LINC01410 | lincRNA |
| ENSG00000253161.4 | 2.01456 | 2.459937 | 0.001279 | 0.004617 | LINC01605 | lincRNA |
| ENSG00000234546.2 | 2.01369 | 0.480476 | 5.81E-10 | 9.72E-09 | LNCTAM34A | lincRNA |
| ENSG00000278389.1 | 2.011496 | -0.85071 | 0.00085 | 0.003259 | AC099518.6 | lincRNA |
| ENSG00000248360.6 | 2.005458 | 2.059677 | 0.004293 | 0.012984 | LINC00504 | lincRNA |
| ENSG00000271888.1 | 2.003909 | -0.55339 | 8.86E-07 | 8.05E-06 | AL136162.1 | lincRNA |
| ENSG00000272168.4 | 2.001889 | 2.566296 | 1.04E-05 | 7.18E-05 | CASC15 | lincRNA |
| ENSG00000249378.4 | 1.98913 | -1.67371 | 0.001864 | 0.006378 | LINC01060 | lincRNA |
| ENSG00000258592.1 | 1.985689 | -1.75972 | 2.55E-05 | 0.000158 | AL391152.1 | lincRNA |
| ENSG00000212766.8 | 1.979035 | -1.25711 | 0.012883 | 0.032423 | EWSAT1 | lincRNA |
| ENSG00000235979.7 | 1.978011 | -1.35486 | 0.046825 | 0.09379 | AC004448.2 | lincRNA |
| ENSG00000234076.1 | 1.97326 | -0.50626 | 0.000397 | 0.001708 | TPRG1-AS1 | lincRNA |
| ENSG00000259230.1 | 1.968958 | 1.676383 | 0.005136 | 0.015092 | LINC02323 | lincRNA |
| ENSG00000260430.1 | 1.96731 | -1.5809 | 0.004808 | 0.014286 | AC099518.1 | lincRNA |
| ENSG00000267279.1 | 1.949933 | -1.28891 | 0.00196 | 0.006663 | AC090409.1 | lincRNA |
| ENSG00000227533.4 | 1.948424 | 1.439006 | 2.04E-05 | 0.000129 | SLC2A1-AS1 | lincRNA |
| ENSG00000279712.1 | 1.93506 | -0.27653 | 0.001341 | 0.004809 | FP325330.3 | lincRNA |
| ENSG00000237787.4 | 1.927465 | -2.12218 | 0.027455 | 0.060715 | C3orf79 | lincRNA |
| ENSG00000259828.1 | 1.91856 | -1.52773 | 0.008222 | 0.022305 | AL355596.1 | lincRNA |
| ENSG00000256128.4 | 1.914409 | -0.27279 | 0.003671 | 0.011371 | LINC00944 | lincRNA |
| ENSG00000270190.1 | 1.903764 | -0.5621 | 1.67E-06 | 1.41E-05 | AC068491.3 | lincRNA |
| ENSG00000180066.8 | 1.886806 | 1.142943 | 0.00098 | 0.003684 | C10orf91 | lincRNA |
| ENSG00000267452.1 | 1.882596 | -2.444 | 0.014875 | 0.036554 | LINC02073 | lincRNA |
| ENSG00000258733.4 | 1.879456 | -0.71784 | 0.000219 | 0.001024 | LINC02328 | lincRNA |
| ENSG00000254842.5 | 1.863068 | -1.09471 | 0.006196 | 0.017645 | LINC02551 | lincRNA |
| ENSG00000267226.2 | 1.859811 | -1.7908 | 3.42E-05 | 0.000204 | AC104971.1 | bidirectional_promoter_lncRNA |
| ENSG00000233858.4 | 1.858704 | -2.83705 | 0.007255 | 0.020115 | LINC02599 | lincRNA |
| ENSG00000280206.1 | 1.848923 | 3.47875 | 3.16E-10 | 5.56E-09 | AC026401.3 | lincRNA |
| ENSG00000253417.4 | 1.841542 | 0.111996 | 0.003321 | 0.010426 | LINC02159 | lincRNA |
| ENSG00000224260.5 | 1.838479 | -0.56593 | 0.006607 | 0.018622 | AL023754.1 | lincRNA |
| ENSG00000253395.1 | 1.835259 | -1.56072 | 1.54E-05 | 0.000101 | AP003469.2 | lincRNA |
| ENSG00000272347.1 | 1.833805 | -2.02105 | 0.001044 | 0.003877 | AC116351.2 | lincRNA |
| ENSG00000236651.1 | 1.832108 | -2.20671 | 0.01178 | 0.030064 | DLX2-DT | lincRNA |
| ENSG00000232774.6 | 1.827567 | 0.139564 | 0.000118 | 0.000597 | FLJ22447 | lincRNA |
| ENSG00000259380.4 | 1.820934 | -2.97645 | 0.015588 | 0.037947 | AC087473.1 | lincRNA |
| ENSG00000205562.2 | 1.820139 | -0.81973 | 0.011335 | 0.029105 | AL049775.1 | lincRNA |
| ENSG00000245522.2 | 1.813785 | -0.1175 | 9.16E-06 | 6.41E-05 | AC026250.1 | lincRNA |
| ENSG00000260086.2 | 1.804851 | -0.84668 | 8.61E-05 | 0.000454 | AC007611.1 | lincRNA |
| ENSG00000256955.2 | 1.802139 | -2.31639 | 0.022912 | 0.052181 | AC131009.2 | lincRNA |
| ENSG00000231528.2 | 1.79568 | -1.6476 | 0.000164 | 0.000796 | FAM225A | lincRNA |
| ENSG00000175728.3 | 1.792035 | -2.66046 | 0.02207 | 0.050535 | C11orf44 | lincRNA |
| ENSG00000259807.1 | 1.789983 | -0.29192 | 0.000622 | 0.002512 | AC009093.1 | lincRNA |
| ENSG00000189229.10 | 1.780305 | -0.3709 | 0.00257 | 0.008381 | AC069277.1 | lincRNA |
| ENSG00000232006.7 | 1.779178 | 0.874691 | 7.34E-06 | 5.25E-05 | AC005537.1 | lincRNA |
| ENSG00000235947.1 | 1.769006 | -1.67347 | 0.006754 | 0.018974 | EGOT | lincRNA |
| ENSG00000224843.5 | 1.764881 | 0.783797 | 0.000372 | 0.001618 | LINC00240 | lincRNA |
| ENSG00000277013.1 | 1.762402 | 3.064849 | 0.000162 | 0.000791 | AC008556.1 | lincRNA |
| ENSG00000277287.1 | 1.749535 | 2.529695 | 0.000952 | 0.003599 | AL109976.1 | lincRNA |
| ENSG00000244541.4 | 1.734289 | -2.64906 | 0.032357 | 0.069477 | LINC01213 | lincRNA |
| ENSG00000217258.2 | 1.724109 | -1.49595 | 0.022081 | 0.050556 | AC007249.1 | lincRNA |
| ENSG00000232386.8 | 1.723057 | -0.65606 | 0.001149 | 0.004208 | AC015712.1 | lincRNA |
| ENSG00000258791.6 | 1.714784 | 1.493351 | 0.033717 | 0.071854 | LINC00520 | lincRNA |
| ENSG00000231772.4 | 1.71396 | -1.65612 | 0.004272 | 0.012929 | Z93403.1 | lincRNA |
| ENSG00000256001.1 | 1.700881 | -0.49517 | 0.00517 | 0.015175 | AC079949.1 | lincRNA |
| ENSG00000273760.1 | 1.68788 | 2.72592 | 0.020409 | 0.047365 | AC245041.1 | lincRNA |
| ENSG00000247134.5 | 1.682212 | 0.411599 | 0.020771 | 0.048028 | AC090204.1 | lincRNA |
| ENSG00000250748.5 | 1.65605 | -1.74632 | 0.021757 | 0.049946 | AC025419.1 | lincRNA |
| ENSG00000224397.4 | 1.649338 | 1.345037 | 9.42E-06 | 6.58E-05 | SMIM25 | lincRNA |
| ENSG00000256146.1 | 1.646972 | -0.47784 | 0.008693 | 0.023359 | AC006206.1 | lincRNA |
| ENSG00000281162.1 | 1.646551 | 0.731549 | 0.019923 | 0.046432 | LINC01127 | lincRNA |
| ENSG00000233791.4 | 1.62885 | -1.90298 | 0.001397 | 0.004981 | LINC01136 | lincRNA |
| ENSG00000272711.1 | 1.625457 | 0.951262 | 9.80E-05 | 0.000508 | AC019069.1 | lincRNA |
| ENSG00000236481.1 | 1.623465 | -2.14387 | 0.004587 | 0.013733 | LINC02195 | lincRNA |
| ENSG00000280145.2 | 1.612297 | -2.28416 | 0.034155 | 0.072612 | CU638689.4 | lincRNA |
| ENSG00000225077.2 | 1.595754 | -1.68189 | 0.000594 | 0.002415 | LINC00337 | lincRNA |
| ENSG00000259594.4 | 1.588085 | -1.49477 | 0.000675 | 0.002693 | AC023034.1 | lincRNA |
| ENSG00000260423.1 | 1.586186 | 0.038585 | 0.000422 | 0.001798 | LINC02367 | lincRNA |
| ENSG00000231566.1 | 1.586165 | -0.29255 | 0.002582 | 0.008412 | LINC02595 | lincRNA |
| ENSG00000235725.1 | 1.56223 | -2.20071 | 0.011841 | 0.030209 | AC007389.3 | lincRNA |
| ENSG00000206532.2 | 1.558917 | -0.42879 | 0.004101 | 0.012483 | AC117402.1 | lincRNA |
| ENSG00000261504.1 | 1.54659 | -1.61637 | 0.00274 | 0.008849 | LINC01686 | lincRNA |
| ENSG00000240893.1 | 1.543221 | -0.78433 | 0.030296 | 0.065778 | LINC02042 | lincRNA |
| ENSG00000227373.4 | 1.541629 | -1.83546 | 0.000785 | 0.003049 | AL121983.2 | lincRNA |
| ENSG00000237923.1 | 1.538787 | -2.15149 | 0.048022 | 0.095684 | LINC02570 | lincRNA |
| ENSG00000254615.2 | 1.529094 | 0.531253 | 0.000388 | 0.001675 | AC027031.2 | lincRNA |
| ENSG00000259869.1 | 1.525796 | -1.48626 | 0.00269 | 0.008708 | AL022344.2 | lincRNA |
| ENSG00000234902.5 | 1.518992 | -1.00162 | 0.016515 | 0.039801 | AC007879.3 | lincRNA |
| ENSG00000256443.1 | 1.512923 | -1.27525 | 0.005289 | 0.015446 | AP003559.1 | lincRNA |
| ENSG00000255362.1 | 1.501223 | -2.38551 | 0.029435 | 0.064301 | AP000785.2 | lincRNA |
| ENSG00000231483.1 | 1.494639 | -1.10616 | 0.010823 | 0.028025 | AL365356.4 | lincRNA |
| ENSG00000278514.1 | 1.490821 | -1.55398 | 0.00041 | 0.001753 | AC068831.6 | lincRNA |
| ENSG00000259345.4 | 1.486573 | 0.961166 | 7.64E-05 | 0.000411 | AC013652.1 | lincRNA |
| ENSG00000203999.7 | 1.479398 | -0.29071 | 0.000401 | 0.00172 | LINC01270 | lincRNA |
| ENSG00000261222.2 | 1.473379 | -2.29355 | 0.044423 | 0.089845 | AC064805.1 | lincRNA |
| ENSG00000266970.1 | 1.462818 | -1.18445 | 0.000699 | 0.002769 | AC061992.1 | lincRNA |
| ENSG00000233723.6 | 1.458528 | -2.56889 | 0.047256 | 0.094542 | LINC01122 | lincRNA |
| ENSG00000272663.1 | 1.45643 | -2.31868 | 0.000891 | 0.003393 | AC093635.1 | lincRNA |
| ENSG00000251026.1 | 1.453503 | -1.01338 | 0.016707 | 0.040161 | LINC02163 | lincRNA |
| ENSG00000272425.1 | 1.452333 | -1.21648 | 0.000416 | 0.001776 | AC009902.3 | lincRNA |
| ENSG00000182366.8 | 1.452212 | -1.23205 | 0.002914 | 0.00933 | FAM87A | lincRNA |
| ENSG00000277511.1 | 1.449998 | -0.23135 | 4.51E-05 | 0.000259 | AC116407.2 | lincRNA |
| ENSG00000248693.1 | 1.449259 | -1.77167 | 0.012168 | 0.030909 | LINC02100 | lincRNA |
| ENSG00000260597.1 | 1.447725 | -0.47991 | 0.01124 | 0.028916 | AC012531.1 | lincRNA |
| ENSG00000260377.1 | 1.439288 | -1.33833 | 0.019891 | 0.046374 | AC083801.2 | lincRNA |
| ENSG00000268601.1 | 1.4382 | 0.138554 | 0.01681 | 0.040368 | AC115522.1 | lincRNA |
| ENSG00000269974.1 | 1.436834 | -1.06893 | 2.25E-05 | 0.000141 | AC091057.4 | lincRNA |
| ENSG00000214145.5 | 1.433971 | 0.915791 | 0.017699 | 0.042111 | LINC00887 | lincRNA |
| ENSG00000261996.1 | 1.433872 | -0.48051 | 0.005988 | 0.017138 | AC004706.1 | lincRNA |
| ENSG00000231327.1 | 1.426235 | 0.049733 | 0.002476 | 0.008124 | LINC01816 | lincRNA |
| ENSG00000261135.1 | 1.411148 | -1.90782 | 0.004672 | 0.013952 | AL137802.2 | lincRNA |
| ENSG00000248161.4 | 1.399662 | -0.86485 | 0.000424 | 0.001804 | AC098487.1 | lincRNA |
| ENSG00000271855.1 | 1.398524 | -0.84062 | 9.66E-05 | 0.000501 | AC073195.1 | lincRNA |
| ENSG00000271811.1 | 1.393419 | -0.10275 | 0.006646 | 0.018706 | Z97200.1 | lincRNA |
| ENSG00000273366.1 | 1.385706 | 0.196231 | 8.98E-06 | 6.30E-05 | Z83851.2 | lincRNA |
| ENSG00000248243.1 | 1.38126 | -0.9945 | 0.031015 | 0.067094 | LINC02014 | lincRNA |
| ENSG00000225506.2 | 1.375947 | -1.29792 | 0.002701 | 0.008739 | CYP4A22-AS1 | lincRNA |
| ENSG00000270977.1 | 1.374809 | -0.52778 | 0.01655 | 0.039872 | AC015849.5 | lincRNA |
| ENSG00000249740.1 | 1.371088 | -0.20536 | 9.59E-05 | 0.000498 | OSMR-AS1 | lincRNA |
| ENSG00000268129.1 | 1.370807 | -1.85145 | 0.002275 | 0.007564 | AC026304.1 | lincRNA |
| ENSG00000273117.1 | 1.368231 | 0.642265 | 0.000995 | 0.00373 | AC144652.1 | lincRNA |
| ENSG00000272468.1 | 1.366964 | -0.07367 | 0.02312 | 0.052542 | AL021807.1 | lincRNA |
| ENSG00000224961.1 | 1.357483 | 0.285241 | 0.017299 | 0.041327 | LINC01752 | lincRNA |
| ENSG00000272829.1 | 1.356712 | -1.09077 | 0.000966 | 0.003641 | AC002470.1 | lincRNA |
| ENSG00000189419.6 | 1.356462 | -0.37477 | 0.0001 | 0.000518 | SPATA41 | lincRNA |
| ENSG00000278071.1 | 1.353486 | -1.08116 | 0.026548 | 0.059025 | AL161669.3 | lincRNA |
| ENSG00000255571.5 | 1.351267 | 1.959087 | 0.034793 | 0.073673 | MIR9-3HG | lincRNA |
| ENSG00000256433.2 | 1.348235 | 1.117126 | 0.00031 | 0.00138 | AC005840.2 | lincRNA |
| ENSG00000225518.2 | 1.343708 | 0.266703 | 0.000767 | 0.002996 | LINC01703 | lincRNA |
| ENSG00000259178.1 | 1.341649 | -0.80403 | 0.024424 | 0.055005 | AC023906.2 | lincRNA |
| ENSG00000261997.1 | 1.325327 | 0.069314 | 0.000328 | 0.001448 | AC007336.1 | lincRNA |
| ENSG00000220161.4 | 1.322964 | -1.64827 | 0.005316 | 0.015516 | LINC02076 | lincRNA |
| ENSG00000236908.2 | 1.319581 | -0.69231 | 0.046247 | 0.092806 | AC005865.1 | lincRNA |
| ENSG00000256540.1 | 1.296381 | -1.85734 | 0.001663 | 0.005794 | AC007406.3 | lincRNA |
| ENSG00000260805.2 | 1.285668 | 0.395434 | 0.001756 | 0.00606 | AC092803.2 | lincRNA |
| ENSG00000175170.13 | 1.282243 | 0.124722 | 0.008694 | 0.023361 | FAM182B | lincRNA |
| ENSG00000249364.4 | 1.282008 | -1.2237 | 0.01307 | 0.03279 | AC112206.2 | lincRNA |
| ENSG00000278916.1 | 1.280431 | -1.23517 | 0.001033 | 0.003845 | CEP83-DT | lincRNA |
| ENSG00000235888.1 | 1.279902 | -0.86392 | 0.017355 | 0.041433 | AF064858.1 | lincRNA |
| ENSG00000260193.1 | 1.27642 | -1.03112 | 0.003212 | 0.010128 | AL138781.1 | lincRNA |
| ENSG00000225783.5 | 1.266757 | 2.850392 | 0.010399 | 0.027145 | MIAT | lincRNA |
| ENSG00000246640.1 | 1.24835 | -0.51845 | 0.002646 | 0.008589 | PICART1 | lincRNA |
| ENSG00000242516.1 | 1.247727 | 1.558191 | 0.019851 | 0.046308 | LINC00960 | lincRNA |
| ENSG00000260588.1 | 1.246256 | -0.02881 | 0.003444 | 0.010741 | AC027702.1 | lincRNA |
| ENSG00000267321.1 | 1.2436 | 2.52197 | 9.81E-06 | 6.82E-05 | LINC02001 | lincRNA |
| ENSG00000260265.1 | 1.242455 | 1.622296 | 0.002631 | 0.008548 | LINC02562 | lincRNA |
| ENSG00000251161.3 | 1.229034 | 0.006582 | 0.000848 | 0.003253 | AC020661.1 | lincRNA |
| ENSG00000273004.1 | 1.225924 | -1.26262 | 7.93E-05 | 0.000423 | AL078644.1 | lincRNA |
| ENSG00000254973.1 | 1.221068 | 0.068903 | 0.00185 | 0.006335 | AC105219.3 | lincRNA |
| ENSG00000258537.4 | 1.213815 | -1.41694 | 0.018096 | 0.042909 | FRMD6-AS2 | lincRNA |
| ENSG00000224592.4 | 1.209648 | -1.06382 | 0.041554 | 0.085138 | AL139158.2 | lincRNA |
| ENSG00000251442.4 | 1.201143 | 1.237662 | 0.002779 | 0.008954 | LINC01094 | lincRNA |
| ENSG00000229178.1 | 1.179018 | -1.51192 | 0.00485 | 0.014396 | AC233280.1 | lincRNA |
| ENSG00000235806.1 | 1.159631 | -2.38569 | 0.013274 | 0.033198 | AF241728.1 | lincRNA |
| ENSG00000257924.1 | 1.140201 | -2.04946 | 0.024167 | 0.054527 | LINC02416 | lincRNA |
| ENSG00000272853.1 | 1.130393 | -0.6614 | 4.31E-06 | 3.27E-05 | AC069544.1 | lincRNA |
| ENSG00000272449.1 | 1.129731 | -0.21731 | 0.00976 | 0.025736 | AL139246.5 | lincRNA |
| ENSG00000226853.2 | 1.116643 | -0.66387 | 0.000326 | 0.00144 | AC010894.2 | lincRNA |
| ENSG00000267416.1 | 1.11262 | -1.01462 | 0.007814 | 0.021365 | AC025048.4 | lincRNA |
| ENSG00000243701.4 | 1.109683 | 1.980843 | 0.001271 | 0.00459 | DUBR | lincRNA |
| ENSG00000273297.1 | 1.106211 | -0.29957 | 0.04573 | 0.092 | AC009275.1 | lincRNA |
| ENSG00000258048.1 | 1.105893 | -0.2061 | 0.000144 | 0.000714 | AC073569.2 | lincRNA |
| ENSG00000233903.2 | 1.097547 | 1.217152 | 0.000202 | 0.000955 | Z83851.1 | lincRNA |
| ENSG00000259240.1 | 1.096214 | -0.67783 | 0.007887 | 0.021535 | MIR4713HG | lincRNA |
| ENSG00000226856.4 | 1.071016 | -1.63533 | 0.019678 | 0.04598 | THORLNC | lincRNA |
| ENSG00000258667.1 | 1.063872 | -1.09647 | 0.012462 | 0.031553 | HIF1A-AS2 | lincRNA |
| ENSG00000277559.1 | 1.059402 | 0.704929 | 0.002104 | 0.007068 | AC018553.1 | lincRNA |
| ENSG00000234129.6 | 1.057601 | -1.4228 | 0.000558 | 0.002292 | AC073529.1 | lincRNA |
| ENSG00000261889.1 | 1.052771 | -0.84649 | 0.00442 | 0.013316 | AC108134.2 | lincRNA |
| ENSG00000275632.1 | 1.044109 | -0.22377 | 0.00222 | 0.007402 | AL035461.2 | lincRNA |
| ENSG00000274213.1 | 1.041692 | -0.07181 | 0.004557 | 0.013654 | AC015912.3 | lincRNA |
| ENSG00000260231.1 | 1.031006 | 3.581112 | 0.009346 | 0.024808 | KDM7A-DT | bidirectional_promoter_lncRNA |
| ENSG00000260743.1 | 1.030149 | -0.49004 | 0.002894 | 0.009276 | AC007823.1 | lincRNA |
| ENSG00000281706.1 | 1.020444 | -0.15245 | 0.011085 | 0.02859 | LINC01012 | lincRNA |
| ENSG00000267152.1 | -1.00203 | 0.753658 | 0.000334 | 0.00147 | AC093227.1 | lincRNA |
| ENSG00000234690.5 | -1.01654 | -2.77166 | 0.036035 | 0.07578 | AC106869.1 | lincRNA |
| ENSG00000272754.1 | -1.02302 | -1.36822 | 0.000866 | 0.003312 | AL133245.1 | lincRNA |
| ENSG00000268087.1 | -1.02358 | -0.30872 | 4.30E-05 | 0.00025 | AC008764.2 | lincRNA |
| ENSG00000275580.1 | -1.03452 | -0.69425 | 4.14E-06 | 3.16E-05 | AC022306.2 | lincRNA |
| ENSG00000259673.4 | -1.03537 | 2.329776 | 1.58E-07 | 1.69E-06 | IQCH-AS1 | lincRNA |
| ENSG00000279927.1 | -1.0431 | -2.99937 | 0.035987 | 0.075718 | Z95114.4 | lincRNA |
| ENSG00000238005.2 | -1.04724 | -2.01399 | 0.020314 | 0.047191 | AL391832.2 | lincRNA |
| ENSG00000266256.1 | -1.04862 | -1.47918 | 0.018264 | 0.043253 | LINC00683 | lincRNA |
| ENSG00000198221.8 | -1.04932 | -0.48991 | 0.002671 | 0.008659 | AFDN-DT | lincRNA |
| ENSG00000231690.2 | -1.05119 | -2.51589 | 0.007264 | 0.02013 | LINC00574 | lincRNA |
| ENSG00000245937.6 | -1.05134 | 4.019166 | 7.49E-10 | 1.23E-08 | LINC01184 | lincRNA |
| ENSG00000271553.1 | -1.06619 | -0.54074 | 0.000179 | 0.000862 | AC018638.7 | lincRNA |
| ENSG00000281181.1 | -1.06685 | 0.487162 | 0.026062 | 0.058117 | FP236383.3 | lincRNA |
| ENSG00000259721.1 | -1.07002 | -1.25541 | 0.015096 | 0.036982 | AC090877.2 | lincRNA |
| ENSG00000240875.4 | -1.07158 | 1.419406 | 0.003489 | 0.010868 | LINC00886 | lincRNA |
| ENSG00000231367.4 | -1.08264 | -1.146 | 0.003425 | 0.01069 | AC011247.1 | lincRNA |
| ENSG00000277159.1 | -1.09204 | -1.51355 | 0.002122 | 0.007122 | AL139384.2 | lincRNA |
| ENSG00000255458.4 | -1.09824 | -1.88481 | 0.000641 | 0.002576 | AC108471.2 | lincRNA |
| ENSG00000203711.10 | -1.10872 | -1.87529 | 0.000232 | 0.001074 | C6orf99 | lincRNA |
| ENSG00000255455.2 | -1.10932 | 2.74871 | 1.06E-07 | 1.18E-06 | AP003486.1 | lincRNA |
| ENSG00000230487.6 | -1.11527 | 1.993508 | 7.66E-05 | 0.000411 | PSMG3-AS1 | lincRNA |
| ENSG00000261064.1 | -1.12354 | -1.19721 | 8.70E-05 | 0.000458 | LINC02256 | lincRNA |
| ENSG00000274173.1 | -1.13514 | 3.07744 | 0.007361 | 0.020349 | AL035661.1 | lincRNA |
| ENSG00000271327.1 | -1.13532 | -1.44122 | 0.000103 | 0.000529 | AC010201.2 | lincRNA |
| ENSG00000267940.1 | -1.14151 | -1.13265 | 4.64E-05 | 0.000266 | AC022762.2 | lincRNA |
| ENSG00000266904.4 | -1.14907 | 0.137856 | 1.15E-06 | 1.02E-05 | LINC00663 | lincRNA |
| ENSG00000272141.1 | -1.15351 | 1.718502 | 0.000176 | 0.00085 | AL390719.2 | lincRNA |
| ENSG00000248441.5 | -1.15534 | -1.01621 | 0.000781 | 0.003037 | LINC01197 | lincRNA |
| ENSG00000267659.4 | -1.15549 | -1.88615 | 0.00447 | 0.013442 | LINC01482 | lincRNA |
| ENSG00000268912.1 | -1.15691 | 0.246309 | 0.000148 | 0.000729 | AC012313.5 | lincRNA |
| ENSG00000274184.1 | -1.17798 | -1.59401 | 0.000957 | 0.003611 | AC011815.2 | lincRNA |
| ENSG00000234698.1 | -1.18675 | -3.82359 | 0.0441 | 0.089346 | AL161937.1 | lincRNA |
| ENSG00000277550.1 | -1.19182 | -2.7998 | 0.01898 | 0.044682 | AL031651.1 | lincRNA |
| ENSG00000228022.4 | -1.19304 | -3.14111 | 0.014928 | 0.036648 | HCG20 | lincRNA |
| ENSG00000231028.7 | -1.19561 | -1.32397 | 1.54E-05 | 0.000101 | LINC00271 | lincRNA |
| ENSG00000259834.1 | -1.19941 | 0.185553 | 0.000587 | 0.002389 | AL365361.1 | lincRNA |
| ENSG00000226237.1 | -1.20125 | -0.38198 | 0.007398 | 0.02043 | GAS1RR | lincRNA |
| ENSG00000236849.4 | -1.20147 | -2.69058 | 0.04544 | 0.091523 | LINC01474 | lincRNA |
| ENSG00000262370.4 | -1.2161 | -1.18103 | 9.43E-05 | 0.000491 | AC108134.3 | lincRNA |
| ENSG00000258819.1 | -1.22322 | -2.15325 | 0.001701 | 0.005905 | LINC02289 | lincRNA |
| ENSG00000255046.1 | -1.22649 | -1.43283 | 3.50E-05 | 0.000208 | AC069185.1 | lincRNA |
| ENSG00000214548.13 | -1.23826 | 4.07822 | 0.00112 | 0.004117 | MEG3 | lincRNA |
| ENSG00000248508.5 | -1.2397 | 1.266659 | 7.84E-07 | 7.21E-06 | SRP14-AS1 | lincRNA |
| ENSG00000237390.1 | -1.2398 | -3.3746 | 0.010032 | 0.026349 | AL139130.1 | lincRNA |
| ENSG00000279145.1 | -1.24124 | -0.67862 | 2.79E-05 | 0.00017 | AC011912.1 | lincRNA |
| ENSG00000259134.4 | -1.242 | -1.81093 | 0.001656 | 0.005775 | LINC00924 | lincRNA |
| ENSG00000203620.2 | -1.24328 | -3.14951 | 0.01842 | 0.043556 | AL354919.1 | lincRNA |
| ENSG00000204464.6 | -1.24343 | -1.67396 | 0.000235 | 0.001087 | C1orf195 | lincRNA |
| ENSG00000236423.4 | -1.25695 | -0.91117 | 6.42E-05 | 0.000352 | LINC01134 | lincRNA |
| ENSG00000272983.1 | -1.25699 | -1.12044 | 0.000322 | 0.001425 | AL117339.4 | lincRNA |
| ENSG00000224259.4 | -1.25952 | 4.554271 | 0.007398 | 0.02043 | LINC01133 | lincRNA |
| ENSG00000250508.1 | -1.25953 | 1.005294 | 0.038313 | 0.07974 | AP000808.1 | lincRNA |
| ENSG00000246430.5 | -1.26679 | -2.06835 | 0.003131 | 0.00992 | LINC00968 | lincRNA |
| ENSG00000228397.1 | -1.27387 | -3.44812 | 0.010844 | 0.028061 | LINC01635 | lincRNA |
| ENSG00000239482.5 | -1.28691 | -2.48921 | 0.016593 | 0.039945 | AC112487.1 | lincRNA |
| ENSG00000246523.6 | -1.28839 | -1.82956 | 0.000136 | 0.000681 | AP001528.1 | lincRNA |
| ENSG00000180422.3 | -1.30068 | -2.54805 | 0.007212 | 0.020019 | LINC00304 | lincRNA |
| ENSG00000262097.1 | -1.30089 | -1.83715 | 0.006995 | 0.019533 | LINC02185 | lincRNA |
| ENSG00000271204.1 | -1.3063 | -1.64542 | 2.57E-05 | 0.000159 | AC016831.4 | lincRNA |
| ENSG00000232453.4 | -1.31099 | -0.17101 | 0.00022 | 0.001029 | AC105277.1 | lincRNA |
| ENSG00000249487.5 | -1.32228 | -3.41912 | 0.038288 | 0.079709 | LINC01586 | lincRNA |
| ENSG00000260197.1 | -1.32243 | -0.37655 | 0.020143 | 0.046841 | AC010889.1 | lincRNA |
| ENSG00000276071.1 | -1.32384 | -1.53415 | 7.19E-05 | 0.000389 | AC074138.1 | lincRNA |
| ENSG00000274370.1 | -1.32603 | -0.90876 | 0.000131 | 0.000657 | AC130371.2 | lincRNA |
| ENSG00000226251.4 | -1.3292 | -1.53076 | 0.00712 | 0.019817 | AL451060.1 | lincRNA |
| ENSG00000278921.2 | -1.34321 | -0.24255 | 6.36E-05 | 0.000349 | EPB41L4A-DT | lincRNA |
| ENSG00000235097.1 | -1.36882 | -1.25054 | 0.045739 | 0.092011 | LINC00330 | lincRNA |
| ENSG00000226051.5 | -1.37338 | 0.330058 | 0.008875 | 0.023754 | ZNF503-AS1 | lincRNA |
| ENSG00000256973.1 | -1.37356 | -1.54102 | 2.14E-05 | 0.000135 | AC053513.1 | lincRNA |
| ENSG00000255970.1 | -1.37368 | -2.34675 | 0.001131 | 0.004151 | LINC02421 | lincRNA |
| ENSG00000205181.5 | -1.39171 | 0.377662 | 6.49E-05 | 0.000356 | LINC00654 | lincRNA |
| ENSG00000231768.1 | -1.41622 | -1.69998 | 0.000964 | 0.003635 | LINC01354 | lincRNA |
| ENSG00000272129.1 | -1.42364 | -0.07472 | 1.21E-09 | 1.93E-08 | AL359715.3 | lincRNA |
| ENSG00000251432.5 | -1.42681 | 0.258677 | 9.40E-07 | 8.49E-06 | AC108062.1 | lincRNA |
| ENSG00000180861.8 | -1.42705 | 2.072932 | 0.006373 | 0.018062 | LINC01559 | lincRNA |
| ENSG00000234155.1 | -1.44757 | -1.57094 | 0.002289 | 0.007598 | LINC02535 | lincRNA |
| ENSG00000269842.3 | -1.46335 | -2.77295 | 0.007592 | 0.020868 | AC011453.1 | lincRNA |
| ENSG00000273319.1 | -1.46455 | -0.0682 | 1.85E-07 | 1.95E-06 | AC058791.1 | lincRNA |
| ENSG00000267506.4 | -1.47 | -0.48967 | 0.010682 | 0.027735 | AC021683.2 | lincRNA |
| ENSG00000231883.1 | -1.48415 | -3.65721 | 0.041505 | 0.085053 | AL138733.2 | lincRNA |
| ENSG00000228750.4 | -1.49098 | -4.0581 | 0.023704 | 0.053674 | LINC01672 | lincRNA |
| ENSG00000259840.1 | -1.5164 | -2.50638 | 0.000382 | 0.001654 | Z97653.1 | lincRNA |
| ENSG00000261025.1 | -1.52163 | -3.89461 | 0.023003 | 0.052333 | AL445471.2 | lincRNA |
| ENSG00000250903.7 | -1.52975 | 1.815487 | 1.57E-13 | 4.37E-12 | GMDS-DT | lincRNA |
| ENSG00000272264.1 | -1.53443 | -1.41617 | 9.54E-07 | 8.60E-06 | AC009686.2 | lincRNA |
| ENSG00000255774.1 | -1.53762 | -2.4585 | 0.029957 | 0.065213 | AP000439.2 | lincRNA |
| ENSG00000235621.7 | -1.54432 | -2.12704 | 0.001934 | 0.006589 | LINC00494 | lincRNA |
| ENSG00000277022.1 | -1.54858 | -3.27745 | 0.002229 | 0.007425 | AL031663.3 | lincRNA |
| ENSG00000224565.1 | -1.5495 | -3.60518 | 0.008955 | 0.023937 | LINC01754 | lincRNA |
| ENSG00000260855.1 | -1.55643 | -1.36899 | 1.16E-05 | 7.88E-05 | AL591848.4 | lincRNA |
| ENSG00000176515.1 | -1.56037 | -2.44829 | 0.010196 | 0.026688 | AL033381.1 | lincRNA |
| ENSG00000223764.2 | -1.56808 | 0.577167 | 0.000963 | 0.003632 | LINC02593 | lincRNA |
| ENSG00000234880.1 | -1.5698 | -2.00902 | 0.012549 | 0.031732 | LINC00163 | lincRNA |
| ENSG00000254810.1 | -1.57022 | -1.83078 | 6.93E-06 | 4.99E-05 | AP001189.3 | lincRNA |
| ENSG00000214039.7 | -1.57748 | -2.45455 | 0.020133 | 0.046827 | LINC02418 | lincRNA |
| ENSG00000238062.4 | -1.579 | -3.13671 | 0.001396 | 0.004977 | SPATA3-AS1 | lincRNA |
| ENSG00000245468.3 | -1.58748 | -1.60796 | 4.87E-07 | 4.69E-06 | LINC02447 | lincRNA |
| ENSG00000241316.5 | -1.59459 | -0.66856 | 1.23E-08 | 1.64E-07 | SUCLG2-AS1 | lincRNA |
| ENSG00000264727.1 | -1.60817 | -1.18602 | 0.005275 | 0.01542 | AC005725.1 | lincRNA |
| ENSG00000237643.1 | -1.62001 | -1.77496 | 0.000685 | 0.002728 | AL365226.2 | lincRNA |
| ENSG00000230587.1 | -1.64485 | -2.19675 | 5.35E-05 | 0.000301 | LINC02580 | lincRNA |
| ENSG00000267466.1 | -1.64999 | -0.76801 | 0.0062 | 0.017651 | AC021683.1 | lincRNA |
| ENSG00000233559.1 | -1.66429 | -1.32315 | 1.23E-08 | 1.63E-07 | LINC00513 | lincRNA |
| ENSG00000225194.2 | -1.67516 | -0.36142 | 0.00018 | 0.000863 | LINC00092 | lincRNA |
| ENSG00000250986.1 | -1.67541 | 0.34457 | 0.0031 | 0.009837 | LINC02600 | lincRNA |
| ENSG00000267454.4 | -1.68594 | -1.66538 | 1.19E-05 | 8.04E-05 | ZNF582-AS1 | lincRNA |
| ENSG00000244953.1 | -1.68621 | -2.50307 | 0.000117 | 0.000596 | AC087521.1 | lincRNA |
| ENSG00000281383.1 | -1.69507 | 3.545692 | 1.37E-05 | 9.06E-05 | FP671120.4 | lincRNA |
| ENSG00000272079.2 | -1.69645 | -0.88824 | 3.71E-05 | 0.000218 | AC004233.3 | lincRNA |
| ENSG00000205037.2 | -1.70382 | -2.19571 | 1.99E-05 | 0.000126 | AC134312.1 | lincRNA |
| ENSG00000246526.2 | -1.7173 | -1.64393 | 1.25E-09 | 1.99E-08 | LINC002481 | lincRNA |
| ENSG00000261795.1 | -1.72613 | -2.40937 | 0.003832 | 0.011789 | AC093627.7 | lincRNA |
| ENSG00000268628.2 | -1.72725 | 1.455407 | 0.000439 | 0.001859 | AL121761.1 | lincRNA |
| ENSG00000234817.2 | -1.73467 | -2.15287 | 4.22E-07 | 4.11E-06 | AL136309.2 | lincRNA |
| ENSG00000180712.3 | -1.73759 | -1.76443 | 4.04E-08 | 4.91E-07 | LINC02363 | lincRNA |
| ENSG00000267731.1 | -1.74013 | -1.52755 | 2.06E-06 | 1.71E-05 | AC005332.2 | lincRNA |
| ENSG00000233821.1 | -1.74595 | -2.31232 | 7.59E-05 | 0.000408 | ENOX1-AS1 | lincRNA |
| ENSG00000239467.4 | -1.75155 | 0.565117 | 3.22E-05 | 0.000193 | AC007405.3 | lincRNA |
| ENSG00000226777.6 | -1.75236 | 0.226119 | 0.00026 | 0.001184 | FAM30A | lincRNA |
| ENSG00000262585.1 | -1.75472 | -1.72119 | 5.72E-05 | 0.000319 | LINC01979 | lincRNA |
| ENSG00000272505.1 | -1.76324 | -0.35296 | 4.04E-05 | 0.000236 | AC104964.3 | lincRNA |
| ENSG00000251637.5 | -1.76586 | -1.59339 | 9.06E-05 | 0.000473 | AP003716.1 | lincRNA |
| ENSG00000260417.1 | -1.76676 | -2.12963 | 2.24E-09 | 3.41E-08 | AC092127.1 | lincRNA |
| ENSG00000249743.4 | -1.77957 | -2.90399 | 0.012995 | 0.032651 | AC116345.1 | lincRNA |
| ENSG00000233967.5 | -1.84527 | -1.29331 | 1.19E-10 | 2.22E-09 | AL359715.1 | lincRNA |
| ENSG00000259448.2 | -1.8582 | -1.15856 | 5.12E-15 | 1.66E-13 | LINC02352 | lincRNA |
| ENSG00000260645.1 | -1.85876 | -1.07634 | 6.40E-11 | 1.25E-09 | AL359715.2 | lincRNA |
| ENSG00000223403.3 | -1.86019 | -2.4181 | 2.24E-05 | 0.000141 | MEG9 | lincRNA |
| ENSG00000270933.1 | -1.86257 | -1.21675 | 6.91E-09 | 9.55E-08 | AC010719.1 | lincRNA |
| ENSG00000242282.5 | -1.86902 | 0.201814 | 2.21E-14 | 6.72E-13 | AC108488.1 | lincRNA |
| ENSG00000232931.4 | -1.87028 | 2.293655 | 2.52E-10 | 4.51E-09 | LINC00342 | lincRNA |
| ENSG00000236499.2 | -1.89954 | -1.72335 | 1.19E-07 | 1.31E-06 | LINC00896 | lincRNA |
| ENSG00000247157.5 | -1.91051 | -0.08098 | 3.70E-08 | 4.53E-07 | LINC01252 | lincRNA |
| ENSG00000251169.2 | -1.92467 | -2.09384 | 0.000399 | 0.001716 | LINC01843 | lincRNA |
| ENSG00000234492.4 | -1.93271 | -1.90564 | 5.04E-08 | 5.99E-07 | RPL34-AS1 | lincRNA |
| ENSG00000246145.1 | -1.94047 | -1.84195 | 8.65E-06 | 6.09E-05 | RRS1-AS1 | lincRNA |
| ENSG00000233237.5 | -1.94231 | -0.46538 | 1.41E-06 | 1.21E-05 | LINC00472 | lincRNA |
| ENSG00000264968.1 | -1.95642 | -2.6292 | 1.54E-06 | 1.31E-05 | AC090844.2 | lincRNA |
| ENSG00000227388.2 | -1.95764 | -2.40993 | 9.65E-05 | 0.000501 | AL133410.1 | lincRNA |
| ENSG00000229649.1 | -1.96971 | -3.34757 | 0.016447 | 0.039659 | AL133387.2 | lincRNA |
| ENSG00000227502.2 | -1.98529 | -1.65137 | 2.34E-07 | 2.42E-06 | LINC01268 | lincRNA |
| ENSG00000258616.4 | -1.99537 | -2.60897 | 0.029073 | 0.063665 | LINC02303 | lincRNA |
| ENSG00000267461.1 | -1.99936 | -1.58633 | 2.05E-05 | 0.00013 | AC079210.1 | lincRNA |
| ENSG00000250889.2 | -2.00291 | -1.7743 | 2.89E-10 | 5.12E-09 | LINC01336 | lincRNA |
| ENSG00000249464.4 | -2.00873 | -1.38889 | 5.54E-10 | 9.31E-09 | LINC01091 | lincRNA |
| ENSG00000218357.3 | -2.01073 | -1.43747 | 0.004404 | 0.013276 | LINC01644 | lincRNA |
| ENSG00000271420.1 | -2.01476 | -1.85147 | 8.21E-11 | 1.57E-09 | AL109936.2 | lincRNA |
| ENSG00000279516.1 | -2.05103 | -1.53207 | 0.031355 | 0.067676 | FAM230C | lincRNA |
| ENSG00000246379.5 | -2.13449 | -2.87865 | 0.000126 | 0.000638 | AC007495.1 | lincRNA |
| ENSG00000267405.1 | -2.15145 | -1.65336 | 4.76E-05 | 0.000272 | AC005180.1 | lincRNA |
| ENSG00000260704.1 | -2.15203 | -1.53143 | 4.78E-05 | 0.000273 | LINC00543 | lincRNA |
| ENSG00000176728.6 | -2.15388 | 0.12177 | 0.000118 | 0.0006 | TTTY14 | lincRNA |
| ENSG00000274956.2 | -2.1668 | -1.27366 | 0.000587 | 0.002389 | NKAIN3-IT1 | lincRNA |
| ENSG00000236780.4 | -2.16893 | -2.47753 | 1.45E-06 | 1.24E-05 | LINC01829 | lincRNA |
| ENSG00000255693.1 | -2.19858 | -2.53119 | 8.78E-05 | 0.000461 | LINC02389 | lincRNA |
| ENSG00000268555.1 | -2.20573 | -2.41451 | 0.000967 | 0.003643 | AC123912.4 | lincRNA |
| ENSG00000236882.6 | -2.20598 | -1.47817 | 1.73E-06 | 1.46E-05 | LINC01554 | lincRNA |
| ENSG00000236008.1 | -2.21381 | -1.09243 | 8.51E-08 | 9.64E-07 | LINC01814 | lincRNA |
| ENSG00000267505.1 | -2.2147 | -1.48652 | 6.30E-06 | 4.59E-05 | AC005180.2 | lincRNA |
| ENSG00000249669.6 | -2.25525 | 3.483195 | 6.87E-07 | 6.40E-06 | CARMN | lincRNA |
| ENSG00000256288.1 | -2.25544 | -3.13545 | 6.39E-05 | 0.000351 | AC022075.2 | lincRNA |
| ENSG00000260019.1 | -2.25559 | -2.27816 | 0.006781 | 0.019034 | LINC01992 | lincRNA |
| ENSG00000223561.5 | -2.26821 | -0.85503 | 8.23E-07 | 7.54E-06 | AC005165.1 | lincRNA |
| ENSG00000225434.2 | -2.27736 | -0.39652 | 2.86E-09 | 4.27E-08 | LINC01504 | lincRNA |
| ENSG00000277152.1 | -2.30198 | -2.06492 | 2.13E-08 | 2.73E-07 | AC110048.2 | lincRNA |
| ENSG00000230490.2 | -2.36687 | -2.23351 | 1.48E-12 | 3.66E-11 | AL139383.1 | lincRNA |
| ENSG00000259772.5 | -2.39345 | -0.23714 | 3.39E-11 | 6.96E-10 | AC012236.1 | lincRNA |
| ENSG00000268388.4 | -2.43006 | 3.284456 | 1.22E-07 | 1.34E-06 | FENDRR | lincRNA |
| ENSG00000257151.1 | -2.46894 | 1.38995 | 1.82E-09 | 2.81E-08 | PWAR6 | lincRNA |
| ENSG00000224957.4 | -2.48304 | -2.03453 | 4.02E-07 | 3.94E-06 | LINC01266 | lincRNA |
| ENSG00000235601.1 | -2.51114 | -2.42773 | 1.85E-06 | 1.55E-05 | BARX1-DT | lincRNA |
| ENSG00000278035.1 | -2.52046 | -1.54613 | 8.58E-11 | 1.64E-09 | AL121895.2 | lincRNA |
| ENSG00000258760.1 | -2.59091 | -2.84652 | 4.60E-09 | 6.57E-08 | AL355076.2 | lincRNA |
| ENSG00000257647.1 | -2.6109 | -2.18996 | 1.93E-11 | 4.10E-10 | AC124312.2 | lincRNA |
| ENSG00000179082.3 | -2.62216 | -1.71282 | 2.97E-11 | 6.14E-10 | C9orf106 | lincRNA |
| ENSG00000205611.3 | -2.64372 | -1.73903 | 2.21E-11 | 4.64E-10 | LINC01597 | lincRNA |
| ENSG00000269186.1 | -2.68745 | -2.20801 | 1.21E-08 | 1.61E-07 | LINC01082 | lincRNA |
| ENSG00000245330.4 | -2.74357 | -2.75004 | 1.41E-14 | 4.38E-13 | AP005717.1 | lincRNA |
| ENSG00000267259.1 | -2.75675 | 0.066832 | 2.55E-12 | 6.13E-11 | ERVE-1 | lincRNA |
| ENSG00000261069.3 | -2.81778 | -1.72378 | 6.00E-11 | 1.18E-09 | AC124312.3 | lincRNA |
| ENSG00000255474.1 | -2.8638 | -2.49512 | 2.06E-11 | 4.36E-10 | GAU1 | lincRNA |
| ENSG00000245750.6 | -2.90383 | -0.37587 | 8.23E-08 | 9.36E-07 | DRAIC | lincRNA |
| ENSG00000262006.1 | -2.91295 | -2.57004 | 1.42E-06 | 1.22E-05 | AC005920.4 | lincRNA |
| ENSG00000253116.1 | -2.92191 | -3.62004 | 1.26E-07 | 1.38E-06 | AC027698.1 | lincRNA |
| ENSG00000203709.8 | -2.95605 | 2.499063 | 6.89E-19 | 3.05E-17 | MIR29B2CHG | lincRNA |
| ENSG00000253317.1 | -2.95847 | -2.03654 | 5.01E-10 | 8.48E-09 | AC078906.1 | lincRNA |
| ENSG00000231826.4 | -2.97214 | -0.74673 | 9.14E-08 | 1.03E-06 | LINC01819 | lincRNA |
| ENSG00000262188.1 | -3.11383 | -2.19546 | 9.95E-22 | 5.45E-20 | LINC01978 | lincRNA |
| ENSG00000268505.1 | -3.1431 | -1.1869 | 2.46E-09 | 3.72E-08 | AC135012.3 | lincRNA |
| ENSG00000254119.4 | -3.15091 | -3.50558 | 6.10E-06 | 4.46E-05 | AC025524.2 | lincRNA |
| ENSG00000273771.1 | -3.18238 | -2.31817 | 6.67E-20 | 3.19E-18 | AC024337.2 | lincRNA |
| ENSG00000275322.1 | -3.24361 | -2.3844 | 4.40E-29 | 3.93E-27 | AC103746.1 | lincRNA |
| ENSG00000272235.1 | -3.25721 | -2.25196 | 6.99E-19 | 3.09E-17 | AL590438.1 | lincRNA |
| ENSG00000233421.4 | -3.26078 | -2.77521 | 5.47E-16 | 1.93E-14 | LINC01783 | lincRNA |
| ENSG00000222033.1 | -3.29522 | -1.14465 | 1.73E-11 | 3.70E-10 | LINC01124 | lincRNA |
| ENSG00000234571.1 | -3.31289 | -1.91946 | 6.23E-13 | 1.61E-11 | AC239798.2 | lincRNA |
| ENSG00000228037.1 | -3.34408 | -2.36669 | 9.99E-22 | 5.46E-20 | AL139246.3 | lincRNA |
| ENSG00000234350.3 | -3.43448 | -1.36643 | 1.33E-13 | 3.72E-12 | AC007405.1 | lincRNA |
| ENSG00000249388.1 | -3.45149 | -2.19877 | 1.01E-16 | 3.82E-15 | AC023794.2 | lincRNA |
| ENSG00000272894.4 | -3.45854 | 0.9401 | 2.48E-29 | 2.28E-27 | AC004982.2 | lincRNA |
| ENSG00000278484.1 | -3.49624 | -1.30687 | 5.76E-07 | 5.47E-06 | AC010998.3 | lincRNA |
| ENSG00000276772.1 | -3.50381 | -2.68542 | 1.84E-08 | 2.38E-07 | AC025271.4 | lincRNA |
| ENSG00000268416.1 | -3.51908 | -0.7227 | 5.74E-10 | 9.61E-09 | AC010329.1 | lincRNA |
| ENSG00000259275.2 | -3.62892 | -0.18736 | 8.19E-34 | 1.02E-31 | AC087477.2 | lincRNA |
| ENSG00000253844.1 | -3.64224 | -2.59514 | 1.03E-26 | 8.33E-25 | AC064807.2 | lincRNA |
| ENSG00000261863.1 | -3.79865 | -3.23135 | 2.98E-13 | 8.00E-12 | LINC01996 | lincRNA |
| ENSG00000250742.1 | -3.80546 | 3.176455 | 7.09E-40 | 1.33E-37 | LINC02381 | lincRNA |
| ENSG00000228549.3 | -3.85512 | -0.937 | 7.27E-20 | 3.46E-18 | BX284668.2 | lincRNA |
| ENSG00000264016.2 | -3.96136 | -1.84092 | 4.44E-46 | 1.17E-43 | AC015908.3 | lincRNA |
| ENSG00000233569.1 | -3.98511 | -3.20092 | 3.20E-08 | 3.97E-07 | AL161630.1 | lincRNA |
| ENSG00000259359.1 | -4.00483 | -2.92971 | 3.29E-25 | 2.34E-23 | AC012409.1 | lincRNA |
| ENSG00000256969.1 | -4.01696 | -3.50998 | 5.99E-09 | 8.39E-08 | AC007207.2 | lincRNA |
| ENSG00000237361.2 | -4.04873 | -2.07649 | 1.22E-06 | 1.07E-05 | TUSC8 | lincRNA |
| ENSG00000254343.2 | -4.07625 | 0.695707 | 2.78E-36 | 4.05E-34 | AC091563.1 | lincRNA |
| ENSG00000272732.1 | -4.09514 | -0.67555 | 3.53E-36 | 5.12E-34 | AC004982.1 | lincRNA |
| ENSG00000254872.3 | -4.29376 | -0.1348 | 2.60E-15 | 8.63E-14 | AC139749.1 | lincRNA |
| ENSG00000224511.1 | -4.30822 | 0.478094 | 2.59E-19 | 1.18E-17 | LINC00365 | lincRNA |
| ENSG00000242407.1 | -4.37638 | -2.69114 | 4.77E-09 | 6.81E-08 | AC091179.1 | lincRNA |
| ENSG00000259925.1 | -4.37849 | -2.33041 | 2.61E-12 | 6.26E-11 | AC130456.2 | lincRNA |
| ENSG00000274979.1 | -4.43119 | -0.4932 | 4.80E-21 | 2.48E-19 | AC020656.2 | lincRNA |
| ENSG00000263427.1 | -4.66771 | -2.69775 | 7.89E-12 | 1.75E-10 | AC129492.2 | lincRNA |
| ENSG00000229155.1 | -4.73594 | -1.55696 | 9.48E-22 | 5.20E-20 | LINC02038 | lincRNA |
| ENSG00000223823.1 | -4.74306 | -2.12571 | 2.18E-28 | 1.90E-26 | LINC01342 | lincRNA |
| ENSG00000235142.6 | -4.85822 | -0.07241 | 5.18E-21 | 2.67E-19 | LINC02532 | lincRNA |
| ENSG00000260604.2 | -5.17933 | 0.3137 | 1.46E-31 | 1.55E-29 | AL590004.3 | lincRNA |
| ENSG00000255394.4 | -5.41241 | -0.95996 | 7.36E-25 | 5.10E-23 | C8orf49 | lincRNA |
| ENSG00000254290.1 | -5.49894 | -1.51615 | 6.05E-25 | 4.22E-23 | AC124067.4 | lincRNA |
| ENSG00000265369.3 | -5.52859 | -1.14175 | 1.09E-17 | 4.45E-16 | PCAT18 | lincRNA |
| ENSG00000229719.3 | -5.76244 | -0.50825 | 4.93E-60 | 2.62E-57 | MIR194-2HG | lincRNA |
| ENSG00000204876.4 | -5.82053 | -0.14364 | 5.53E-52 | 1.91E-49 | AC021218.1 | lincRNA |
| ENSG00000235049.1 | -6.50573 | -2.08861 | 1.53E-37 | 2.40E-35 | LINC00940 | lincRNA |
| ENSG00000213373.6 | -6.53369 | -0.43379 | 2.14E-55 | 8.93E-53 | LINC00671 | lincRNA |
| ENSG00000263588.1 | -6.66592 | -3.03001 | 4.86E-26 | 3.74E-24 | AC091043.1 | lincRNA |
| ENSG00000256022.4 | -7.04227 | -2.44608 | 6.36E-21 | 3.22E-19 | LINC02411 | lincRNA |
| ENSG00000259974.2 | -9.24889 | 3.95694 | 2.20E-46 | 5.86E-44 | LINC00261 | lincRNA |
| ENSG00000235584.2 | -9.93447 | -0.93492 | 1.92E-53 | 7.12E-51 | AC008268.1 | lincRNA |
